# Transcriptomic and phenotypic analysis of paralogous *spx* gene function in *Bacillus anthracis* Sterne

**DOI:** 10.1002/mbo3.109

**Published:** 2013-07-22

**Authors:** Skye Barendt, Hyunwoo Lee, Cierra Birch, Michiko M Nakano, Marcus Jones, Peter Zuber

**Affiliations:** 1 Division of Environmental and Biomolecular SystemsInstitute of Environmental Health, Oregon Health and Science UniversityBeaverton, Oregon; 2 Center for Pharmaceutical Biotechnology, University of Illinois-ChicagoChicago, Illinois; 3 Infectious Disease Group, J. Craig Venter InstituteRockville, Maryland

**Keywords:** *Bacillus anthracis*, oxidative stress, SpxA1, SpxA2, transcriptomic

## Abstract

Spx of *Bacillus subtilis* is a redox-sensitive protein, which, under disulfide stress, interacts with RNA polymerase to activate genes required for maintaining thiol homeostasis. Spx orthologs are highly conserved among low %GC Gram-positive bacteria, and often exist in multiple paralogous forms. In this study, we used *B. anthracis* Sterne, which harbors two paralogous *spx* genes, *spxA1* and *spxA2,* to examine the phenotypes of *spx* null mutations and to identify the genes regulated by each Spx paralog. Cells devoid of *spxA1* were sensitive to diamide and hydrogen peroxide, while the *spxA1 spoxA2* double mutant was hypersensitive to the thiol-specific oxidant, diamide. *Bacillus anthracis* Sterne strains expressing *spxA1DD* or *spxA2DD* alleles encoding protease-resistant products were used in microarray and quantitative real-time polymerase chain reaction (RT-qPCR) analyses in order to uncover genes under SpxA1, SpxA2, or SpxA1/SpxA2 control. Comparison of transcriptomes identified many genes that were upregulated when either SpxA1DD or SpxA2DD was produced, but several genes were uncovered whose transcript levels increased in only one of the two SpxADD-expression strains, suggesting that each Spx paralog governs a unique regulon. Among genes that were upregulated were those encoding orthologs of proteins that are specifically involved in maintaining intracellular thiol homeostasis or alleviating oxidative stress. Some of these genes have important roles in *B. anthracis* pathogenesis, and a large number of upregulated hypothetical genes have no homology outside of the *B. cereus/thuringiensis* group. Microarray and RT-qPCR analyses also unveiled a regulatory link that exists between the two *spx* paralogous genes. The data indicate that *spxA1* and *spxA2* are transcriptional regulators involved in relieving disulfide stress but also control a set of genes whose products function in other cellular processes.

*Bacillus anthracis* harbors two paralogs of the global transcriptional regulator of stress response, SpxA. SpxA1 and SpxA2 contribute to disulfide stress tolerance, but only SpxA1 functions in resistance to peroxide. Transcriptome analysis uncovered potential SpxA1 and SpxA2 regulon members, which include genes activated by both paralogs. However, paralog-specific gene activation was also observed. Genes encoding glutamate racemase, CoA disulfide reductase, and products functioning in bacillithiol biosynthesis, are among the genes activated by the SpxA paralogs.

## Introduction

*Bacillus anthracis* is a spore-forming, nonmotile Gram-positive bacterium that is the causative agent of the zoonotic infectious disease, anthrax (Beyer and Turnbull 2009). It is an effective pathogen because the infectious agent of anthrax is the metabolically dormant and highly resistant spore. Upon ingestion by a professional phagocytic cell (e.g., activated macrophage), the spore undergoes germination and outgrowth to generate a vegetative cell that is capable of reproduction within the infected host, as it produces plasmid-encoded toxins and protective capsule material for evading immune capture and destruction (Fouet et al. 1999; Koehler 2009; Moayeri and Leppla 2009; Tournier et al. 2009). Germination and outgrowth in the macrophage takes place within a hostile environment, made so by the phagocyte's oxidative burst, which generates a toxic combination of reactive oxygen species (ROS), nitric oxide (NO), and hypochlorous acid (HOCl), as well as phospholipase, and antimicrobial peptides (Piris-Gimenez et al. 2005; Passalacqua and Bergman 2006; Passalacqua et al. 2006; Dawson and Liu 2008; Welkos et al. 2011). Successful establishment of infection involves mechanisms of oxidant resistance (Shatalin et al. 2008; Welkos et al. 2011). Such systems in bacteria are activated by encounters with a variety of toxic agents, not only components of the oxidative burst, but antibiotics and other chemical and physical insults (Gusarov et al. 2009; Mols and Abee 2011).

Much of what is known about the oxidative stress response in Bacilli has come from studies of *Bacillus subtilis*, a nonpathogen, which is a model genetic system used in studying Gram-positive physiology and the bacterial response to harsh conditions. Recent studies of oxidant sensitivity indicated that *B. subtilis* is more sensitive to the lethal effects of peroxide and superoxide-generating agents than is *B. anthracis* (Pohl et al. 2011). The findings suggest that more robust processes of oxidant detoxification and tolerance have evolved in the pathogen, which is in keeping with its developmental cycle involving reproduction within phagocytic hosts. Several regulatory proteins govern the oxidative stress response in *B. subtilis*, including the peroxide sensor PerR (Lee and Helmann 2006), organic hydroperoxide-sensing MarR family protein, OhrR (Fuangthong et al. 2001), HypR, which senses thiol-reactive HOCl stress (Palm et al. 2012), and SpxA (Zuber 2004). All of these proteins have orthologs in *B. anthracis* (PerR [BA0537], HypR [BA3379], OhrR [BA4699]). Both species possess the general stress response sigma subunit, σ^B^, which controls a large regulon that becomes activated by starvation and reduced energy-generating capability, as well as by stress brought about through encounters with toxic chemical and physical agents (Hecker et al. 2007; van Schaik et al. 2007). Peroxide induces the σ^B^ regulon in *B. subtilis*, but σ^B^ is poorly activated by peroxide stress in *B. anthracis*, which is likely due to the different regulatory architectures that operate in the two organisms (Pohl et al. 2011; Tu et al. 2012b). The response of *B. anthracis* to superoxide stress, which is likely encountered within the infected macrophage, is the elevated expression of genes within the Fur (Ferric uptake regulator) regulon specifying iron uptake mechanisms, which is also observed in *B. subtilis* (Mostertz et al. 2004; Passalacqua et al. 2007; Tu et al. 2012a). The response seems maladaptive as it would expose macromolecules to potentially damaging, hydroxyl radicals generated by the Fenton reaction (Liochev and Fridovich 1999; Imlay 2008). Superoxide is known to cause decomposition of enzyme iron centers, which could trigger an iron starvation response through inactivation of the Fur transcriptional regulator and stimulation of the Fur regulon (Mostertz et al. 2004; Passalacqua et al. 2007). However, it has been proposed that superoxide is a germination signal for *B. anthracis* spores (Fisher and Hanna 2005), and accelerated iron uptake during subsequent outgrowth may assist in coping with the iron-poor environment that characterizes the infected host. In contrast to *B. subtilis*, the response of *B. anthracis* to superoxide is limited involving around 40 genes, which might reflect the signaling role of superoxide in *B. anthracis* infection rather than an agent of general stress generation (Tu et al. 2012b).

Peroxide stress induces over 200 genes in *B. anthracis* that specify a variety of activities related to detoxification, macromolecular damage repair, and disposal of damaged protein (Pohl et al. 2011). Thus, genes encoding DNA repair enzymes, and other members of the LexA regulon, are induced. Genes functioning in redox homeostasis, including those encoding components of the bacillithiol biosynthesis pathway, are also activated. A significant change in the *B. anthracis* transcriptome is evident from the observation that several genes encoding regulatory proteins are activated after peroxide treatment. These include genes specifying the PerR and SpxA transcriptional regulators (Bergman et al. 2007; Passalacqua et al. 2007; Pohl et al. 2011).

SpxA is a global regulator of the stress response that is activated upon thiol stress. Over 250 genes, or 144 operons, are controlled by SpxA in *B. subtilis* (Nakano et al. 2003; Rochat et al. 2012). The protein is a direct transcriptional activator through an interaction with the RNA polymerase alpha subunit in *B. subtilis* (Nakano et al. 2005; Newberry et al. 2005). Among the genes activated by SpxA are those required to establish the reduced state of thiols in the cytoplasm. Such genes include those that encode thioredoxin (*trxA*), thioredoxin reductase (*trxB*), methionine sulfoxide reductase (You et al. 2008), enzymes required for synthesis of the low-molecular-weight thiol bacillithiol (Gaballa et al. 2010; Chi et al. 2011; Gaballa and Helmann 2011), and genes that function in cysteine biosynthesis (Nakano et al. 2003; Choi et al. 2006; Zuber et al. 2011; Rochat et al. 2012). A null mutation in *spx* causes sensitivity to thiol reactive compounds, partial cysteine auxotrophy, and causes disruption of iron uptake control. The *spx* gene is under complex transcriptional control that is responsive to stress caused by variety of physical and chemical agents (Helmann et al. 2001; Petersohn et al. 2001; Jervis et al. 2007; Leelakriangsak et al. 2007; Eiamphungporn and Helmann 2008). In *B. subtilis* and in other Gram-positive species, *spxA* is transcriptionally induced by mechanisms responsive to cell envelope stress. SpxA can undergo stress-induced thiol oxidation of a CxxC disulfide center, which is necessary for its productive interaction with RNA polymerase (Nakano et al. 2005). The SpxA protein is under proteolytic control that requires the ATP-dependent protease, ClpXP, and a substrate recognition factor, YjbH (Larsson et al. 2007; Garg et al. 2009). SpxA, as might be expected given its role in regulating the oxidative stress response, has been found to be required for virulence in Streptococci and *Enterococcus* (Kajfasz et al. 2010, 2012; Chen et al. 2012).

**Figure 1:**
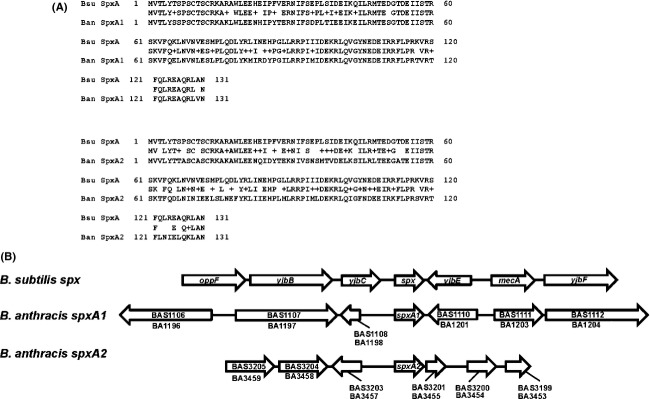
*Bacillus anthracis* paralogs, SpxA1 and SpxA2, are orthologs of *B. subtilis* Spx. (A) Comparison of the primary structures of SpxA1 and SpxA2 in *B. anthracis* with Spx in *B. subtilis*. ClpXP protease-resistant forms of Spx, SpxA1DD and SpxA2DD, were constructed by replacing the two C-terminal residues with DD. (B) Synteny between the paralogous *spx* genes in *B. anthracis* and *spx* in *B. subtilis*. *Bacillus anthracis* Sterne nomenclature is used. BAS1110 and BAS1111 encode, respectively, YjbE and MecA orthologs. Also shown are the gene designations according to the *B. anthracis* Ames nomenclature.

Several of the low %GC Gram-positive bacteria possess multiple paralogs of Spx (Veiga et al. 2007; Turlan et al. 2009). The genome of *B. anthracis* bears two *spxA* genes, *spxA1* and *spxA2* (Fig. 1A). The *spxA1*-linked genes show syntenic similarity to those in *B. subtilis*, but *B. anthracis* also contains an additional paralogous *spx* gene in a part of the genome that shows no synteny with *B. subtilis* (Fig. 1B). Previous transcriptomic studies have shown that *spxA1* is expressed in early log phase of a *B. anthracis* culture, while *spxA2* transcript is detected during stationary phase (Bergman et al. 2006). The *spxA2* gene is one of the most highly induced genes in *B. anthracis* cells following germination in the host macrophage (Bergman et al. 2007). The differences in expression patterns exhibited by the two paralogous genes suggest differences in their roles within the stress response network of *B. anthracis*.

To uncover the roles of the two paralogous genes of *spxA* in *B. anthracis*, a study was conducted to examine the phenotype conferred by null mutations in the genes encoding the two paralogs and to identify the genes that are regulated by SpxA1 and SpxA2. The work reported herein shows that the two paralogous SpxA proteins oversee two large overlapping regulons. While both function in the oxidative stress response, SpxA1 plays an essential role in the bacterium's resistance to peroxide and disulfide stress.

## Experimental Procedures

### Bacterial strains and growth conditions

Bacterial strains and plasmids are listed in Table 1. The *B. anthracis* Sterne strains used in this study are derivatives of 7702 (pXO1^+^ pXO2^−^) and, in most cases, were grown in Lysogeny Broth (LB) medium at 37°C or sporulation medium (SM) at 30°C (Barua et al. 2009). The *B. subtilis* strains used in this study are derivatives of JH642 (*trpC2 pheA1*) and were grown at 37°C in Difco sporulation medium (DSM) unless otherwise indicated. *Escherichia coli* DH5α was used for plasmid construction and was grown at 37°C in LB liquid or on LB solid medium containing 1.2% agar (Difco, BD Biosciences, San Jose, CA). Appropriate antibiotics were added: *B. subtilis*, 75 μg/mL spectinomycin, 1 μg/mL erythromycin/25 μg/mL lincomycin, 5 μg/mL kanamycin; *B. anthracis*, 100 μg/mL streptomycin, 100 μg/mL spectinomycin, 20 μg/mL kanamycin; *E. coli*, 100 μg/mL ampicillin, 100 μg/mL spectinomycin, 20 μg/mL kanamycin.

**Table:** Bacterial strains and plasmids used in this study

Strain	Genotype	Derivation	Antibiotic resistance	Source
*Bacillus anthracis* Sterne 7702 derivative strains				
*B. anthracis* Sterne 7702	Parent		None	Pasteur Institute Cataldi et al. (1990)
*B. anthracis* Sterne 7702 SR1	Streptomycin resistant	Streptomycin-resistant isolate of *B. anthracis* Sterne 7702	Strep	This study
ORB7863	ICE*Bs1*::P_spank(hy)_-*spxA1DD*	ICE*Bs1*::pCSZ35 (P_spank(hy)_-*spxA1DD*) × *B. anthracis* Sterne 7702 SR1	Spec, Strep	This study
ORB7864	ICE*Bs1*::P_spank(hy)_-*spxA2DD*	ICE*Bs1*::pCSZ36 (P_spank(hy)_-*spxA2DD*) × *B. anthracis* Sterne 7702 SR1	Spec, Strep	This study
ORB8092	ICE*Bs1*	pJMA402 (ICE*Bs1*) × *B. anthracis* Sterne 7702 SR1	Spec, Strep	This study
ORB8115	Δ*spxA2*	pSB3 (Δ*spxA2*) × *B. anthracis* Sterne 7702 SR1	Strep	This study
ORB8170	Δ*spxA1*	pSB2 (Δ*spxA1*) × *B. anthracis* Sterne 7702	None	This study
ORB8285	Δ*spxA1* Δ*spxA2*	pSB3 (Δ*spxA2*) × ORB8170	None	This study
ORB8390	Δ*spxA2* ICE*Bs1*::P_spank(hy)_-*spxA1DD*	ICE*Bs1*::pCSZ35 (P_spank(hy)_-*spxA1DD*) × ORB8115	Spec, Strep	This study
ORB8391	Δ*spxA2* ICE*Bs1*::P_spank(hy)_-*spxA2DD*	ICE*Bs1*::pCSZ36 (P_spank(hy)_-*spxA2DD*) × ORB8115	Spec, Strep	This study
ORB8398	Δ*spxA1*	Streptomycin-resistant isolate of ORB8170	Strep	This study
ORB8404	Δ*spxA1* ICE*Bs1*::P_spank(hy)_-*spxA1DD*	ICE*Bs1*::pCSZ35 (P_spank(hy)_-*spxA1DD*) × ORB8170	Spec, Strep	This study
ORB8405	Δ*spxA1* ICE*Bs1*::P_spank(hy)_-*spxA2DD*	ICE*Bs1*::pCSZ36 (P_spank(hy)_-*spxA2DD*) × ORB8170	Spec, Strep	This study
ORB8438	Δ*spxA2*	pSB3 (Δ*spxA2*) × *B. anthracis* Sterne 7702	None	This study
ORB8481	Δ*spxA1* Δ*spxA2*	Streptomycin-resistant isolate of ORB8285	Strep	This study
ORB8485	Δ*spxA1* Δ*spxA2* ICE*Bs1*::P_spank(hy)_-*spxA1DD*	ICE*Bs1*::pCSZ35 (P_spank(hy)_-*spxA1DD*) × ORB8481	Spec, Strep	This study
ORB8486	Δ*spxA1* Δ*spxA2* ICE*Bs1*::P_spank(hy)_-*spxA2DD*	ICE*Bs1*::pCSZ36 (P_spank(hy)_-*spxA2DD*) × ORB8481	Spec, Strep	This study
*Bacillus subtilis* JH642 (*trpC2 pheA1*) derivative strains				
JH642	Parent		None	J. Hoch
JMA475	*cgcD*::P_spank(hy)_*-rapI kan458*		Kan	A. Grossman Auchtung et al. (2005)
ORB3834	Δ*spx::neo*	Δ*spx::neo* × JH642	Neo	Nakano et al. (2001)
ORB7262	Δ*spx::neo* *thrC*::P_*BA5387*_*-lacZ*	*thrC*::pDYR9 (P_*BA5387*_*-lacZ*) × ORB8384	Neo, Erm	This study
ORB7854	ICE*Bs1*::P_spank(hy)_-*spxA1DD*	ICE*Bs1*::pCSZ35 (P_spank(hy)_-*spxA1DD*) × JH642	Spec	This study
ORB7860	ICE*Bs1*::P_spank(hy)_-*spxA2DD*	ICE*Bs1*::pCSZ36 (P_spank(hy)_-*spxA2DD*) × JH642	Spec	This study
ORB7861	ICE*Bs1*::P_spank(hy)_-*spxA1DD cgcD*::P_spankhy_*-rapI kan458*	JMA475 genomic DNA × ORB7854	Spec, Kan	This study
ORB7862	ICE*Bs1*::P_spank(hy)_-*spxA2DD cgcD*::P_spank(hy)_*-rapI kan458*	JMA475 genomic DNA × ORB7860	Spec, Kan	This study
ORB7871	Δ*spx* *thrC*::P_*BA5387*_*-lacZ* *amyE*::P_spank(hy)_-*spxA1DD*	*amyE*::pMMN818 (P_spank(hy)_-*spxA1DD*) × ORB7262	Neo, Erm, Spec	This study
ORB7872	Δ*spx* *thrC*::P_*BA5387*_*-lacZ* *amyE*::P_spank(hy)_-*spxA2DD*	*amyE*::pMMN819 (P_spank(hy)_-*spxA2DD*) × ORB7262	Neo, Erm, Spec	This study
ORB8343	Δ*spx::neo* *thrC*::P_*BA0847*_-*lacZ*	*thrC*::P_*BA0847*_-*lacZ* × ORB3834	Neo, Erm	This study
ORB8344	Δ*spx::neo* *thrC*::P_*BA1119*_-*lacZ*	*thrC*::P_*BA1119*_-*lacZ* × ORB3834	Neo, Erm	This study
ORB8354	Δ*spx::neo* *thrC*::P_*BA1951*_-*lacZ*	*thrC*::P_*BA1951*_-*lacZ* × ORB3834	Neo, Erm	This study
ORB8356	Δ*spx::neo* *thrC*::P_*BA5387*_-*lacZ*	*thrC*::P_*BA5387*_-*lacZ* × ORB3834	Neo, Erm	This study
ORB8359	Δ*spx::neo* *thrC*::P_*BA0847*_-*lacZ* *amyE*::P_spank(hy)_-*spxA1DD*	*amyE*:: P_spank(hy)_-*spxA1DD* × ORB8343	Neo, Erm, Spec	This study
ORB8360	Δ*spx::neo* *thrC*::P_*BA0847*_-*lacZ* *amyE*:: P_spank(hy)_-*spxA2DD*	*amyE*:: P_spank(hy)_-*spxA2DD* × ORB8343	Neo, Erm, Spec	This study
ORB8361	Δ*spx::neo* *thrC*::P_*BA1119*_-*lacZ* *amyE*:: P_spank(hy)_-*spxA1DD*	*amyE*:: P_spank(hy)_-*spxA1DD* × ORB8344	Neo, Erm, Spec	This study
ORB8362	Δ*spx::neo* *thrC*::P_*BA1119*_-*lacZ* *amyE*:: P_spank(hy)_-*spxA2DD*	*amyE*:: P_spank(hy)_-*spxA2DD* × ORB8344	Neo, Erm, Spec	This study
ORB8363	Δ*spx::neo* *thrC*::P_*BA1951*_-*lacZ* *amyE*:: P_spank(hy)_-*spxA1DD*	*amyE*:: P_spank(hy)_-*spxA1DD* × ORB8354	Neo, Erm, Spec	This study
ORB8364	Δ*spx::neo* *thrC*::P_*BA1951*_-*lacZ* *amyE*:: P_spank(hy)_-*spxA2DD*	*amyE*:: P_spank(hy)_-*spxA2DD* × ORB8354	Neo, Erm, Spec	This study
ORB8367	Δ*spx::neo* *thrC*::P_*BA5387*_-*lacZ* *amyE*:: P_spank(hy)_-*spxA1DD*	*amyE*:: P_spank(hy)_-*spxA1DD* × ORB8356	Neo, Erm, Spec	This study
ORB8368	Δ*spx::neo* *thrC*::P_*BA5387*_-*lacZ* *amyE*:: P_spank(hy)_-*spxA2DD*	*amyE*:: P_spank(hy)_-*spxA2DD* × ORB8356	Neo, Erm, Spec	This study
ORB8373	Δ*spx::neo* *thrC*::P_*BA1200*_-*lacZ*	*thrC*::P_*BA1200*_-*lacZ* × ORB3834	Neo, Erm	This study
ORB8380	Δ*spx::neo* *thrC*::P_*spxA1*_-*lacZ* *amyE*:: P_spank(hy)_-*spxA1DD*	*amyE*:: P_spank(hy)_-*spxA1DD* × ORB8373	Neo, Erm, Spec	This study
ORB8381	Δ*spx::neo* *thrC*::P_*BA1200*_-*lacZ* *amyE*:: P_spank(hy)_-*spxA2DD*	*amyE*:: P_spank(hy)_-*spxA2DD* × ORB8373	Neo, Erm, Spec	This study
ORB8389	*thrC*::P_*BA3868*_-*lacZ*	*thrC*::P_*BA3868*_-*lacZ* × ORB3834	Neo, Erm	This study
ORB8396	*thrC*::P_*BA3868*_-*lacZ* *amyE*:: P_spank(hy)_-*spxA2DD*	*amyE*:: P_spank(hy)_-*spxA2DD* × ORB8389	Neo, Erm, Spec	This study
ORB8397	*thrC*::P_*BA3868*_-*lacZ* *amyE*:: P_spank(hy)_-*spxA1DD*	*amyE*:: P_spank(hy)_-*spxA1DD* × ORB8389	Neo, Erm, Spec	This study
Plasmid	Genotype			Selection				
pCSZ35	pJMA402::P_spank(hy)_-*spxA1DD*			Spec				
pCSZ36	pJMA402::P_spank(hy)_-*spxA2DD*			Spec				
pSB2	pRP1028::Δ*spxA1*			Spec				
pSB3	pRP1028::Δ*spxA2*			Spec				
pSB10	pPROEX-1::*spxA2*			Amp				
pMMN818	pDR111::P_spank(hy)_-*spxA1DD*			Spec				
pMMN819	pDR111::P_spank(hy)_-*spxA2DD*			Spec				
pDG793	*thrC* integration vector with promoter-less *lacZ* reporter			Erm Guerout-Fleury et al. (1996)				
pSS1827	Conjugation helper strain			Amp				
pSS4332	Harbors I-SceI gene and cyan fluorescent protein reporter construct			Kan				
pRP1028	Cloning vector for conjugation			Spec				
pJMA402	Cloning vector for ICE*Bs1* conjugation			Spec				
pDR111	*amyE* integration vector with P_spank(hy)_ promoter			Spec				
pPROEX-1	*E. coli* expression vector			Amp				
pDRY9	*thrC* integration plasmid carrying *B. subtilis trxB* (−115 to +47)-*lacZ*			Erm				

Spec, spectinomycin; Strep, streptomycin; Kan, kanamycin; Amp, ampicillin; Erm, erythromycin; Neo, neomycin.

### Construction of *B. anthracis spx* mutants

Two kilobytes DNA amplicons containing 1 kb up- and 1 kb downstream flanking DNA regions of either *spxA1* or *spxA2* were generated by overlapping fusion polymerase chain reaction (PCR) using chromosomal DNA from *B. anthracis* 7702 (see Table S6 for primer sequences). The DNA amplicons were digested with HindIII and KpnI, purified, and ligated with similarly digested and purified pRP1028 using T4 DNA ligase to construct pSB2 (pRP1028::Δ*spxA1)* and pSB3 (pRP1028::Δ*spxA2)*. The plasmids containing in-frame deletion mutations of *spxA1* and *spxA2 w*ere introduced into *B. anthracis* 7702 (or a derivative 7702 SR1) using a markerless allelic replacement technique developed by Janes and Stibitz (Janes and Stibitz 2006). *Escherichia coli* DH5α strains harboring pRP1028::Δ*spxA1* (pSB2) or pRP1028::Δ*spxA2* (pSB3) were conjugated at room temperature for 24 h with *B. anthracis* Sterne 7702 in the presence of an *E. coli* helper strain containing pSS1827 on brain-heart infusion (BHI) plates lacking antibiotic. The transconjugate diploid intermediates (Δ*spx spx*^*+*^) were conjugated for a second time at 37°C with *E. coli* strains containing pSS4332 (encoding nuclease I-SceI) and pSS1827. Conjugants were screened for the loss of pRP1028 and were subsequently subjected to several rounds of plating on BHI agar with incubation at 37°C to eliminate plasmid pSS4332. Spectinomycin and kanamycin sensitive isolates were obtained and screened by PCR (using primers that anneal outside of the sequence used in the construction of the mutant) and nucleotide sequence analysis of these PCR products was performed to ensure that the final Δ*spxA1* (ORB8170) and Δ*spxA2* (ORB8115 and ORB8438) constructs were correct.

### Construction of *B. anthracis* strains that produce ClpXP-insensitive SpxA1 and SpxA2

The *spxA1DD* and *spxA2DD* genes were amplified by PCR from *B. anthracis* 7702 chromosomal DNA using oMN10-535 and oMN10-536 (for *spxA1DD*) or oMN10-537 and oMN10-528 (for *spxA2DD*). The PCR products were digested with HindIII and SphI and ligated into pDR111 (Britton et al. 2002) digested with the same enzymes. The resultant plasmids pMMN818 and pMMN819 contain *spxA1DD* and *spxA2DD* under the control of an IPTG (isopropyl β–D-1-thiogalactopyranoside)-inducible P_spank(hy)_ promoter. pMMN818 and pMMN819 were digested with BamHI and were filled-in using T4 DNA polymerase and dNTPs, followed by EcoRI digestion. The released 2.3-kb fragment containing *lacI* and *spxADD* was subcloned into pJMA402 (kindly provided by C. Lee and A. D. Grossman), digested with EcoRI and HincII to generate pCSZ35 and pCSZ36. pCSZ35 and pCSZ36 were used to transform *B. subtilis* JH642 with selection for spectinomycin resistance, resulting in strains ORB7854 and ORB7860, respectively. Donor strains used for ICE*Bs1*-mediated conjugation, namely ORB7861 and ORB7862, were generated by transformation of ORB7854 and ORB7860 with chromosomal DNA isolated from JMA475 with selection for kanamycin resistance. ICE*Bs1*-mediated conjugation was carried out as previously described (Auchtung et al. 2005) using ORB7861 or ORB7862 as a donor and 7702 SR1 as a recipient with selection for both streptomycin and spectinomycin resistance. *spxA1DD* and *spxA2DD* in *B. anthracis* conjugants ORB7863 and ORB7864 were verified by sequence analysis of the PCR product amplified from the chromosomal DNA using oligonucleotides spac-up and spac-down (Table S6).

### Culture preparation and RNA extraction

*Bacillus anthracis* Sterne 7702 SR1 and overexpression derivatives bearing P_spank(hy)_-*spxA1DD* (ORB7863), P_spank(hy)_-*spxA2DD* (ORB7864), and empty vector control strain bearing P_spank(hy)_-empty (ORB8092), were grown in 60 mL LB 37°C, 200 rpm until mid-exponential phase (OD_600_ 0.2–0.4) and then split into two equal volumes. One millimoles per liter IPTG was added to one set of cultures and the cultures were incubated for an additional 15 or 45 min, after which the pellets from 10 mL culture samples were harvested by centrifugation (5180*g*, 4°C, 10 min.), and frozen at −80C until use. For diamide-treated cultures, *B. anthracis* Sterne 7702 and deletion derivatives, Δ*spxA1* (ORB8170) and Δ*spxA1* Δ*spxA2* (ORB8285), were grown in 40 mL LB at 37°C, 200 rpm until mid-exponential phase (OD_600_ 0.2–0.4) and split into two equal volumes. One mmol/L diamide (freshly prepared, Sigma-Aldrich, St. Louis, MO) was added to one set of cultures and, following incubation for an additional 20 min, were harvested by centrifugation, as above. RNA was purified as previously described (Igo and Losick 1986).The resulting RNA concentrations were measured by ultraviolet spectrophotometry. RNA quality was assessed by measuring the ratio of absorbance at 260 nm to absorbance at 280 nm, as well as by visualization in agarose gels.

### Microarray design, data collection, and data analysis

cDNA for microarray experiments were generated by adding 2 μg of total RNA in a mixture containing 6 μg of random hexamers (Life Technologies, Carlsbad, CA), 0.01 mol/L dithiothreitol, an aminoallyl-deoxynucleoside triphosphate mixture containing 25 mmol/L each dATP, dCTP, and dGTP, 15 mmol/L dTTP, and 10 mmol/L amino-allyl-dUTP (aa-dUTP) (Sigma), reaction buffer, and 400 units of SuperScript III reverse transcriptase (RT) (Life Technologies) at 42°C overnight. The RNA template then was hydrolyzed by adding NaOH and ethylenediaminetetraacetic acid to a final concentration of 0.2 and 0.1 mol/L, respectively, and incubating at 70°C for 15 min. Unincorporated aa-dUTP was removed with a Minelute column (Qiagen, Gaithersburg, MD). The probe was eluted with a phosphate elution buffer (4 mmol/L potassium phosphate buffer, pH 8.5, in ultrapure water), dried, and resuspended in 0.1 mol/L sodium carbonate buffer (pH 9.0). To couple the amino-allyl cDNA with fluorescent labels, normal human serum-Cy3 or normal human serum-Cy5 (GE Healthcare Biosciences, Pittsburgh, PA) was added at room temperature for 1 h. Uncoupled label was removed using a Minelute column (Qiagen). Aminosilane-coated slides printed with a set of 5823 oligonucleotides representing all open reading frame sequences of *B. anthracis* Ames A2012 (www.jcvi.org) were prehybridized in 5× SSC (1× SSC is 0.15 mol/L NaCl plus 0.015 mol/L sodium citrate) (Life Technologies), 0.1% sodiumdodecyl sulfate (SDS), and 1% bovine serum albumin at 42°C for 60 min. The slides then were washed at room temperature with distilled water, dipped in isopropanol, and allowed to dry. Equal volumes of the appropriate Cy3- and Cy5-labeled probes were combined, dried, and then resuspended in a solution of 40% formamide, 5× SSC, and 0.1% SDS. Resuspended probes were heated to 95ºC prior to hybridization. The probe mixture then was added to the microarray slide and allowed to hybridize overnight at 42°C. Hybridized slides were washed sequentially in solutions of 1× SSC-0.2% SDS, 0.1× SSC-0.2% SDS, and 0.1× SSC at room temperature, then dried in air, and scanned with an Axon GenePix 4000 scanner (Molecular Devices, Sunnyvale, CA). All wash buffers were supplemented with 1 mL of 0.1 mol/L dithiothreitol per liter of wash buffer. Individual TIFF images from each channel were analyzed with TIGR Spotfinder (available at [www.tm4.org]). Microarray data were normalized by LOWESS normalization and with in-slide replicate analysis using TM4 software MIDAS (available at www.tm4.org). The array design is available at Gene Expression Omnibus (www.ncbi.nlm.nih.gov/geo) with accession number GPL10188.

### Quantitative real-time polymerase chain reaction analysis

cDNA was prepared from each RNA sample using random primers and Invitrogen SuperScript III RT (Life Technologies) as per the manufacturer's protocol. Triple or quadruple technical replicates were performed for each quantitative real-time polymerase chain reaction (RT-qPCR) assay, from either two (for microarray validation) or three (for diamide treatment) independently isolated RNA samples, in a 96-well plate using an ABI Prism Step-One Plus with Step-One Plus (Life Technologies) Software version 2.0 sequence detection system, an annealing temperature of 58°C, and extension at 72°C for 1 min for 40 cycles. Primer sequences (Table S6) were designed to specifically amplify a 100–250-bp portion of each transcript of interest (see Table S6). The amplification efficiencies were roughly equivalent across all primer sets (E% = 80–110). Control reactions (cDNA reactions lacking RT) were performed to verify that no genomic DNA contamination was present (that is, the threshold cycle [*C*_T_] for detection in the control without RT was above 35). Normalization of *C*_T_ values was done relative to the signal obtained from reactions amplifying a portion of the *gatB*/Yqey (BA4533) transcript; the expression level of this gene has been shown previously to remain stable across the entire *B. anthracis* life cycle (Reiter et al. 2011), and the microarray experiments described in this study suggest that its expression levels change less than twofold when increased amounts of SpxA1 or SpxA2 are present (see Tables S1, S2). Fold-changes for microarray validation studies were obtained by taking the ratio of +IPTG/−IPTG samples after normalization to *gatB*/Yqey. Transcripts (ng) were directly reported for RT-qPCR assays of diamide-treated cultures following normalization to the *gatB*/Yqey endogenous control.

### Promoter-*lacZ* strain construction and β-galactosidase assay

Amplified promoter regions, generated by primers listed in Table S6, were digested with EcoR1 and BamHI, ligated into pDG793 (harboring the *lacZ* reporter construct, [Guerout-Fleury et al. 1996]) and propagated by transformation of competent DH5α cells. Plasmids containing the correct insert were introduced by transformation into ORB3834 (*B. subtilis* JH642 Δ*spx::neo*) with selection for Erm resistance and screening for *thrC* (threonine auxotrophy). These resulting strains were transformed with pMMN818 (P_spank(hy)_-*spxA1*DD) or pMMN819 (P_spank(hy)_-*spxA2*DD) (see Table 1) to obtain the final strains (e.g., *B. subtilis* JH642 Δ*spx::neo* [Neo] P_*exoA*_-*lacZ* [Erm] *amyE*::P_spank(hy)_-*spxA1DD* [Spec]) used in β-galactosidase assays. Strains bearing various promoters (see Table 1) fused to a *lacZ-*reporter were grown at 37°C overnight on DSM supplemented with appropriate antibiotics. Assays of β-galactosidase in DSM cultures were performed as previously described (Nakano et al. 1998); activity was calculated in Miller units (Miller 1972).

### Western blot analysis

**Figure 2:**
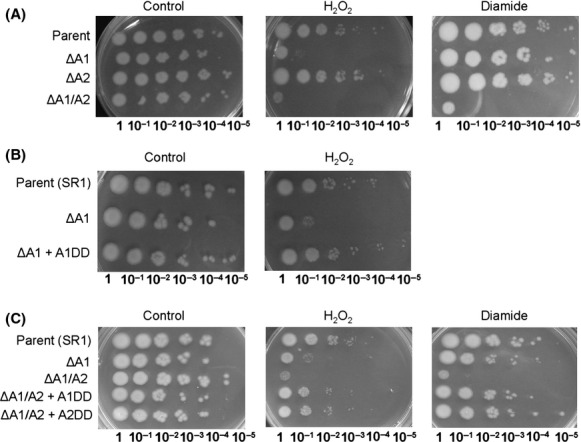
Spx mutants are sensitive to oxidative stress. Strains were grown on LB medium in the presence or absence of 100 μmol/L diamide or 0.44 mmol/L H_2_O_2_. Five microliter of the indicated dilutions were spotted onto LB agar (see Experimental Procedures). (A) Strains 7702 (Parent), ORB8170 (∆*spxA1*, ∆A1), ORB8438 (∆s*pxA2*, ∆A2), ORB8285 (∆*spxA1* ∆*spxA2*, ∆A1/A2). (B) 7702 Str^R^ (Parent [SR1]), ORB8398 (∆*spxA1* Str^R^, ∆A1), ORB8404 (∆*spxA1* Str^R^ ICE*Bs1*::*spxA1DD*, ∆A1 +A1DD). (C) 7702 Str^R^ (Parent [SR1]), ORB8398 (∆*spxA1* Str^R^, ∆A1), ORB8481 (∆*spxA1* ∆*spxA2* Str^R^, ∆A1/A2), ORB8485 (∆*spxA1* ∆*spxA2* Str^R^ ICE*Bs1*::*spxA1DD*, ∆A1/A2 +A1DD), ORB8486 (∆*spxA1* ∆*spxA2* Str^R^ ICE*Bs1*::*spxA2DD*, ∆A1/A2 +A2DD). Str^R^, streptomycin resistance.

*Bacillus anthracis* Sterne 7702, and derivatives ORB8170 (Δ*spxA1*), ORB8438 (Δ*spxA2*), and ORB8285 (Δ*spxA1* Δ*spxA2*), were grown in 40–60 mL of LB liquid medium lacking antibiotic at 37°C with agitation (200 rpm) and culture samples were taken at various time points during vegetative growth (see Fig. 2B). Cell pellets were harvested by centrifugation (5180*g*, 4°C, 10 min.) and stored until use at −80°C. Cell lysate was prepared by suspending the cell pellet in 200 μL–1 mL phosphate-buffered saline and vortexing with 0.1 mm glass beads for a total of 10 min, resting on ice every 5 min. After brief centrifugation to pellet the beads, the supernatant was analyzed for protein amount (Coomassie protein assay, Pierce) and an equal amount (25 or 30 μg) of cell lysate was separated on a SDS-polyacrylamide gel (15%) and transferred to nitrocellulose membranes. Immunoblotting was carried out in Tris-buffered saline plus 0.05% (v/v) Tween-20 using preabsorbed anti-Spx derived from *B. subtilis* purified Spx protein (Nakano et al. 2005) and alkaline phosphatase-anti-rabbit conjugated secondary antibody (Sigma) at appropriate dilutions. A representative immunoblot is shown for each experiment in Figures 1, 2, with each experiment done twice. Densitometry obtained with ImageJ (Schneider et al. 2012).

### Phenotype testing

A fresh colony grown on LB agar was used to inoculate an overnight LB culture (37°C, 200 rpm). Ten microliter of the overnight cultures were subcultured into 2 mL LB and grown (37°C, 200 rpm) until OD_600_ reached around 1.0. Five microliter of 10-fold serial dilutions (in T-base) of the cultures were spotted onto LB agar unsupplemented or supplemented with either 100 μmol/L diamide, 1.5 × 10^−3^% (0.44 mmol/L) hydrogen peroxide, or 0.001% deoxycholate. Sensitivity was checked after overnight incubation (37°C) of the agar plates.

### Microscopy

Two microliter aliquots of each spore culture grown for 18 h in SM broth were added to a precleaned glass slide, a coverslip was added, and cells were subsequently examined using a Leica DMIL Inverted Contrasting Microscope (Leica, Buffalo Grove, IL) equipped with a Leica HI Plan I 40× (numerical aperture, 0.5). Images were captured using a Leica DFC295 digital color camera and processed with Leica Software Application Suite V3.8.0.

## Results and Discussion

### Phenotypic analyses

#### Construction of Spx protease resistant and Δ*spx* mutants

Low levels of Spx are normally maintained in *B. subtilis* in part through posttranslational control involving degradation catalyzed by ClpXP and mediated by an adaptor protein, YjbH (Larsson et al. 2007; Garg et al. 2009). This mechanism can be averted in *B. subtilis* by substituting the last two C-terminal residues of SpxA to DD (see sequence alignment Fig. 1A, [Nakano et al. 2003]). We utilized the same method in *B. anthracis*. The SpxDD-producing strains used in this study were constructed by interspecies conjugation using a recombinant form of the integrative conjugative element ICE*Bs1* of *B. subtilis* ([Auchtung et al. 2005], Experimental Procedures) and a streptomycin-resistant variant of *B. anthracis* 7702 as recipient (7702 SR1). Mutation of SpxA1 and SpxA2 in this manner (SpxA1DD and SpxA2DD) caused accumulation of either protein compared to levels observed in the parental strain (see below in the case of SpxA1). In-frame deletions of both *spx* genes, Δ*spxA1* and Δ*spxA2*, were also constructed in order to study the phenotypic effects of these two paralogs in *B. anthracis*. These mutations were constructed using a markerless allelic replacement method developed by Janes and Stibitz ([Janes and Stibitz 2006], Experimental Procedures).

#### *spxA1* is essential for peroxide resistance; both *spxA1* and *spxA2* participate in disulfide stress tolerance

In *B. subtilis*, a Δ*spxA* mutant is sensitive to oxidant stress (Nakano et al. 2003). Therefore, experiments were conducted to determine if a similar phenotype was conferred by *B. anthracis spx* null mutations. Δ*spxA1* (ORB8170), Δ*spxA2* (ORB8438), and Δ*spxA1* Δ*spxA2* (ORB8285) mutant cells were treated with several oxidants that had been shown to trigger a stress response in related bacteria ([Kristoffersen et al. 2007; Rukmana et al. 2009; McLean et al. 2010; Antelmann and Helmann 2011; Tu et al. 2012b]; Experimental Procedures). Either SpxA1 or SpxA2 can function in disulfide-stress tolerance, as only th*e* Δ*spxA1* Δ*spxA2* (ΔA1/A2 in Fig. 2A and C) double mutant shows growth impairment upon diamide treatment. However, SpxA1 is essential for peroxide resistance, as Δ*spxA1* (ΔA1 in Fig. 2A, B, and C) showed significant peroxide sensitivity. Defects in diamide and peroxide resistance could be corrected by introducing eithe*r spxA1DD* o*r spxA2DD* constructs in *trans* (+A1DD or +A2DD in Fig. 2C), suggesting that either Spx protein can potentially function in peroxide-induced stress.

Bile salt can induce oxidative stress and disulfide formation in bacteria (Rodriguez-Beltran et al. 2012; Yang et al. 2013). Δ*spxA1* and Δ*spxA1* Δ*spxA2*, but not Δ*spxA2*, mutants were sensitive to the bile salt deoxycholate (Fig. S1).

#### SpxA1 and SpxA2 differ in transcriptional regulation during disulfide stress

Experiments were conducted to determine if the *spxA* paralogous genes were induced by disulfide stress, as part of the oxidative stress response in *B. anthracis*. cDNA was synthesized using RNA harvested from cells that were untreated or treated with 1 mmol/L diamide for 20 min and analyzed by RT-qPCR to determine the relative amount of *spxA1* or *spxA2* transcript produced. *spxA1* or *spxA2* transcript amounts were normalized to a portion of the *gatB*/Yqey (BA4533) transcript, which served as an endogenous control. The *gatB*/Yqey transcript is stable during the *B. anthracis* cell cycle (Reiter et al. 2011), and we observed no increase in this transcript in response to elevated SpxA1 or SpxA2 levels (Table 2); however, in the presence of diamide the *gatB*/Yqey transcript increased more than threefold (1.65 *C*_T_). We also found a similar change using the16S gene transcript when used as an endogenous control (data not shown). Although fold-changes are not absolutely comparable in this experiment because of the severe stress applied to the cell by diamide treatment, we believe that general trends in transcript amount could be discerned for both *spx* paralogs. The level of *spxA2* transcript increased 150- to 350-fold after diamide treatment; conversely, the *spxA1* transcript amount remained nearly unchanged or decreased slightly (data not shown), suggesting that the observed repression of *spxA1* exerted by SpxA2 that was uncovered from transcriptomic analysis (see below) was ongoing during disulfide stress.

**Table:** Comparison of select transcript fold-changes by microarray and RT-qPCR

Gene tag	Description	Microarray results^1^				RT-qPCR validation^2^					
		SpxA1DD	SpxA2DD	SpxA1DD	SpxA2DD
BA4533	*gatB/*Yqey	No change under conditions studied				–					
BA1200	*spxA1*	3.8	−3.5	Not determined	−4.4
BA1118	*yvrG*, sensor histidine kinase of YvrHG two-component system involved in peptidoglycan biosynthesis	17.9	No change^3^	2.9	1.4
BA1263	CoADR homolog	12.9	5.9	4.2	3.4
BA0774	CoADR-RHD (rhodanese homology domain)	2.3	12.8	3.7	7.9
BA1951	Putative oxidoreductase, conserved only in *B. cereus*/*thuringiensis* group	32.6	2.9	5.1	3.5
BA0847	*racE-1,* Glu racemase	No change^3^	15.9	4.6	7.3
BA1208	*yjbH,* adaptor protein for Spx_*Bsu*_ proteolysis	8.1	6.9	10.7	6.4
BA5387	*trxB,* thioredoxin reductase	3.1	4.3	4.1	4.0

1 Averaged fold-change of three biological replicates (see Experimental Procedures).

2 Averaged fold-change of two biological replicates, one biological replicate used RNA extracted for microarray experiments and one replicate independently isolated under similar growth conditions (see Experimental Procedures).

3 No change is a fold-change <2.

**Figure 3:**
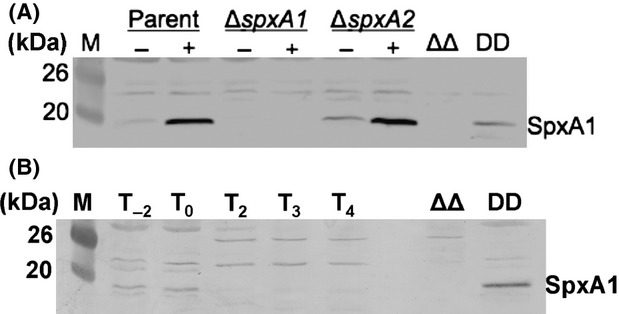
Protein levels of SpxA1 measured by Western blot. (A) SpxA1 levels in cultures grown with and without 1 mmol/L diamide (25 μg total protein applied; ΔΔ, Δ*spxA1* Δ*spxA2* (ORB8285); DD, P_spank(hy)_-*spxA1*DD (ORB7863) +IPTG 45 min.). (B) Expression profile of SpxA1 during the *B. anthracis* vegetative life cycle (30 μg total protein loaded; Lanes: T_−2_ through T_4_, hours during vegetative growth; T_0_, transition to stationary phase; ΔΔ, Δ*spxA1* Δ*spxA2* [ORB8285]; DD, P_spank(hy)_-*spxA1*DD [ORB7863] +IPTG 45 min.). Representative gels are shown in (A) or (B), each experiment was done twice. Growth conditions for obtaining cells for whole cell extracts are presented in Experimental Procedures.

Although *spxA1* transcription was not stimulated by diamide treatment, the protein concentration might increase if a posttranslational control mechanism similar to that governing Spx in *B. subtilis* is operating in *B. anthracis*. The presence of both *yjbH* and *clpXP* orthologs in *B. anthracis* suggests that SpxA1 and SpxA2 might be subject to a similar mechanism of proteolytic control (Larsson et al. 2007; Garg et al. 2009). Therefore, the amount of SpxA1 protein after diamide-induced stress in *B. anthracis* was examined. The culture extracts of the *B. anthracis* parent (7702), Δ*spxA1* (ORB8170), and Δ*spxA2* (ORB8438) grown in LB medium were used in Western blotting experiments with preabsorbed rabbit polyclonal anti-serum raised against *B. subtilis* SpxA, which cross-reacts only with SpxA1. Although very low levels of SpxA1 protein were observed in the parent strain grown without stress, the amount of SpxA1 increased at 20 min after the addition of 1 mmol/L diamide (57-fold), suggesting that posttranslational, rather than transcriptional, control of *spxA1* governs SpxA1 concentration in response to oxidative stress (Fig. 3A). Interestingly, SpxA1 protein levels also increased in the Δ*spxA2* (ORB8438) mutant without stress (7.4-fold), but remained unchanged in the Δ*spxA2* mutant after diamide treatment (1.2-fold), suggesting that SpxA2-dependent dampening of *spxA1* expression likely occurs at the transcriptional level (Fig. 3A).

One likely scenario for the increase in *B. anthracis* SpxA1 and SpxA2 concentration may be during germination and outgrowth, where newly formed vegetative cells would presumably encounter higher levels of ROS. This regulation could extend to spore germination and outgrowth in the oxidizing environment of the lysosome within the host macrophage. In line with this argument, previous transcriptomic studies identified increased *spxA1* transcript during spore outgrowth, increased *spxA2* transcript during sporulation, and both paralogous transcripts increased inside murine macrophage cells (Bergman et al. 2006, 2007). In keeping with the previous transcriptomic results, we found that SpxA1 was produced during exponential growth and that its concentration declined during early stationary phase (Fig. 3B).

The spike in *spxA2* transcriptional activity upon diamide treatment does not correlate well with the contribution of its product toward diamide resistance, as this function is served primarily by SpxA1 based on sensitivity tests (Fig. 2). There are other factors, aside from transcriptional control that can figure prominently in the final level of SpxA2 activity, notably protein stability and redox activation of the Spx protein through its redox disulfide center. How these features of Spx control might differ between the two paralogs is currently not known. Differences in affinity for RNA polymerase binding surface that engages Spx protein, or holoenzyme preference exhibited by the two paralogs might also account for the disparate contributions of SpxA1 and A2 to oxidant resistance. Evidence from studies of *B. subtilis* suggest that the *spx* paralog, *mgsR* is transcriptionally activated by the σ^B^ form of RNA polymerase, after which the MgsR protein associates with σ^B^ holoenzyme (Reder et al. 2012). However, the finding that each of the *B. anthracis spx* paralogous genes can complement a Δ*spxA* mutation in *B. subtilis* suggests that each product can engage the σ^A^ form of RNA polymerase (data not shown).

Together our data suggest that both SpxA1 and *spxA2* are upregulated when grown in the presence of the oxidant diamide; but different mechanisms exist for maintaining the level of each paralog in *B. anthracis*. SpxA1 is likely governed posttranslationally by a mechanism similar to that in *B. subtilis;* whereas another unknown control mechanism responsive to oxidative stress functions in elevating *spxA2* transcript levels.

#### Increased SpxA1 affects sporulation efficiency in *B. anthracis*

The requirement for SpxA1 and SpxA2 in germination/outgrowth and sporulation was also examined (Data S1). The results showed that there was no difference in the sporulation, germination, and outgrowth efficiency between the Δ*spx* mutants and the parent strain (7702 SR1), indicating that SpxA1 or SpxA2 are not required for the development or outgrowth from the spore state in *B. anthracis* (data not shown).

**Figure 4:**
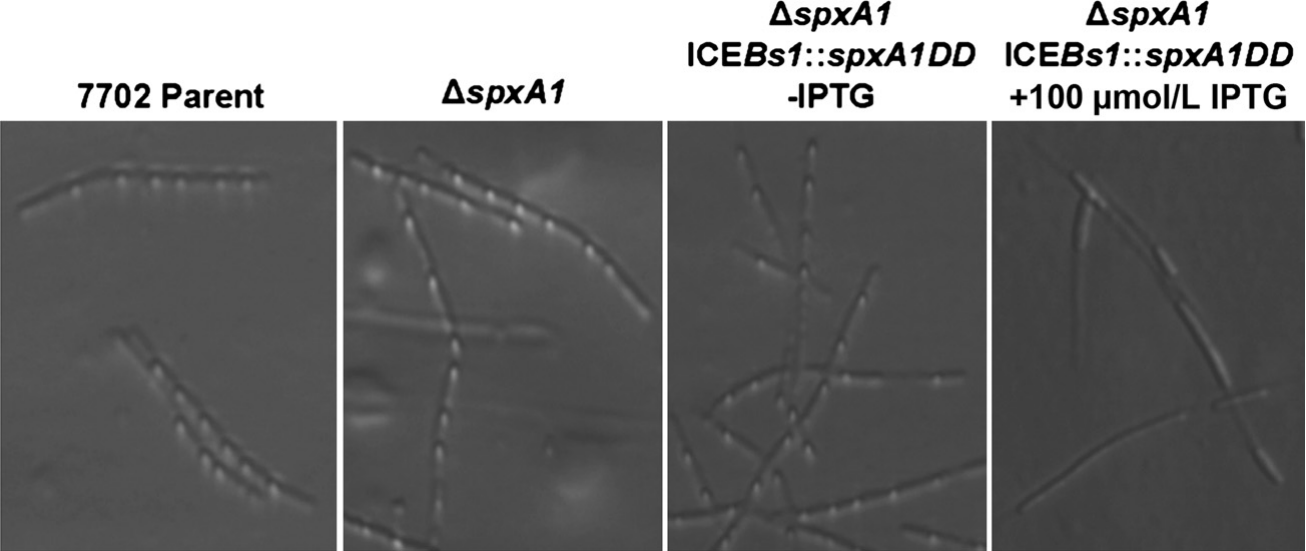
Micrographs of endospore-forming cells containing varying levels of SpxA1 protein after 24 h of growth in SM at 30°C. The phase-bright endospores are readily visualized in the *Bacillus anthracis* Sterne 7702 Parent and Δ*spxA1* mutant (1st and 2nd Panels). In the presence of 100 μmol/L IPTG, the Δ*spxA1* ICE*Bs1*::P_spank(hy)_-*spxA1DD* mutant (4th Panel) produced somewhat phase-bright bodies/filaments instead of forming endospores, whereas this strain readily produced endospores similar to the 7702 Parent strain in the absence of IPTG (3rd Panel).

Similar to overexpressing Spx in *B. subtilis* (Nakano et al. 2001), overexpression of SpxA1DD, but not SpxA2DD, inhibited sporulation to a large extent. Cells containing ICE*Bs1*::P_spank(hy)_-*spxA1DD* (ORB8404) grown in SM the presence of 100 μmol/L IPTG exhibited aberrant cell morphology, compared with the single Δ*spxA1* (ORB8170) or parental 7702 strains, at 18 h after inoculation with no visible endospores (Fig. 4), although some colony-forming units (CFUs) were obtained after heat treatment and plating onto LB-agar plates (postheat plated CFU/mL: ORB8404 −IPTG = 2.9 × 10^7^, +IPTG = 2.0 × 10^6^; compared with ORB8170 [7702 Δ*spxA1*] = 4.4 × 10^7^ and Parent 7702 = 4.9 × 10^7^). At 42 h after inoculation, the aberrant cells had completely lysed, no phase bright spores could be detected by microscopy, and plated CFUs resulted in (5% of the CFUs after heat treatment obtained on minus IPTG culture plates (preheat plated CFU/mL: ORB8404 −IPTG = 1.62 × 10^8^, +IPTG = 3.8 × 10^6^; postheat plated CFU/mL: ORB8404 −IPTG = 1.62 × 10^8^, +IPTG = 2.8 × 10^6^). These results suggest a role for activated Spx in delaying complex developmental process during periods of oxidative stress, a role previously proposed for *B. subtilis* SpxA (Nakano et al. 2003).

### Transcriptomic analyses

#### Transcriptomic changes in the presence of protease-resistant SpxA1DD or SpxA2DD in *B. anthracis*

**Figure 5:**
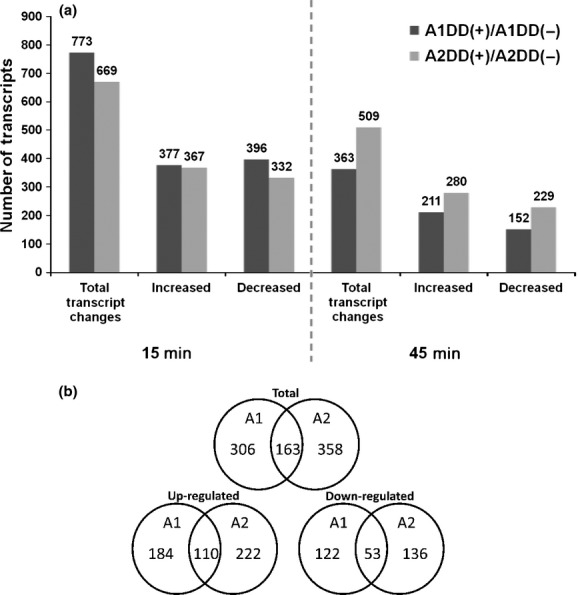
Transcriptomic changes in the presence of SpxA paralogs in *Bacillus anthracis*. (A) The total number of transcripts that change after induction of SpxA1DD (dark gray bars: A1DD[+]/A1D[−]) or SpxA2DD (light gray bars: A2DD[+]/A2DD[−]) for either 15 or 45 min. Total number of transcripts in each category listed above each bar. (B) Venn diagrams representing the total common transcript changes (≥twofold) observed when either SpxA1DD or SpxA2DD is induced for 45 min. Genes that overlap between the 15- and 45-min time points are considered common genes. A list of up- or downregulated transcripts, grouped by cellular function, can be found in Tables S1, S2. Transcripts were grouped by cellular function based on annotations from Genolist (http://genolist.pasteur.fr) and primary literature sources (Moszer et al. 2002; Pohl et al. 2011). Growth conditions for obtaining total cellular RNA and subsequent microarray experiments are presented in Experimental Procedures.

To identify genes potentially regulated by SpxA1 or SpxA2, we compared gene expression profiles between the parent 7702 strain and its isogenic derivatives expressing protease-resistant forms of either Spx paralog (parent vs. ICE*Bs1*::P_spank(hy)_-*spxA1DD* or parent vs. ICE*Bs1*::P_spank(hy)_-*spxA2DD*) using microarray hybridization array analysis. The strains were grown in LB broth and induced by the addition of IPTG for 15 or 45 min prior to culture sampling. Figures 5A and B summarize the overall transcriptional trends from the microarray experiments, and lists of all transcript changes can be found in Tables S1, S2.

**Figure 6:**
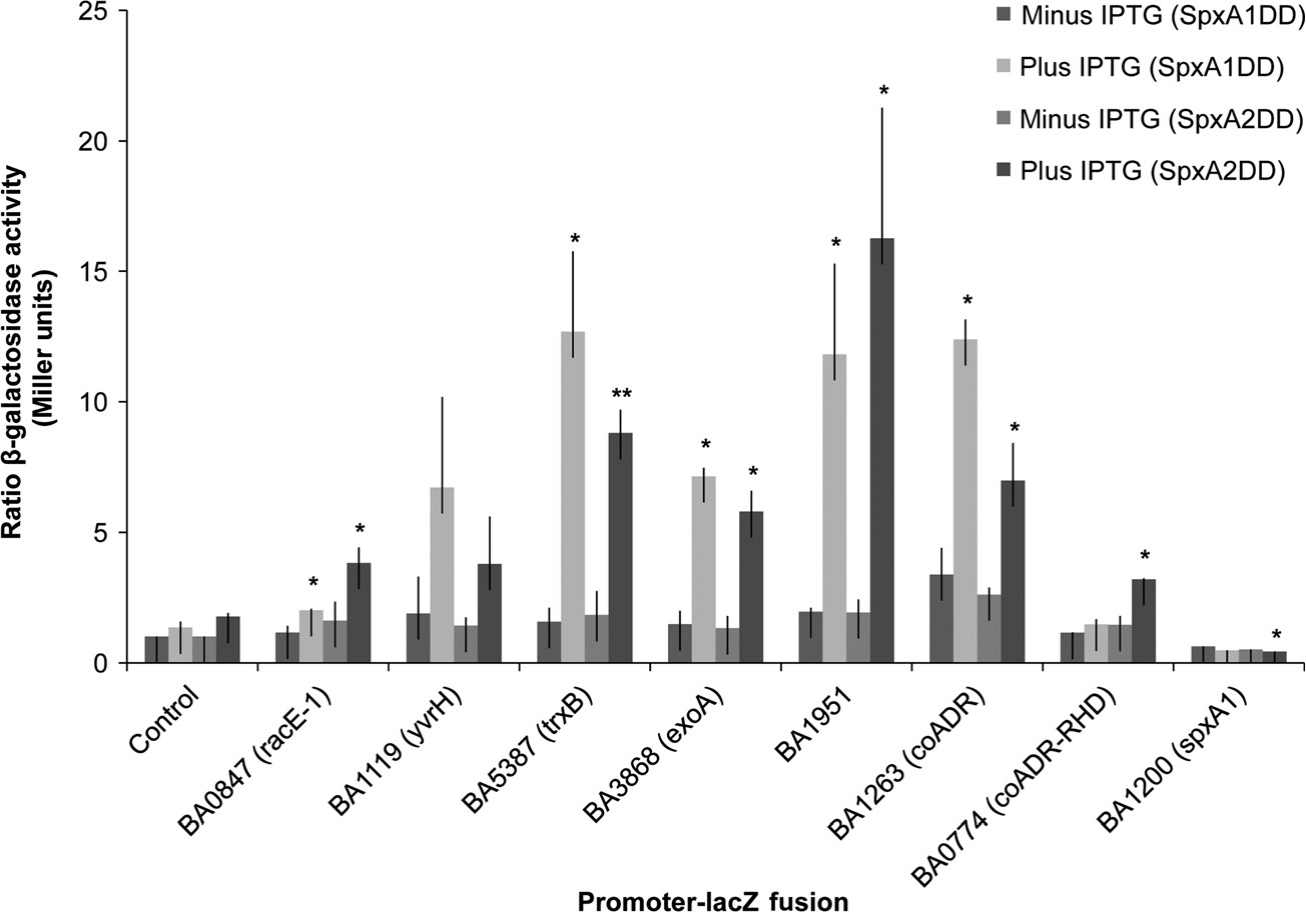
SpxA1DD and SpxA2DD activate several *Bacillus anthracis* genes in *B. subtilis*. Promoters of selected genes (horizontal axis) controlled by either SpxA1DD or SpxA2DD, as identified by microarray, were transcriptionally fused to a promoterless *lacZ* and integrated into the chromosome at the *thrC* locus of a *B. subtilis* JH642 Δ*spx* strain harboring an IPTG-inducible copy of either *spxA1DD* or *spxA2DD* integrated at the *amyE* locus. β-galactos*idase activi*ty was measured in these strains at 30 min. intervals after the addition of 1 mmol/L IPTG. The maximal activity, which was usually observed at 1–2 h after IPTG induction, was divided by the β-galactosidase activity when IPTG was added; except for P_*spxA1*_*-lacZ*, where the minimal activity was divided by the β-galactosidase activity when IPTG was added (SpxA1DD: minus IPTG 0.64 ± 0.16, plus IPTG 0.49 ± 0.2; Sp*xA2*DD*:* minus IPTG 0.52 ± 0.27, plus IPTG 0.44 ± 0.18). The ratio was shown as the average of three biological triplicates with standard deviation. Significance was determined by a two-tailed *T*-test comparing plus IPTG to minus IPTG ratios; *P < 0.05 and **P < 0.005 (SpxA1DD: Control [empty vector] *P* = 0.272, BA0847 [*racE-1*] *P = 0*.026*,* BA1119 [*yvrH*] *P* = 0.055, BA5387 [*trxB*] *P* = 0.020, BA3868 [*exoA*] *P* = 0.0057, BA1951 *P* = 0.036, BA1263 [*coADR*] *P* = 0.005, BA0774 [*coADR-RHD*] *P* = 0.108, BA1200 [*spxA1*] *P* = 0.463; SpxA2DD: Control [empty vector] *P* = 0.078, BA0847 [*racE-1*] *P* = 0.013, BA1119 [*yvrH*] *P* = 0.179, BA5387 [*trxB*] *P* = 0.001, BA3868 [*exoA*] *P* = 0.015, BA1951 *P* = 0.032*,* BA1263 [*coADR*] *P* = 0.027, BA0774 [*coADR-RHD*] *P* = 0.009, *spxA1 P* = 0.020).

The expression trends from the microarray transcriptomic analysis underwent validation by (RT-qPCR, Table 2) and by assaying β-galactosidase activity in *B. subtilis* strains harboring promoter*–lacZ* transcriptional fusions (Fig. 6). For RT-qPCR, the transcripts were normalized against a portion of the *gatB*/Yqey (BA4533) transcript (Reiter et al. 2011). The individual transcript values were reported as the averaged fold-change for two biological replicates (Table 2). β-galactosidase assays were performed by constructing *lacZ* transcriptional fusions to several gene promoters (intergenic regions roughly 200–600 nucleotides upstream of the start codon, extending downstream into the 5′ end of the coding sequence), and integrating these alleles into the chromosome of *B. subtilis* strains harboring a deletion of the native *spxA* gene as well as IPTG-inducible versions of *B. anthracis spxA1DD* or *spxA2DD*. β-galactosidase activity in the presence or absence of IPTG was then measured in these strains and the ratio of β-galactosidase activity (highest activity/starting time point activity) was plotted for each strain when grown in the presence or absence of IPTG (Fig. 6, see Experimental Procedures).

There were 773 and 699 transcript changes (≥twofold), respectively, when either *spxA1DD* or *spxA2DD* was expressed for 15 min. After 15 min of induction, transcript levels of 377 genes were higher and those of 396 genes were lower in the SpxA1DD-producing strain; and transcript levels of 367 genes were higher and 332 genes were lower when SpxA2DD production was induced (Fig. 5A). After 45 min of induction, only 363 transcript changes (≥twofold; 211 up- and 152 downregulated) were noted in SpxA1DD with 306 of those transcripts also found at higher levels after 15 min of induction (Fig. 5A and B). Far more transcripts changed after prolonged SpxA2DD induction (45 min) with 509 total transcript changes (≥twofold; 280 up- and 229 downregulated), while 358 of these also exhibited a change in expression after 15 min of induction (Fig. 5A and B). The total number of upregulated genes between SpxA1DD and SpxA2DD after 45 min of induction was 163, with 110 upregulated and 53 downregulated (Fig. 5B). A list of all transcriptomic changes can be found in Tables S1, S2. Due to the large number of transcripts that showed changes in abundance when either SpxA1DD or SpxA2DD was present, we chose to focus on upregulated transcripts after 15 min of SpxA1DD or SpxA2DD induction for further analysis. Lists of all transcript changes ≥twofold can be found in Tables S1, S2; and lists of genes whose transcripts changed by ≥threefold, grouped by function, can be found in Tables S3, S4.

Many genes having elevated transcript amounts were uncovered in analysis of *spxA1DD* and *spxA2DD* transcriptomes, suggesting some overlap in the composition of SpxA1 and SpxA2 regulons. In total, several upregulated genes unique to either SpxA1 or SpxA2 regulons, 184 and 222, respectively, were also identified (Fig. 5B), supporting the hypothesis that each Spx protein has some unique sequence recognition properties (Tables S1, S2) and/or perhaps holoenzyme specificity.

### Redox homeostasis

In Bacilli, as in many bacteria, low-molecular-weight thiols are important for maintaining redox homeostasis and alleviating stress caused by toxic oxidants (Newton et al. 1996, 2009). As regulated by orthologous *spx* in *B. subtilis*,* trxA*,* trxB*, and thioredoxin family protein, *ytpP*, among others, were also upregulated in the *spxA1DD* and *spxA2DD B. anthracis* strains (Nakano et al. 2003; Zuber et al. 2011; Rochat et al. 2012). RT-qPCR and β-galactosidase assays further confirmed a role for both paralogs in the transcriptional control of *trxB* (Table 2 and Fig. 6).

In several organisms that do not produce glutathione (GSH), coenzyme A (CoASH) can become an important molecule involved in intracellular redox functions (Delcardayre and Davies 1998; Delcardayre et al. 1998). In the presence of SpxA1DD or SpxA2DD, the transcripts specifying two CoASH disulfide reductases, BA1263 (CoADR) and BA0774, which encodes an isoform of CoADR that bears a rhodanese domain (CoADR-RHD), increased >threefold, suggesting that both genes are under the control of the *spx* paralogs in *B. anthracis* (Table 2). By RT-qPCR, we observed an increase in BA1263 and BA0774 transcripts with either SpxA1DD or SpxA2DD induction (Table 2); however, in β-galactosidase assays only SpxA2 could activate P_BA0774_-lacZ, while either paralog activated P_BA1263_-lacZ (Fig. 6). It may be possible that an activator (missing in *B. subtilis*) is necessary, in conjunction with SpxA1, to stimulate BA0774 transcription. *B. subtilis* lacks CoADR and CoADR-RHD isoforms, perhaps utilizing bacillithiol and cysteine instead of CoA in the cytoplasmic redox buffer (Helmann 2011).

The synthesis of bacillithiol has been linked to diamide stress and the *spx* regulon (Gaballa et al. 2010). Similarly, we found several orthologous genes encoding proteins functioning in the sequential synthesis of UDP-GlcNAc to bacillithiol that are upregulated upon induction of SpxA1DD and SpxA2DD ([Gaballa et al. 2010]; Tables S1, S2). In particular, the transcript for the glycosyltransferase gene, *bshA*, increased 4.3-fold and 2.5-fold in the SpxA1DD- and SpxA2DD-producing strains, respectively (Tables S1, S2); and both orthologous genes encoding N-acetylhydrolases, *bshB1* and *yojG*, as well as the Cys-adding enzyme, *yllA*, were also upregulated >twofold in the presence of either SpxA1DD or SpxA2DD (Tables S1, S2), suggesting expression that is governed by SpxA1 and SpxA2 resulting in increased production of bacillithiol in *B. anthracis*. Additionally, an ortholog of a *B. subtilis* gene putatively involved in the reduction of oxidized bacillithiol, namely *ypdA*, encoding a pyridine nucleotide-dependent disulfide oxidoreductase, was upregulated >twofold by either SpxA1DD or SpxA2DD (Tables S1, S2). Two putative bacilliredoxin genes, *yphP* and *ytxJ,* also showed elevated transcript levels after SpxA1DD or SpxA2DD production. *ytxJ* transcript level was increased 2.5-fold by SpxA1DD and *yphP* 2.3-fold by SpxA2DD (Tables S1, S2).

Several SpxA1DD- and SpxA2DD-activated genes were also observed to be transcriptionally upregulated in peroxide-treated cells (Table S5; [Pohl et al. 2011]), and likely include genes required for peroxide-induced stress tolerance, including (*trxB*), DNA repair (*uvrC*), detoxification (nitroreductase, aldehyde dehydrogenase), and export (MATE efflux family protein).

*Bacillus anthracis* Spx proteins also stimulated the transcription of genes whose products function in overcoming DNA damage, which studies have shown is a direct consequence of oxidative stress brought about by encounters with oxidizing agents such as H_2_O_2_ (Imlay and Linn 1988; Imlay et al. 1988). We found that SpxA2DD upregulated BA3868, the ortholog of *B. subtilis exoA*, an apurinic-apyrimidinic endonuclease important in base excision repair (Ibarra et al. 2008) and genes encoding components of the ABC excinuclease (*uvrC* and *uvrD*) (Tables S1, S2) are also upregulated by SpxA1DD and SpxA2DD. Both Spx paralogs were able to activate a P_BA3868_-*lacZ* reporter construct in *B. subtilis*, suggesting that BA3868 is a member of SpxA1 and SpxA2 regulons (Fig. 6). ExoA_*Bsu*_ is an important component of spore outgrowth and germination when oxidative DNA damage occurs, presumably when the once dormant spore is suddenly exposed to oxygen upon germinated spore hydration (Ibarra et al. 2008). *Bacillus subtilis* cells deficient in *exoA* were significantly delayed in spore outgrowth and increasingly sensitive to H_2_O_2_ (Ibarra et al. 2008).

Several genes encoding hypothetical products, many having no homology outside of the *B. cereus/B. thuringiensis* group, were also upregulated when SpxA1DD or SpxA2DD were produced (Tables S1, S2). We focused on one of these genes, a putative oxidoreductase (BA1951), which in the microarray analysis was induced 32.6-fold in cells expressing SpxA1DD. Cells expressing SpxA2DD had a smaller increase in BA1951 transcript (2.9-fold) (Table 2). The microarray trends were mirrored in β-galactosidase (activation of P_BA1951_*-lacZ* by SpxA1DD [*P* = 0.036] or SpxA2DD [*P* = 0.032]) and RT-qPCR (5.1-fold, SpxA1DD, 3.5-fold SpxA2DD) assays, although the transcript changes determined by RT-qPCR were lower than transcript changes seen by microarray (Table 2).

Several genes functioning in NAD(P)H-dependent interconversions were also highly upregulated by SpxA1 and SpxA2. These include genes encoding putative alcohol, aldehyde, and quinone dehydrogenases (BA0838, BA2647, and BA3438) for which orthologs exist in *B. subtilis* (YogA and YdeQ). Interestingly, the *B. subtilis* orthologous genes are not members of the Spx regulon, suggesting that the SpxA1 and SpxA2 regulons of *B. anthracis* include members with different metabolic capabilities than those belonging to the *B. subtilis* Spx regulon. The products of the Spx-activated genes could function in detoxification or generation of reduced NAD(P)H for coping with oxidative stress (Rochat et al. 2012). Taken together these results reinforce the view that SpxA1 and SpxA2 act as transcriptional regulators to activate genes involved in the reduction of oxidized thiols in *B. anthracis*.

### SpxA2 repression of *spxA1*

Increasing the amount of SpxA2DD led to a 3.5-fold decrease in the *spxA1* transcript, suggesting a relationship between SpxA2 and the transcriptional control of *spxA1* expression (Table 2). By microarray, the induction of *spxA2* was not dependent on SpxA1DD, suggesting that another regulatory mechanism functions in elevating *spxA2* transcript levels during oxidative stress.

The *spxA1* repression by SpxA2DD was confirmed in three ways: (1) a roughly fourfold decrease in transcript amount was found by RT-qPCR when SpxA2DD was present (Table 2); (2) a roughly twofold increase in *spxA1* transcript was detected in a Δ*spxA2* background (data not shown); and (3) expression of a P_*spxA1*_-*lacZ* reporter fusion was repressed when SpxA2DD was produced in *B. subtilis* (Fig. 6). These transcriptomic results parallel the expression results reported above, in which an increase in the SpxA1 protein amount was detected in the Δ*spxA2* mutant (Fig. 3A). In conjunction with the large increase in the *spxA2* transcript in the presence of diamide stress, it may be that the muted transcriptional response of *spxA1* in *B. anthracis* Sterne 7702 is a consequence of SpxA2-dependent repression, which seems to persist even in the presence of disulfide stress. However, as shown by Western blot experiments, the SpxA1 protein amount increased during diamide stress (Fig. 3), suggesting that the oxidative stress response in *B. anthracis* includes a posttranscriptional mechanism that oversees SpxA1 accumulation instead of a surge in transcriptional activity that characterizes *spxA2* induction. Together, these experiments indicate that SpxA2 negatively affects *spxA1* expression.

### Cell wall biosynthesis and sporulation

The involvement of SpxA orthologs in control of genes that function in cell wall metabolism has been observed in other Gram-positive bacteria (Prudhomme et al. 2006; Veiga et al. 2007; Suntharalingam et al. 2009; Turlan et al. 2009; Eldholm et al. 2010; Kajfasz et al. 2010). The microarray data suggest that both *B. anthracis* paralogs can increase expression of genes involved in cell wall or membrane synthesis, remodeling, and maintenance (Tables S1, S2).

Two two-component signal transduction systems specified by the *yycFG* and BA1119-BA1118 (*yvrHG*) operons were upregulated in response to increased amounts of SpxA1DD, but not SpxA2DD. Transcription of *yycFG* increased more than twofold (2.2-fold *yycF*, 3.5-fold *yycG*), while *yvrGH*, showed an even greater transcript increase (17.9-fold *yvrG*, 12.9-fold *yvrH*). Upon induction of SpxA1DD, P_BA1119_-*lacZ*, encompassing the promoter for the gene encoding the YvrH DNA-binding protein, was activated to nearly significant levels (*P* = 0.055; Fig. 6). Similarly in RT-qPCR experiments, SpxA1DD increased the BA1118 (*yvrG*) transcript (2.9-fold), although to lesser amounts than found by microarray. Neither BA1119 promoter activation (Fig. 6) or BA1118 transcript increase (1.4-fold, Table 2) were detected after SpxA2DD induction, suggesting that the *yvrHG* operon is regulated by SpxA1DD only. Both YycFG and YvrGH are responsible for peptidoglycan biosynthesis and cell wall homeostasis in *B. subtilis* (Serizawa et al. 2005; Szurmant et al. 2007a). The essential YycFG system of *B. subtilis* also has two accessory proteins YycH and YycI, which are transcribed as part of the same operon (Szurmant et al. 2005, 2007b) and both *yycHI* orthologous transcripts in *B. anthracis* were elevated only when SpxA1DD was produced (3.3-fold *yycH*, 2.1-fold *yycI*).

*Bacillus anthracis* possesses two glutamate racemases, which catalyze the conversion of L-glu to D-glu for the synthesis of poly-γ-glutamate capsule and peptidoglycan (Dodd et al. 2007). One of these glutamate racemase-encoding genes, BA0847 (*racE-1*)*,* was upregulated in the SpxA2DD-producin*g* strain almost 16-fold by microarray analysis, while its expression did not change when SpxA1DD was overproduced. However, using RT-qPCR, we were able to detect an elevated BA0847 (*racE-1*) transcript concentration in the SpxA1DD-producing strain (4.6-fold) as well as the expected transcript increase (7.3-fold) by SpxA2DD (Table 2), suggesting that there may be an overestimation of genes uniquely controlled by only one paralogous Spx, as indicated by microarray. A P_BA0847_-*lacZ* transcriptional fusion was also activated in the presence of SpxA2DD or SpxA1DD (Fig. 6), confirming our RT-qPCR results.

Production of SpxA2DD also increased the level of transcripts encoded by three sporulation genes, *spoIIID, spoIIIE*, and *rsfA*, whose gene products are involved in sporulation-specific transcriptional regulation and DNA translocation (Kunkel et al. 1989; Bath et al. 2000; Wang et al. 2006). As the Δ*spxA2* mutant does not seem to have altered sporulation efficiency or outgrowth patterns, the increased transcript amount of these sporulation-specific genes after SpxA2DD induction may be an indirect effect. A more detailed study of *spxA2* is underway.

#### SpxA*-*activated gene expression during disulfide stress

**Figure 7:**
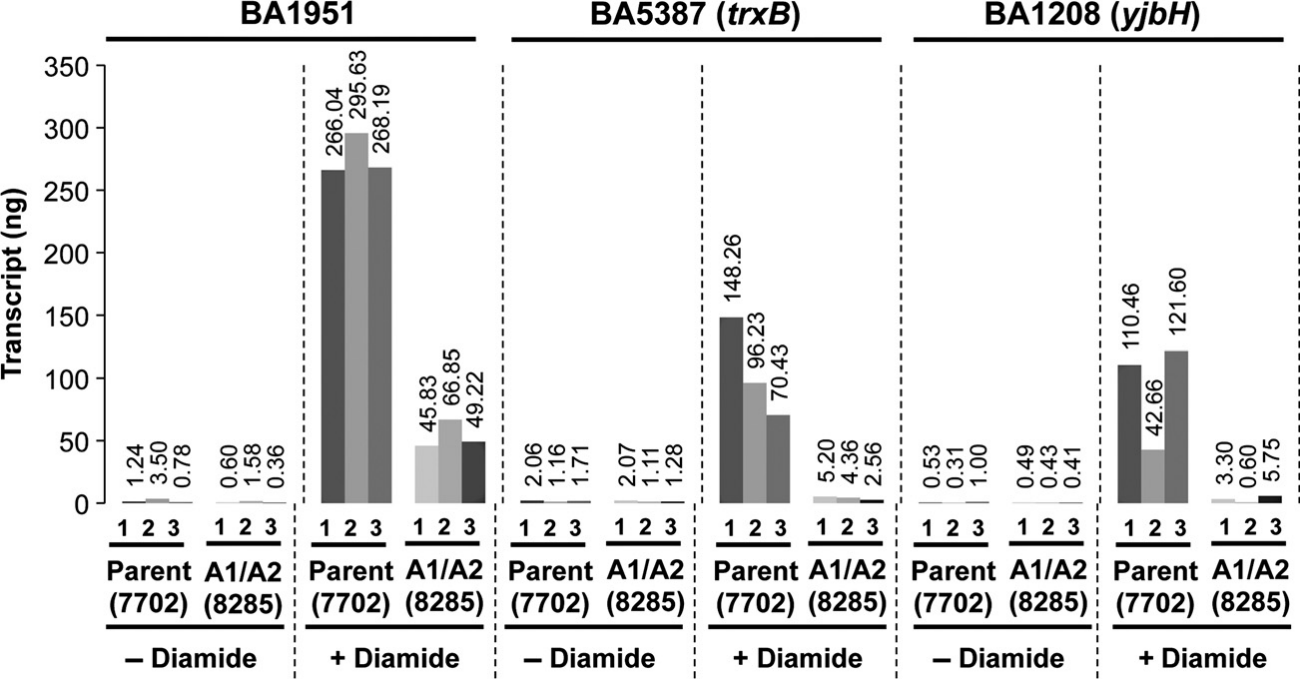
SpxA1 and SpxA2 activate genes that potentially function in thiol homeostasis. RT-qPCR of BA1951, BA5387, and BA1208 transcripts of the parent (*Bacillus anthracis* Sterne 7702) and Δ*spxA1* Δ*spxA2* mutant (A1/A2, ORB8285) from triplicate cultures with and without 1 mmol/L diamide for 20 min (see Experimental Procedures). Each biological replicate is graphed individually (denoted as 1, 2, or 3) to show the variation in the transcript amount (values listed above each bar).

We chose to further characterize two genes regulated by both Spx paralogs and putatively involved in thiol homeostasis, BA5387 (*trxB*) and BA1951 (a putative oxidoreductase), by measuring their transcript levels after diamide stress (1 mmol/L) for 20 min (Fig. 7; Experimental Procedures). The levels of the two transcripts were normalized to the *gatB*/Yqey (BA4533) transcript as described above. Without diamide treatment, *gatB*/Yqey transcript levels remained unchanged. However, in the presence of diamide the *gatB*/Yqey transcript increased more than threefold in the parent and roughly eightfold in the Δ*spxA1* Δ*spxA2* mutant. As BA1951 and BA5387 transcripts increased by at least 80-fold when diamide was applied, we believe that general comparisons between untreated and treated parent and Δ*spxA1* Δ*spxA2* transcripts could reasonably be made.

In the absence of oxidative stress, BA1951 and BA5387 transcript levels remained low, but in the presence of diamide, both transcripts increased dramatically in the parent (7702), suggesting a role for both genes in SpxA-dependent disulfide stress tolerance (Fig. 7). In the double *spxA* mutant, BA1951 transcript levels decreased, but not to untreated levels, signifying that BA1951 is also regulated by a Spx-independent mechanism during oxidative stress (Fig. 7). The level of BA5837 (*trxB*) transcript in the double *spxA* mutant was only slightly induced by diamide, indicating a requirement for SpxA1 and/or A2 in activating BA5387 transcription during diamide-induced stress (Fig. 7). Taken together, we believe that the *B. anthracis* (*cereus*/*thuringiensis*)-specific BA1951 gene product is an oxidoreductase, and along with BA5387, have potential roles in oxidative stress defense.

In microarray experiments, the transcript levels of the *B.subtilis yjbH* ortholog, BA1208, was elevated when either SpxA1DD or SpxA2DD was present (Table 2, Tables S1, S2), indicating a requirement for one or both Spx paralogs in the activation of *yjbH* transcription. RT-qPCR experiments confirmed SpxA1DD and SpxA2DD transcriptional control of BA1208, even during disulfide stress where the transcript amount of BA1208 increased in the parent (7702) but not in the Δ*spxA1* Δ*spxA2* mutant (Table 2 and Fig. 7). Elevating the concentrations of YjbH and ClpX through a Spx-dependent mechanism of control might ensure that the means of Spx elimination is in place for when thiol homeostasis is restored following stress. Recent evidence from chromatin immune precipitation and transcription analysis indicated that the *clpX* gene is under positive transcriptional control by SpxA in *B. subtilis* (Rochat et al. 2012). However, we were unable to detect upregulation of the *B. anthracis clpX* (BA4074) gene in SpxA1DD- or SpxA2DD-producing cells.

In summary, four lines of evidence indicate that SpxA proteins of *B. anthracis* participate in the control of the oxidative stress response: (1) The *spxA1* null mutant shows heightened sensitivity to H_2_O_2_ treatment, while the *spxA1/spxA2* double mutant shows a severe growth defect when cells are treated with diamide; (2) SpxA1 protein is elevated upon diamide-induced stress, in parallel with elevated SpxA-dependent transcriptional induction; (3) SpxA1 or SpxA2, in protease-resistant form, activate the transcription of many genes that have been implicated in disulfide stress resistance and thiol homeostasis; (4) Genes likely involved in thiol homeostasis are induced by diamide treatment through an SpxA-dependent mechanism. Similar to the role of *spx* in *B. subtilis*, the *B. anthracis spx* paralogs are likely to be important transcriptional regulators during disulfide stress. Each paralog may differentially regulate genes involved in other stress-alleviating processes. Additional experiments are underway to further examine the function and regulation of these two paralogous genes and their products.

## Supporting Information

**Figure S1.** Spx mutants sensitive to oxidative stress. Strains were grown in LB medium in the presence or absence of 0.01% deoxycholate. Five microliter of the indicated dilutions were spotted onto LB agar (see Experimental Procedures). 7702 Str^R^(Parent [SR1]), ORB8398 (Δ*spxA1* Str^R^, ΔA1), ORB8481 (Δ*spxA1* Δ*spxA2* Str^R^, ΔA1/A2), ORB8485 *(*Δ*spxA1* Δ*spxA2* Str^R^ ICE*Bs1*::*spxA1DD,* ΔA1/A2 +A1DD), ORB8486 (Δ*spxA1* Δ*spxA2* Str^R^ ICE*Bs1*::*spxA2DD,* ΔA1/A2 +A2DD). Str^R^, streptomycin resistance.

**Data S1.** Sporulation and germination assays.

**Table S1.** SpxA1DD-regulated genes

**Table S2.** SpxA2DD-regulated genes.

**Table S3.** SpxA1DD-activated genes. Fifteen minutes after induction. .threefold increase.

**Table S4.** SpxA2DD-activated. Fifteen minutes after induction. .threefold increase.

**Table S5. ***SpxA1-* and *SpxA2-* controlled genes shown to be induced by peroxide treatment (Pohl et al. 2011).

**Table S6.** Oligonuceotide primers.
