# Supplementary figures and images for: Transcriptomic and phenotypic analysis of paralogous spx gene function in Bacillus anthracis Sterne
Source: Microbiologyopen. 2013 Jul 22;2(4):695–714. doi: 10.1002/mbo3.109 (PMC3831629; doi:10.1002/mbo3.109)

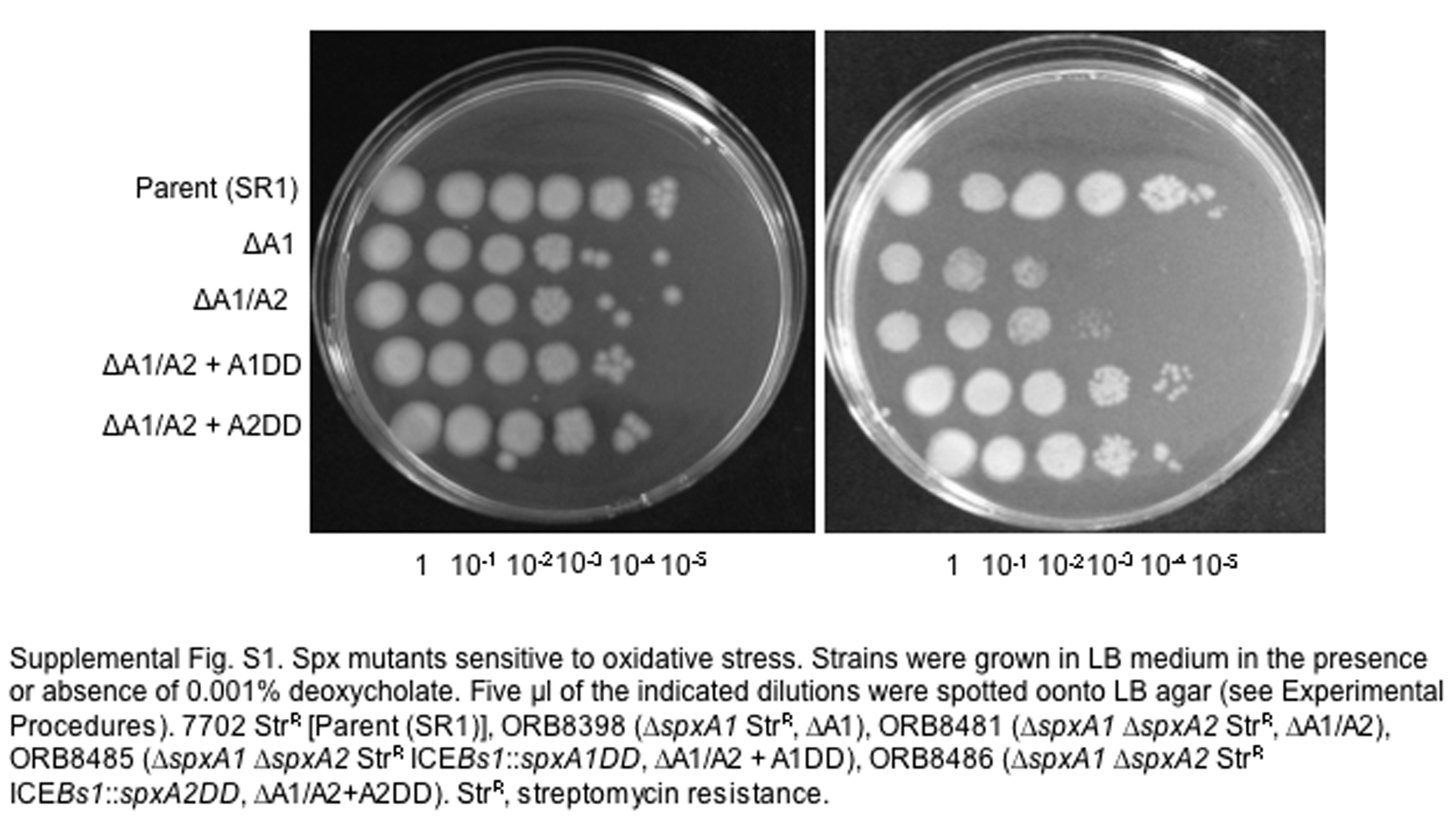

Supplement: Supplementary file 1 — Figure S1. Spx mutants sensitive to oxidative stress. Strains were grown in LB medium in the presence or absence of 0.01% deoxycholate. Five microliter of the indicated dilutions were spotted onto LB agar (see Experimental Procedures). 7702 StrR(Parent [SR1]), ORB8398 (ΔspxA1 StrR, ΔA1), ORB8481 (ΔspxA1 ΔspxA2 StrR, ΔA1/A2), ORB8485 (ΔspxA1 ΔspxA2 StrR ICEBs1::spxA1DD, ΔA1/A2 +A1DD), ORB8486 (ΔspxA1 ΔspxA2 StrR ICEBs1::spxA2DD, ΔA1/A2 +A2DD). StrR, streptomycin resistance. [file mbo30002-0695-SD1.tif]
