# Supplementary material for: Transcriptomic and phenotypic analysis of paralogous spx gene function in Bacillus anthracis Sterne
Source: Microbiologyopen. 2013 Jul 22;2(4):695–714. doi: 10.1002/mbo3.109 (PMC3831629; doi:10.1002/mbo3.109)
Supplement: Supplementary file 2 — Data S1. Sporulation and germination assays. [file mbo30002-0695-SD2.docx]

- **Isolation of Spores and Sporulation Assay.** Aliquots of glycerol stocks were used to inoculate pre-warmed sporulation medium (SM, XX) and grown until late log phase at 37°C, 200 rpm (preculture). The preculture was used to inoculate SM to a starting OD_600_ 0.01 and incubated at 30°C, 200 rpm. Spore development was monitored by phase-contrast microscopy and plating for CFU pre- and post-heating (65°C, 30 minutes) on brain-heart infusion (BHI) agar plates. Spore development in this medium was ≥95% by 24 hours and the assay was run for at least 48 hours after the initial inoculation. Over-expression of SpxA1DD or SpxA2DD was carried out by adding 100 µM IPTG to the growth medium of ORB8404 and ORB8405 cultures at the time of inoculation. In order to isolate large quantities of spores, 100-150 mL cultures were incubated at 30°C, 200 rpm for at least 72 hours before harvesting by centrifugation (5180 xg, 4°C, 10 min.), purification by renografin gradients ([Setlow, 1990](#_ENREF_78)), and storage at 4°C in sterile water protected from light.
- **Germination and Outgrowth Assay.** Aliquots of water-resuspended spore preparations were heat activated (70°C, 30 min.) and added to sterile germination buffer (50 mM Tris-HCl pH 7.5, 10 mM NaCl), lacking or containing 40 µM L-alanine and 250 µM Inosine, to a starting OD_580_ of 0.35-0.5. The progression of germination was carried out at room-temperature and monitored OD_580_ for 1 hr. For outgrowth experiments, germinating spores were added to pre-warmed LB liquid media after 20 min. of germination at a 1:10 dilution (37°C, 200 rpm) and monitored at OD_600_.
