# Supplementary material for: Transcriptomic and phenotypic analysis of paralogous spx gene function in Bacillus anthracis Sterne
Source: Microbiologyopen. 2013 Jul 22;2(4):695–714. doi: 10.1002/mbo3.109 (PMC3831629; doi:10.1002/mbo3.109)
Supplement: Supplementary file 3 — Table S1. SpxA1DD-regulated genes [file mbo30002-0695-SD3.docx]

Supplementary Table S1. SpxA1DD-regulated genes.

| SpxA1DD negatively regulated genes (after 15 minutes of SpxA1DD induction) | | | |
| --- | --- | --- | --- |
| Locus | Gene Description | Gene Annotation | Fold Change |
| BA3511 | membrane protein, putative | NA | -13.25 |
| BA5298 | nucleoside transporter, NupC family | NA | -11.59 |
| BA5072 | hypothetical protein | NA | -9.52 |
| BXA0061 | hypothetical protein, | NA | -8.62 |
| BA1430 | imidazoleglycerol phosphate synthase, cyclase subunit | hisF | -8.60 |
| BA3420 | conserved hypothetical protein | NA | -8.39 |
| BA2133 | molybdenum cofactor biosynthesis protein A | narA-1 | -7.81 |
| BA3147 | hypothetical protein | NA | -7.35 |
| BA1295 | immune inhibitor A metalloprotease | NA | -7.26 |
| BA1467 | flavohemoprotein | hmp | -7.03 |
| BA4130 | prophage LambdaBa02, repressor protein | NA | -6.92 |
| BA0981 | S-layer protein, putative | NA | -6.67 |
| BA1380 | transcriptional regulator, AsnC family | NA | -6.47 |
| BA3512 | membrane protein, putative | NA | -6.43 |
| BA1975 | DNA-binding response regulator | NA | -6.42 |
| BA4459 | prolyl 4-hydroxylase, alpha subunit domain protein | NA | -6.28 |
| BA5689 | membrane protein, putative | NA | -6.11 |
| BA0331 | polysaccharide deacetylase-like protein | NA | -6.09 |
| BA3145 | malate dehydrogenase, putative | NA | -5.96 |
| BA5639 | D-alanyl-D-alanine carboxypeptidase, putative | NA | -5.95 |
| BA1025 | glycerol uptake facilitator protein | glpF | -5.91 |
| BA1192 | oligopeptide ABC transporter, permease protein | NA | -5.72 |
| BA3288 | impB/mucB/samB family protein | NA | -5.59 |
| BA1808 | aspartate--ammonia ligase | asnA | -5.53 |
| BA2653 | degV family protein | NA | -5.47 |
| BA1909 | branched-chain amino acid transport system II carrier protein, authentic frameshift | NA | -5.47 |
| BA5273 | hypothetical protein | NA | -5.45 |
| BA2367 | oxalate:formate antiporter, putative | NA | -5.25 |
| BA1270 | 2-oxoglutarate dehydrogenase, E1 component | odhA | -5.10 |
| BA3146 | hypothetical protein | NA | -5.05 |
| BA5274 | conserved hypothetical protein | NA | -5.01 |
| BA0802 | branched-chain amino acid transport system II carrier protein | brnQ-2 | -4.98 |
| BA3451 | serine/threonine transporter family protein | NA | -4.93 |
| BA3151 | hypothetical protein | NA | -4.89 |
| BA3153 | response regulator | NA | -4.85 |
| BA3509 | conserved hypothetical protein | NA | -4.81 |
| BA3731 | membrane protein, putative | NA | -4.80 |
| BA3266 | membrane protein, putative | NA | -4.78 |
| BA0785 | Na/Pi-cotransporter family protein | NA | -4.78 |
| BA1194 | oligopeptide ABC transporter, ATP-binding protein | NA | -4.71 |
| BA1513 | hypothetical protein | NA | -4.71 |
| BA1811 | aspartate kinase, monofunctional class | dapG-1 | -4.65 |
| BA4599 | aldehyde-alcohol dehydrogenase | NA | -4.61 |
| BA0486 | hypothetical protein | NA | -4.60 |
| BA0177 | lipoprotein, putative | NA | -4.57 |
| BA1428 | imidazole glycerol phosphate synthase, glutamine amidotransferase | hisH | -4.55 |
| BA1680 | conserved hypothetical protein | NA | -4.51 |
| BA1818 | N-acetylmuramoyl-L-alanine amidase, family 4 | NA | -4.47 |
| BXA0146 | transcriptional activator AtxA, | NA | -4.47 |
| BA1191 | oligopeptide ABC transporter, oligopeptide-binding protein | NA | -4.43 |
| BA4761 | enoyl-CoA hydratase/isomerase family protein | NA | -4.42 |
| BA1195 | oligopeptide ABC transporter, ATP-binding protein | NA | -4.41 |
| BA2846 | dltD protein | dltD-2 | -4.41 |
| BA2308 | sporulation-control protein Spo0M, putative | NA | -4.31 |
| BA1985 | hypothetical protein | NA | -4.29 |
| BA3338 | S-layer protein, putative | NA | -4.28 |
| BA3290 | hypothetical protein | NA | -4.25 |
| BA4790 | branched-chain amino acid transport system II carrier protein | brnQ-6 | -4.24 |
| BA4747 | chemotaxis protein MotB, authentic frameshift | NA | -4.20 |
| BA1976 | sensor histidine kinase | NA | -4.19 |
| BA0167 | conserved hypothetical protein | NA | -4.18 |
| BA4025 | carbamoyl-phosphate synthase, large subunit | carB | -4.11 |
| BA3156 | sodium/alanine symporter family protein, authentic frameshift | NA | -4.09 |
| BA4394 | stage 0 sporulation protein A | spo0A | -4.09 |
| BA5345 | hypothetical protein | NA | -4.07 |
| BA1247 | hypothetical protein | NA | -4.07 |
| BA3144 | conserved hypothetical protein | NA | -4.03 |
| BA3326 | conserved hypothetical protein | NA | -4.02 |
| BA1193 | oligopeptide ABC transporter, permease protein | NA | -4.02 |
| BA1293 | SinI protein | NA | -4.01 |
| BA3325 | conserved hypothetical protein | NA | -3.95 |
| BA0330 | polysaccharide deacetylase-like protein | NA | -3.92 |
| BA3202 | chaperone protein hscC | hscC | -3.91 |
| BA5292 | hypothetical protein | NA | -3.88 |
| BA2396 | conserved hypothetical protein | NA | -3.85 |
| BA2363 | transcriptional regulator, ArsR family | NA | -3.83 |
| BXA0042 | hypothetical protein, | NA | -3.83 |
| BA1983 | AMP-binding protein | NA | -3.76 |
| BA1982 | siderophore biosynthesis protein, putative | NA | -3.76 |
| BA5240 | L-lactate dehydrogenase | ldh-3 | 3.76 |
| BA3150 | spore germination protein GerAA | gerAA | -3.75 |
| BA0161 | gluconate operon transcriptional repressor | gntR | -3.73 |
| BA3157 | transporter, putative | NA | -3.72 |
| BA0694 | xanthine/uracil permease family protein | NA | -3.72 |
| BA1981 | siderophore biosynthesis protein, putative | NA | -3.71 |
| BA1862 | acetyl-CoA hydrolase/transferase family protein | NA | -3.69 |
| BA2051 | membrane protein, putative | NA | -3.69 |
| BA5054 | S-layer protein, putative | NA | -3.68 |
| BA1086 | sugar-binding transcriptional regulator, LacI family | NA | -3.64 |
| BA0665 | ribokinase | rbsK | -3.62 |
| BA5479 | glycine betaine transporter | opuD-2 | -3.60 |
| BA4022 | orotidine 5-phosphate decarboxylase | pyrF | -3.60 |
| BA3497 | prismane protein | NA | -3.59 |
| BA4825 | S-adenosylmethionine decarboxylase proenzyme | speD-1 | -3.59 |
| BA3251 | 3-oxoacyl-(acyl-carrier-protein) synthase III, putative | NA | -3.57 |
| BA5427 | endopeptidase lytE, putative | NA | -3.57 |
| BA0509 | formate acetyltransferase | pfl | -3.55 |
| BA1094 | wall-associated protein, putative | NA | -3.53 |
| BA3609 | aldehyde dehydrogenase | dhaS | -3.52 |
| BA1427 | imidazoleglycerol-phosphate dehydratase | hisB | -3.51 |
| BA1375 | ABC transporter, permease protein, putative | NA | -3.50 |
| BA2146 | nitrite reductase [NAD(P)H], large subunit | nirB | -3.50 |
| BA4901 | septation ring formation regulator | ezrA | -3.50 |
| BA1430.1 | phosphoribosyl-AMP cyclohydrolase | hisI | -3.48 |
| BA3730 | conserved hypothetical protein | NA | -3.47 |
| BA2560 | sensor histidine kinase | NA | -3.47 |
| BA0513 | conserved hypothetical protein | NA | -3.46 |
| BA4024 | dihydroorotate dehydrogenase, electron transfer subunit | pyrK | -3.46 |
| BA2957 | hypothetical protein | NA | -3.45 |
| BA5200 | transcriptional activator tipA, putative | NA | -3.45 |
| BA0668 | ribose ABC transporter, permease protein | rbsC | -3.44 |
| BA5626 | 4-oxalocrotonate tautomerase | NA | -3.43 |
| BA0213 | 1-acyl-sn-glycerol-3-phosphate acyltransferase, putative | NA | -3.43 |
| BA3425 | pentapeptide repeats domain protein | NA | -3.41 |
| BA4398 | arginine repressor | argR | -3.38 |
| BA2531 | ABC transporter, ATP-binding protein | NA | -3.36 |
| BA1390 | conserved hypothetical protein | NA | -3.36 |
| BA5696 | superoxide dismutase, Mn | sodA-2 | -3.36 |
| BA4812 | drug resistance transporter, EmrB/QacA family | NA | -3.34 |
| BA1330 | 3-oxoacyl-(acyl-carrier-protein) reductase, putative | NA | -3.32 |
| BA1799 | proton/sodium-glutamate symporter | NA | -3.31 |
| BA2956 | chorismate synthase | aroF-2 | -3.31 |
| BA5478 | hypothetical protein | NA | -3.31 |
| BA0010 | pyridoxine biosynthesis protein | NA | -3.31 |
| BA2858 | conserved hypothetical protein | NA | -3.31 |
| BA1269 | 2-oxoglutarate dehydrogenase, E2 component, dihydrolipoamide succinyltransferase | odhB | -3.31 |
| BA4167 | hypothetical protein | NA | -3.31 |
| BA2281 | arginine/ornithine antiporter | arcD | -3.30 |
| BA2992 | gamma-glutamyl phosphate reductase | proA | -3.30 |
| BA1246 | sodium/proline symporter family protein | NA | -3.27 |
| BA1812 | hypothetical protein | NA | -3.25 |
| BA5551 | ATP synthase F0, B subunit | atpF | -3.24 |
| BA3289 | conserved hypothetical protein | NA | -3.20 |
| BA5133 | aminotransferase, classes I and II | NA | -3.19 |
| BA5435 | glycosyl transferase, group 4 family protein | NA | -3.18 |
| BA5604 | LPXTG-motif cell wall anchor domain protein, degenerate | NA | -3.18 |
| BA1429 | phosphoribosylformimino-5-aminoimidazole carboxamide ribotide isomerase | hisA | -3.18 |
| BA3743 | conserved hypothetical protein | NA | -3.17 |
| BA3029 | succinylornithine transaminase, putative | NA | -3.16 |
| BA5372 | RNA polymerase sigma-54 factor | sigL | -3.16 |
| BA2599 | membrane protein, putative | NA | -3.14 |
| BA4457 | shikimate kinase | aroK | -3.14 |
| BA0408 | conserved hypothetical protein | NA | -3.13 |
| BA0204 | molybdenum ABC transporter, molybdenum-binding protein, authentic frameshift | modA | -3.12 |
| BA0692 | conserved hypothetical protein | NA | -3.11 |
| BXA0069 | hypothetical protein, | NA | -3.10 |
| BA1801 | malate oxidoreductase | ykwA | -3.10 |
| BA1685 | flagellar hook assembly protein family protein | NA | -3.09 |
| BA3989 | 3-oxoacyl-(acyl-carrier-protein) reductase | fabG | -3.08 |
| BA1243 | membrane protein, putative | NA | -3.08 |
| BA5253 | proline dehydrogenase family protein | NA | -3.08 |
| BA3935 | dihydrodipicolinate synthase | dapA-2 | -3.08 |
| BA4307 | pyrimidine-nucleoside phosphorylase | pyn-2 | -3.07 |
| BA1080 | transcriptional regulator, TetR family | NA | -3.05 |
| BA3459 | oxidoreductase, Gfo/Idh/MocA family, authentic point mutation | NA | -3.05 |
| BA3162 | 5-nucleotidase, putative | NA | -3.05 |
| BA0663 | multidrug resistance protein, putative, authentic frameshift | NA | -3.05 |
| BA5439 | chromate ion transporter | NA | -3.04 |
| BA1978 | lipoprotein, putative | NA | -3.03 |
| BA3303 | transcriptional regulator, TetR family | NA | -3.02 |
| BA4630 | hesA/moeB/thiF family protein | NA | -3.02 |
| BA4023 | dihydroorotate oxidase | pyrD | -3.01 |
| BA1984 | hypothetical protein | NA | -2.98 |
| BA5557 | uracil phosphoribosyltransferase | upp | -2.97 |
| BA1668 | conserved domain protein | NA | -2.97 |
| BA4274 | N-acetylglucosamine-6-phosphate deacetylase | nagA | -2.96 |
| BA0722 | hypothetical protein | NA | -2.95 |
| BA3154 | sensor histidine kinase, authentic frameshift | NA | -2.94 |
| BA2257 | nitroreductase family protein | NA | -2.93 |
| BA2917 | hypothetical protein | NA | -2.92 |
| BA4706 | conserved hypothetical protein | NA | -2.91 |
| BA3649 | RNA polymerase sigma-70 factor, ECF subfamily | NA | -2.91 |
| BA1501 | hypothetical protein | NA | -2.90 |
| BA3737 | N-acetylmuramoyl-L-alanine amidase, family 2 | NA | -2.89 |
| BA4193 | peptidase, M20/M25/M40 family | NA | -2.89 |
| BA2953 | 3-phosphoshikimate 1-carboxyvinyltransferase | aroA | -2.89 |
| BA2074 | conserved hypothetical protein | NA | -2.89 |
| BA2130 | ABC transporter, ATP-binding protein | NA | -2.89 |
| BA4315 | acetyltransferase, GNAT family | NA | -2.89 |
| BA5159 | pyridine nucleotide-disulphide oxidoreductase | NA | -2.89 |
| BA0703 | quinol oxidase, subunit II | qoxA | -2.88 |
| BA0428 | prophage LambdaBa04, DNA-binding protein | NA | -2.87 |
| BA0877 | conserved hypothetical protein | NA | -2.87 |
| BA1389 | D-alanine-activating enzyme/D-alanine-D-alanyl carrier protein ligase | dltA | -2.87 |
| BA1958 | oxidoreductase, short-chain dehydrogenase/reductase family | NA | -2.87 |
| BA5671 | membrane protein, putative | NA | -2.86 |
| BA1986 | conserved hypothetical protein | NA | -2.86 |
| BA0670 | transaldolase, putative | NA | -2.86 |
| BA2103 | hypothetical protein | NA | -2.85 |
| BA1481 | site-specific recombinase, phage integrase family | NA | -2.84 |
| BA4529 | PhoH family protein | NA | -2.84 |
| BA1666 | conserved hypothetical protein | NA | -2.84 |
| BA5116 | conserved hypothetical protein | NA | -2.83 |
| BA5550 | ATP synthase F1, delta subunit | atpH | -2.83 |
| BA4305 | xanthine/uracil permease family protein | NA | -2.83 |
| BA5508 | (3R)-hydroxymyristoyl-(acyl-carrier-protein) dehydratase | fabZ | -2.82 |
| BXA0145 | conserved domain protein | NA | -2.80 |
| BA0043 | 4-diphosphocytidyl-2C-methyl-D-erythritol kinase | ispE | -2.80 |
| BA1431 | phosphoribosyl-ATP pyrophosphatase | hisE | -2.79 |
| BA3928 | transcriptional regulator, GntR family | NA | -2.79 |
| BA2954 | prephenate dehydrogenase | tyrA | -2.79 |
| BXA0082 | hypothetical protein | NA | -2.79 |
| BA1826 | 3-oxoacyl-(acyl-carrier-protein) synthase III, putative | NA | -2.78 |
| BA4473 | conserved domain protein | NA | -2.77 |
| BA1683 | conserved domain protein | NA | -2.77 |
| BA0661 | glycerol-3-phosphate transporter | glpT | -2.77 |
| BA2955 | histidinol-phosphate aminotransferase | hisC-2 | -2.77 |
| BA0154 | arginase | rocF | -2.76 |
| BA1511 | glutamate dehydrogenase | gdhA | -2.76 |
| BA0724 | peptidase, M23/M37 family | NA | -2.76 |
| BA0410 | heavy metal-transporting ATPase | NA | -2.75 |
| BA0162 | gluconate kinase, authentic point mutation | NA | -2.75 |
| BA2528 | N-acetylmuramoyl-L-alanine amidase, family 3 | NA | -2.73 |
| BA0683 | undecaprenol kinase family protein | NA | -2.73 |
| BA1321 | formate/nitrite transporter family protein | NA | -2.72 |
| BA4269 | PTS system, glucose-specific IIABC component | ptsG | -2.72 |
| BA1817 | N-acetylmuramoyl-L-alanine amidase, family 3 | NA | -2.69 |
| BA3516 | amino acid permease family protein | NA | -2.68 |
| BA4649 | conserved hypothetical protein | NA | -2.68 |
| BA0796 | conserved hypothetical protein | NA | -2.68 |
| BA0414 | hypothetical protein | NA | -2.67 |
| BA4490 | ribosomal protein L33 | rpmG-2 | -2.67 |
| BA4455 | membrane protein, putative | NA | -2.66 |
| BA2267 | alcohol dehydrogenase, zinc-containing | NA | -2.63 |
| BA1953 | hydrolase, alpha/beta fold family | NA | -2.62 |
| BA0758 | disulfide bond formation protein B , putative | NA | -2.62 |
| BA5495 | ABC transporter, permease protein | NA | -2.61 |
| BA5388 | TPR domain protein | NA | -2.61 |
| BA4816 | conserved hypothetical protein | NA | -2.61 |
| BA0501 | PTS system, N-acetylglucosamine-specific IIBC component, putative | NA | -2.61 |
| BA2048 | dedA family protein | NA | -2.61 |
| BA0898 | N-acetylmuramoyl-L-alanine amidase, family 3 | NA | -2.61 |
| BA5194 | conserved hypothetical protein | NA | -2.60 |
| BA0332 | nucleoside transporter, NupC family | NA | -2.60 |
| BA1135 | cold shock protein CspA | cspA-1 | -2.60 |
| BA4284 | conserved hypothetical protein | NA | -2.56 |
| BA1537 | chorismate synthase | aroF-1 | -2.56 |
| BA4380 | mutT/nudix family protein | NA | -2.55 |
| BA0510 | pyruvate formate-lyase-activating enzyme | pflA | -2.54 |
| BA5672 | membrane protein, putative | NA | -2.54 |
| BA1865 | chlorohydrolase family protein | NA | -2.54 |
| BA4605 | conserved hypothetical protein | NA | -2.53 |
| BA5221 | ABC transporter, permease protein | NA | -2.53 |
| BA1766 | Na /H antiporter NhaC | nhaC-2 | -2.52 |
| BA4425 | conserved hypothetical protein | NA | -2.52 |
| BA0800 | ABC transporter, permease protein, putative | NA | -2.51 |
| BA2958 | chorismate mutase/phospho-2-dehydro-3-deoxyheptonate aldolase | NA | -2.51 |
| BA5431 | conserved hypothetical protein TIGR00257 | NA | -2.50 |
| BA3597 | membrane protein, putative | NA | -2.50 |
| BA1178 | conserved hypothetical protein | NA | -2.50 |
| BA4384 | 3-methyl-2-oxobutanoate dehydrogenase, alpha subunit | bfmbAa | -2.49 |
| BA5403 | hypothetical protein | NA | -2.48 |
| BA3926 | sugar ABC transporter, ATP-binding protein | NA | -2.48 |
| BA0783 | transcription antiterminator, LytR family | NA | -2.48 |
| BA5555 | conserved hypothetical protein | NA | -2.47 |
| BA1598 | conserved hypothetical protein | NA | -2.47 |
| BA4858 | thioesterase family protein | NA | -2.47 |
| BA1297 | spermidine/putrescine ABC transporter, ATP-binding protein | potA | -2.46 |
| BA4030 | pyrimidine operon regulatory protein | pyrR | -2.45 |
| BA1539 | histidinol-phosphate aminotransferase | hisC-1 | -2.45 |
| BA5071 | hypothetical protein | NA | -2.44 |
| BA2948 | ABC transporter, ATP-binding protein | NA | -2.44 |
| BA3936 | aspartate kinase, monofunctional class | dapG-2 | -2.44 |
| BA1097 | hypothetical protein | NA | -2.43 |
| BA3937 | aspartate-semialdehyde dehydrogenase | asd-2 | -2.43 |
| BA2077 | HAD-superfamily hydrolase, subfamily IIB | NA | -2.42 |
| BA3991 | fatty acid/phospholipid synthesis protein PlsX | plsX | -2.42 |
| BA1277 | conserved domain protein | NA | -2.41 |
| BXA0037 | nucleotidyltransferase domain protein, | NA | -2.41 |
| BA2899 | aminotransferase, classes I and II | NA | -2.39 |
| BA0592 | alanine dehydrogenase | ald-1 | -2.38 |
| BA1661 | chemotaxis protein CheA, authentic frameshift | NA | -2.37 |
| BA4467 | conserved hypothetical protein | NA | -2.37 |
| BA3486 | CAAX amino terminal protease family protein | NA | -2.37 |
| BA3927 | lipoprotein, Bmp family | NA | -2.37 |
| BA1746 | hypothetical protein | NA | -2.36 |
| BA5518 | glycosyl transferase, group 1 family protein | NA | -2.36 |
| BA0700 | quinol oxidase, subunit IV | qoxD | -2.36 |
| BA5517 | hypothetical protein | NA | -2.36 |
| BA4203 | EAL-domain protein | NA | -2.36 |
| BA5737 | ribonuclease P protein component | rnpA | -2.35 |
| BA5519 | glycosyl transferase, group 1 family protein | NA | -2.35 |
| BA3591 | hypothetical protein | NA | -2.34 |
| BA2239 | conserved hypothetical protein | NA | -2.34 |
| BA4308 | purine nucleoside phosphorylase | NA | -2.33 |
| BXA0156 | spore germination protein XB | NA | -2.33 |
| BA1690 | chemotaxis protein CheV, authentic frameshift | NA | -2.33 |
| BA1095 | hypothetical protein | NA | -2.33 |
| BA4895 | conserved hypothetical protein | NA | -2.32 |
| BA2314 | conserved domain protein | NA | -2.32 |
| BA4009 | guanylate kinase, putative | NA | -2.31 |
| BA5063 | hypothetical protein | NA | -2.31 |
| BA1977 | polysaccharide deacetylase, putative | NA | -2.31 |
| BA3658 | hypothetical protein | NA | -2.31 |
| BA1547 | hypothetical protein | NA | -2.31 |
| BA5494 | conserved hypothetical protein | NA | -2.30 |
| BA4386 | butyrate kinase | buk | -2.29 |
| BA1679 | flagellar motor switch protein FliG | fliG | -2.29 |
| BA3118 | metallo-beta-lactamase family protein | NA | -2.29 |
| BA4477 | UDP-N-acetylglucosamine--N-acetylmuramyl-(pentapeptide) pyrophosphoryl undecaprenol N-acetylglucosamine transferase | murG-2 | -2.28 |
| BA3845 | conserved domain protein | NA | -2.28 |
| BA1584 | conserved hypothetical protein | NA | -2.27 |
| BA5729 | stage 0 sporulation protein J | spo0J | -2.27 |
| BA3667 | 4-hydroxybenzoyl-CoA thioesterase, putative | NA | -2.26 |
| BA3365 | ABC transporter, permease protein | NA | -2.26 |
| BA4805 | RNA methyltransferase, TrmH family | NA | -2.26 |
| BA0409 | ribonuclease BN, putative | NA | -2.25 |
| BXA0199 | conserved hypothetical protein, | NA | -2.23 |
| BA4060 | acetyltransferase, GNAT family | NA | -2.22 |
| BA4453 | hypothetical protein | NA | -2.22 |
| BA2194 | hypothetical protein | NA | -2.22 |
| BA4680 | septum site-determining protein MinD | minD | -2.22 |
| BA4612 | conserved hypothetical protein TIGR00247 | NA | -2.21 |
| BA4031 | ribosomal large subunit pseudouridine synthase, RluA family | NA | -2.21 |
| BA0873 | conserved domain protein | NA | -2.21 |
| BA3744 | transketolase | tkt-2 | -2.21 |
| BA5560 | sugar-phosphate isomerase, RpiB/LacA/LacB family | NA | -2.21 |
| BA1994 | conserved domain protein | NA | -2.20 |
| BA5392 | HPr(Ser) kinase/phosphatase | hprK | -2.20 |
| BA5735 | jag protein | jag | -2.20 |
| BA2442 | conserved hypothetical protein | NA | -2.20 |
| BA5442 | PTS system, cellobiose-specific IIA component | celC-2 | -2.20 |
| BA5061 | conserved hypothetical protein | NA | -2.19 |
| BA4049 | UDP-N-acetylglucosamine--N-acetylmuramyl-(pentapeptide) pyrophosphoryl-undecaprenol N-acetylglucosamine transferase | murG-1 | -2.19 |
| BA0407 | low molecular weight phosphotyrosine protein phosphatase family protein | NA | -2.18 |
| BA1503 | Ferredoxin | fer | -2.17 |
| BA0723 | conserved domain protein | NA | -2.17 |
| BA5226 | Toprim domain protein | NA | -2.17 |
| BA0008 | inosine-5-monophosphate dehydrogenase | guaB | -2.16 |
| BA2195 | hypothetical protein | NA | -2.16 |
| BA4144 | phosphoglycerate mutase family protein | NA | -2.15 |
| BA1403 | bacitracin resistance protein | bacA-2 | -2.15 |
| BA5422 | ribosomal subunit interface protein | yfiA | -2.15 |
| BA0701 | quinol oxidase, subunit III | qoxC | -2.15 |
| BA2196 | hypothetical protein | NA | -2.15 |
| BA3098 | transcriptional regulator, IclR family | NA | -2.14 |
| BA4456 | conserved hypothetical protein | NA | -2.14 |
| BXA0036 | hypothetical protein | NA | -2.14 |
| BA0666 | ribose ABC transporter protein | rbsD | -2.14 |
| BA3367 | LPXTG-motif cell wall anchor domain protein | NA | -2.13 |
| BA1629 | cold shock protein CspB | cspB-1 | -2.13 |
| BA0159 | glucosamine--fructose-6-phosphate aminotransferase (isomerizing) | glmS | -2.12 |
| BA5738 | ribosomal protein L34 | rpmH | -2.12 |
| BA5552 | ATP synthase F0, C subunit | atpE | -2.12 |
| BA1383 | conserved domain protein | NA | -2.12 |
| BA5346 | conserved hypothetical protein | NA | -2.11 |
| BA5290 | general stress protein 20U | NA | -2.11 |
| BA2230 | conserved hypothetical protein | NA | -2.11 |
| BA1684 | conserved hypothetical protein | NA | -2.10 |
| BA4572 | conserved hypothetical protein | NA | -2.10 |
| BA3896 | conserved domain protein | NA | -2.10 |
| BA4026 | carbamoyl-phosphate synthase, small subunit | carA | -2.10 |
| BA0011 | glutamine amidotransferase, SNO family | NA | -2.10 |
| BA1674 | flagellar basal-body rod protein FlgB | flgB | -2.10 |
| BA4957 | transcriptional regulator, DeoR family | NA | -2.10 |
| BA5065 | FeoA family protein | NA | -2.09 |
| BA2572 | excinuclease ABC, A subunit-related protein | NA | -2.09 |
| BA4039 | conserved hypothetical protein TIGR00044 | NA | -2.09 |
| BA3605 | hypothetical protein | NA | -2.09 |
| BA4558 | nicotinate (nicotinamide) nucleotide adenylyltransferase | nadD | -2.09 |
| BA1185 | 3-oxoacyl-(acyl-carrier-protein) synthase II | fabF | -2.09 |
| BA1456 | sensor histidine kinase | NA | -2.09 |
| BA0111 | ribosomal protein L4 | rplD | -2.09 |
| BA0883 | polysaccharide biosynthesis protein CsaA | NA | -2.08 |
| BA2974 | malate:quinone-oxidoreductase | mqo | -2.08 |
| BA3149 | spore germination protein GerAB, authentic frameshift | gerAB | -2.08 |
| BA0404 | tellurite resistance protein, putative | NA | -2.07 |
| BXA0200 | integrase/recombinase, phage integrase family, | NA | -2.07 |
| BA3089 | transcriptional regulator, Sir2 family | NA | -2.07 |
| BXA0067 | hypothetical protein, | NA | -2.07 |
| BA2102 | conserved hypothetical protein | NA | -2.06 |
| BA4971 | molybdopterin converting factor, subunit 1 | moaD-3 | -2.06 |
| BA3648 | hypothetical protein | NA | -2.06 |
| BA4756 | conserved hypothetical protein | NA | -2.06 |
| BA0202 | molybdenum ABC transporter, permease protein | modB | -2.06 |
| BA4135 | conserved hypothetical protein | NA | -2.05 |
| BA0696 | hypothetical protein | NA | -2.04 |
| BA3387 | ABC transporter, ATP-binding protein | NA | -2.04 |
| BA1309 | glycolate oxidase, subunit GlcD | glcD | -2.04 |
| BA2193 | TPR domain protein | NA | -2.04 |
| BA5514 | glycosyl transferase, group 1 family protein | NA | -2.04 |
| BA1688 | hypothetical protein | NA | -2.03 |
| BXA0191 | conserved hypothetical protein, | NA | -2.03 |
| BA5510 | techoic acid ABC transporter, ATP-binding protein | NA | -2.03 |
| BA3645 | oligopeptide ABC transporter, oligopeptide-binding protein, putative | NA | -2.03 |
| BA3322 | conserved domain protein | NA | -2.02 |
| BA4397 | DNA repair protein RecN | recN | -2.02 |
| BA1292 | transcriptional regulator SinR | sinR | -2.02 |
| BA2291 | sensor histidine kinase | NA | -2.02 |
| BA3924 | sugar ABC transporter, permease protein, authentic frameshift | NA | -2.02 |
| BA4742 | aquaporin Z | NA | -2.02 |
| BA4938 | UDP-N-acetylmuramate--alanine ligase | murC | -2.01 |
| BA1549 | conserved hypothetical protein | NA | -2.01 |
| BA3980 | 16S rRNA processing protein RimM | rimM | -2.01 |
| BA0034 | transition state transcriptional regulatory protein AbrB | abrB | -2.01 |
| BA4670 | sensor histidine kinase, authentic frameshift | NA | -2.00 |
| SpxA1DD positively regulated genes (after 15 minutes of SpxA1DD induction) | | | |
| Locus | Gene Description | Gene Annotation | Fold Change |
| BA3525 | conserved hypothetical protein | NA | 2.00 |
| BA0910 | oligopeptide ABC transporter, permease protein | NA | 2.00 |
| BA4814 | transcriptional regulator, TetR family | NA | 2.00 |
| BA5675 | cytosolic long-chain acyl-CoA thioester hydrolase family protein | NA | 2.01 |
| BA4758 | thioredoxin | trx | 2.01 |
| BA0591 | glycerophosphoryl diester phosphodiesterase family protein | NA | 2.02 |
| BA3697 | conserved hypothetical protein | NA | 2.02 |
| BA1519 | ribosomal protein S1 | rpsA | 2.03 |
| BA4849 | DNA polymerase III, alpha subunit | dnaE | 2.04 |
| BA4830 | formamidopyrimidine-DNA glycosylase | mutM | 2.04 |
| BA0699 | sodium/hydrogen exchanger family protein/TrkA domain protein | NA | 2.04 |
| BA2359 | exonuclease SbcD, putative | NA | 2.04 |
| BA4001 | protein phosphatase 2C, family protein | NA | 2.05 |
| BXA0026 | conserved hypothetical protein, | NA | 2.06 |
| BA3942 | zinc protease, insulinase family | NA | 2.06 |
| BA4506 | membrane protein, putative | NA | 2.06 |
| BA4007 | phosphopantothenoylcysteine decarboxylase/phosphopantothenate--cysteine ligase | coaBC | 2.06 |
| BA0083 | conserved hypothetical protein | NA | 2.06 |
| BA0066 | chaperonin, 33 kDa | hslO | 2.07 |
| BA1823 | conserved hypothetical protein | NA | 2.07 |
| BA2769 | rrf2 family protein | NA | 2.08 |
| BA3391 | conserved domain protein | NA | 2.08 |
| BA3799 | conserved domain protein | NA | 2.08 |
| BA2290 | conserved hypothetical protein | NA | 2.08 |
| BA0866 | acetolactate synthase, catabolic | alsS | 2.09 |
| BA2224 | conserved hypothetical protein | NA | 2.09 |
| BA5709 | conserved hypothetical protein | NA | 2.10 |
| BA3538 | conserved hypothetical protein | NA | 2.10 |
| BA4321 | conserved hypothetical protein | NA | 2.10 |
| BA5712 | yycI protein | NA | 2.11 |
| BA3427 | conserved hypothetical protein | NA | 2.12 |
| BA0517 | conserved hypothetical protein | NA | 2.12 |
| BA4005 | polypeptide deformylase | deF-2 | 2.12 |
| BA4003 | sun protein | sun | 2.12 |
| BA4754 | succinate dehydrogenase, flavoprotein subunit | sdhA | 2.12 |
| BA2500 | hypothetical protein | NA | 2.12 |
| BA1776 | conserved hypothetical protein | NA | 2.12 |
| BA1040 | helicase, UvrD/Rep family | NA | 2.13 |
| BA4842 | fxsA cytoplasmic membrane protein, putative | NA | 2.13 |
| BA0036 | methionyl-tRNA synthetase | metS | 2.13 |
| BA3073 | conserved domain protein | NA | 2.14 |
| BA4625 | tRNA (5-methylaminomethyl-2-thiouridylate)-methyltransferase | trmU | 2.14 |
| BA5571 | modification methylase, HemK family | NA | 2.15 |
| BA5217 | ABC transporter, ATP-binding protein | NA | 2.15 |
| BA3590 | conserved hypothetical protein | NA | 2.16 |
| BA0681 | membrane protein, putative | NA | 2.16 |
| BA5066 | conserved hypothetical protein | NA | 2.16 |
| BA0745 | phospholipase, putative | NA | 2.16 |
| BA4603 | conserved hypothetical protein | NA | 2.16 |
| BA1351 | sensor histidine kinase KinD | NA | 2.16 |
| BA5187 | hypothetical protein | NA | 2.16 |
| BA4947 | peptidase, M42 family | NA | 2.17 |
| BA3063 | yaiI/yqxD family protein | NA | 2.18 |
| BA1404 | conserved hypothetical protein | NA | 2.18 |
| BA5331 | DNA-binding response regulator | NA | 2.18 |
| BA0897 | Peptidase, family M20/M25/M40 protein | NA | 2.18 |
| BA5396 | excinuclease ABC, B subunit | uvrB | 2.19 |
| BA4503 | transcriptional regulator, Fur family | NA | 2.19 |
| BA5205 | lipoic acid synthetase | lipA | 2.19 |
| BA1068 | conserved hypothetical protein | NA | 2.19 |
| BA4695 | uroporphyrinogen-III synthase | hemD | 2.20 |
| BA1073 | hypothetical protein | NA | 2.20 |
| BA5227 | hypothetical protein | NA | 2.22 |
| BA2443 | ABC transporter, ATP-binding/permease protein | NA | 2.22 |
| BA4312 | conserved hypothetical protein | NA | 2.22 |
| BA2277 | permease, putative | NA | 2.22 |
| BA0383 | ABC transporter, ATP-binding protein | NA | 2.23 |
| BA4698 | glutamyl-tRNA reductase | hemA | 2.24 |
| BA4576 | acetyltransferase, GNAT family | NA | 2.25 |
| BA5215 | aminotransferase, class V | NA | 2.25 |
| BA4271 | hydrolase, haloacid dehalogenase-like family | NA | 2.25 |
| BA5715 | DNA-binding response regulator YycF | yycF | 2.26 |
| BA5254 | conserved hypothetical protein | NA | 2.27 |
| BA0149 | kinb signaling pathway activation protein | kbaA | 2.27 |
| BA4960 | conserved hypothetical protein TIGR00275 | NA | 2.28 |
| BA1506 | CAAX amino terminal protease family protein | NA | 2.28 |
| BA5637 | conserved hypothetical protein | NA | 2.28 |
| BA2716 | conserved domain protein | NA | 2.28 |
| BA0009 | D-alanyl-D-alanine carboxypeptidase | dacA | 2.29 |
| BA2360 | exonuclease, putative | NA | 2.29 |
| BA2444 | ABC transporter, ATP-binding/permease protein | NA | 2.29 |
| BA1313 | sensor histidine kinase | NA | 2.30 |
| BA0774 | pyridine nucleotide-disulfide oxidoreductase, class I | NA | 2.30 |
| BA4328 | conserved hypothetical protein | NA | 2.30 |
| BA2358 | conserved hypothetical protein | NA | 2.31 |
| BA1546 | menaquinol-cytochrome c reductase, cytochrome b/c subunit | qcrC | 2.32 |
| BA1204 | cardiolipin synthetase | cls-2 | 2.33 |
| BA0536 | bacterioferritin comigratory protein | bcP | 2.34 |
| BA4829 | membrane protein, putative | NA | 2.34 |
| BA1541 | TPR domain protein | NA | 2.34 |
| BA4057 | S-adenosyl-methyltransferase MraW | mraW | 2.34 |
| BA2107 | formate--tetrahydrofolate ligase | fhs | 2.34 |
| BA1206 | oligoendopeptidase F | pepF-1 | 2.35 |
| BA0911 | oligopeptide ABC transporter, ATP-binding protein, authentic point mutation | NA | 2.36 |
| BA5130 | phosphoglucose isomerase | pgi | 2.36 |
| BA0082 | DNA-binding protein, putative | NA | 2.37 |
| BA5238 | PAP2 family protein | NA | 2.38 |
| BA4156 | cytochrome aa3 controlling protein | ctaA | 2.39 |
| BA4694 | delta-aminolevulinic acid dehydratase | hemB | 2.40 |
| BA2171 | PBS lyase HEAT-like repeat domain protein | NA | 2.40 |
| BA4201 | glutaredoxin family protein | NA | 2.41 |
| BA5636 | phosphate acetyltransferase | pta | 2.42 |
| BA4811 | hypothetical protein | NA | 2.42 |
| BA5567 | membrane protein, putative | NA | 2.42 |
| BA3908 | conserved hypothetical protein | NA | 2.42 |
| BA2163 | HD domain protein | NA | 2.43 |
| BA1829 | oligopeptide ABC transporter, oligopeptide-binding protein, putative, authentic frameshift | NA | 2.45 |
| BA3186 | 1,4-dihydroxy-2-naphthoate octaprenyltransferase, putative | NA | 2.45 |
| BA5189 | conserved hypothetical protein | NA | 2.46 |
| BA0653 | sulfate permease family protein | NA | 2.46 |
| BA3553 | oligoendopeptidase F | pepF-2 | 2.46 |
| BA4873 | alanine dehydrogenase | ald-2 | 2.47 |
| BA2020 | oxidoreductase, aldo/keto reductase family | NA | 2.47 |
| BA4931 | conserved hypothetical protein | NA | 2.48 |
| BA4168 | inositol monophosphatase family protein | NA | 2.48 |
| BA4217 | conserved hypothetical protein | NA | 2.49 |
| BA5201 | membrane protein, putative | NA | 2.49 |
| BA0502 | penicillin-binding protein, putative | NA | 2.49 |
| BA4240 | acetyl-CoA acetyltransferase | NA | 2.49 |
| BA5646 | hydrolase, haloacid dehalogenase-like family | NA | 2.52 |
| BA1155 | conserved hypothetical protein | NA | 2.54 |
| BXA0154 | transposase X | NA | 2.54 |
| BA4905 | conserved hypothetical protein | NA | 2.54 |
| BA4136 | PDZ domain protein | NA | 2.56 |
| BA5691 | response regulator LytR | NA | 2.57 |
| BA3666 | conserved hypothetical protein | NA | 2.57 |
| BA0834 | transcriptional regulator, TetR family | NA | 2.57 |
| BA4147 | conserved hypothetical protein | NA | 2.58 |
| BA5216 | conserved hypothetical protein | NA | 2.58 |
| BA5686 | transporter, AcrB/AcrD/AcrF family | NA | 2.60 |
| BA5625 | membrane protein, putative | NA | 2.60 |
| BA5414 | carboxyl-terminal protease | NA | 2.60 |
| BA4701 | GTP-binding protein | NA | 2.62 |
| BA0583 | acetyltransferase, GNAT family | NA | 2.62 |
| BA0590 | conserved hypothetical protein, authentic point mutation | NA | 2.65 |
| BA2044 | phosphoglycerate mutase family protein | NA | 2.66 |
| BA4306 | magnesium and cobalt transport protein CorA | corA | 2.66 |
| BA4981 | rhodanese-like domain protein | NA | 2.66 |
| BA4326 | sugar-binding transcriptional regulator, LacI family, putative | NA | 2.67 |
| BA5110 | hydrolase, alpha/beta fold family | NA | 2.68 |
| BA0544 | conserved hypothetical protein | NA | 2.68 |
| BA5141 | kinase-associated protein B | NA | 2.69 |
| BA0623 | conserved hypothetical protein | NA | 2.69 |
| BA5666 | carbon starvation protein A | cstA | 2.70 |
| BA5701 | channel protein, hemolysin III family | NA | 2.74 |
| BA3872 | peptidase T | pepT-1 | 2.75 |
| BA5568 | sua5/yciO/yrdC/ywlC family protein | NA | 2.75 |
| BA4311 | integrase/recombinase XerD | NA | 2.77 |
| BA3615 | membrane protein, putative | NA | 2.78 |
| BA0587 | acetyltransferase, GNAT family | NA | 2.78 |
| BA3698 | N-acetylmuramoyl-L-alanine amidase, putative | NA | 2.79 |
| BA1554 | conserved hypothetical protein | NA | 2.80 |
| BA0375 | DNA topoisomerase III | topB-1 | 2.80 |
| BA1557 | conserved hypothetical protein | NA | 2.81 |
| BA5692 | sensor histidine kinase LytS | NA | 2.81 |
| BA3905 | DNA mismatch repair protein MutS | mutS | 2.81 |
| BA2249 | SCO1/SenC family lipoprotein | NA | 2.81 |
| BA1495 | resB protein | resB | 2.84 |
| BA4210 | conserved hypothetical protein | NA | 2.85 |
| BA4499 | superoxide dismutase, Mn | sodA-1 | 2.85 |
| BA3524 | conserved hypothetical protein | NA | 2.85 |
| BA5155 | cytosol aminopeptidase | pepA | 2.86 |
| BA2058 | membrane protein, putative | NA | 2.86 |
| BA5289 | sodium/alanine symporter family protein | NA | 2.88 |
| BA2886 | hypothetical protein | NA | 2.91 |
| BA1559 | polyA polymerase | pcnB | 2.92 |
| BA3515 | alcohol dehydrogenase, zinc-containing, authentic point mutation | NA | 2.92 |
| BA1312 | DNA-binding response regulator | NA | 2.94 |
| BA2043 | conserved hypothetical protein | NA | 2.94 |
| BA5112 | menaquinone-specific isochorismate synthase | menF | 2.94 |
| BA1406 | transcriptional regulator, MarR family | NA | 2.97 |
| BA3344 | transcriptional regulator, MarR family | NA | 2.98 |
| BA1007 | conserved hypothetical protein | NA | 2.98 |
| BA4885 | conserved hypothetical protein | NA | 2.99 |
| BA3922 | zinc protease, insulinase family | NA | 2.99 |
| BA0527 | conserved hypothetical protein | NA | 2.99 |
| BA0499 | glutaminase A | glsA-1 | 3.01 |
| BA4936 | hypothetical protein | NA | 3.01 |
| BA1520 | isopentenyl-diphosphate delta-isomerase | NA | 3.02 |
| BA0521 | yfhP protein | NA | 3.04 |
| BA2174 | conserved hypothetical protein | NA | 3.05 |
| BA1141 | ATP-dependent nuclease, subunit B | addB | 3.05 |
| BA1111 | HD domain protein | NA | 3.06 |
| BA3876 | phosphoglycerate mutase family protein, putative | NA | 3.07 |
| BA4325 | membrane protein, putative | NA | 3.08 |
| BA4059 | 2-dehydropantoate 2-reductase | panE | 3.09 |
| BA5387 | thioredoxin reductase | trxB | 3.09 |
| BA1242 | conserved hypothetical protein | NA | 3.10 |
| BA2933 | lipoprotein, putative | NA | 3.11 |
| BA1515 | pyridine nucleotide-disulfide oxidoreductase family protein | NA | 3.11 |
| BA0862 | conserved hypothetical protein | NA | 3.12 |
| BA4935 | hypothetical protein | NA | 3.12 |
| BA3947 | tRNA pseudouridine synthase B | truB | 3.13 |
| BA0938 | conserved hypothetical protein | NA | 3.13 |
| BA0624 | norQ protein, putative | NA | 3.14 |
| BA1510 | negative regulator of competence MecA, putative | NA | 3.17 |
| BA5105 | sensor histidine kinase | NA | 3.17 |
| BA1555 | dihydrodipicolinate reductase | dapB | 3.19 |
| BA4867 | conserved domain protein | NA | 3.19 |
| BA2172 | hypothetical protein | NA | 3.21 |
| BA4318 | lolS protein | lolS | 3.22 |
| BA0381 | ABC transporter, permease protein, putative | NA | 3.23 |
| BA0079 | phosphotransferase domain protein | NA | 3.23 |
| BA4923 | oxidoreductase, Gfo/Idh/MocA family | NA | 3.27 |
| BA5048 | conserved hypothetical protein TIGR00278 | NA | 3.27 |
| BA1036 | conserved hypothetical protein | NA | 3.30 |
| BA4861 | proline dipeptidase | pepQ-2 | 3.30 |
| BA4543 | conserved hypothetical protein | NA | 3.30 |
| BA3907 | conserved hypothetical protein | NA | 3.33 |
| BA3526 | arsenical pump family protein | NA | 3.33 |
| BA5713 | YycH protein | NA | 3.34 |
| BA4058 | conserved hypothetical protein | NA | 3.35 |
| BA1302 | transporter, putative | NA | 3.35 |
| BA4988 | conserved hypothetical protein | NA | 3.35 |
| BA5255 | conserved hypothetical protein TIGR00106 | NA | 3.35 |
| BA0848 | conserved hypothetical protein | NA | 3.36 |
| BA5335 | carboxylesterase | estA | 3.37 |
| BA1496 | resC protein | resC | 3.40 |
| BA0585 | DNA-binding response regulator | NA | 3.46 |
| BA5714 | sensory box histidine kinase YycG | yycG | 3.51 |
| BA4216 | drug resistance transporter, EmrB/QacA family | NA | 3.51 |
| BA0596 | nicotinate phosphoribosyltransferase, putative | NA | 3.51 |
| BA0078 | conserved hypothetical protein | NA | 3.52 |
| BA3069 | conserved hypothetical protein | NA | 3.53 |
| BA2647 | alcohol dehydrogenase, zinc-containing | NA | 3.54 |
| BA4431 | lipoate-protein ligase A, putative | NA | 3.54 |
| BA5411 | ABC transporter, ATP-binding/permease protein | NA | 3.57 |
| BA4588 | glyoxalase family protein, authentic frameshift | NA | 3.57 |
| BA4324 | hydrolase, alpha/beta fold family, putative | NA | 3.59 |
| BA0380 | conserved hypothetical protein | NA | 3.60 |
| BA1881 | rhodanese-like domain protein | NA | 3.60 |
| BA5043 | mutT/nudix family protein | NA | 3.61 |
| BA4628 | ATPase, AAA family | NA | 3.62 |
| BA5487 | helicase, putative | NA | 3.63 |
| BA1830 | fosmidomycin resistance protein | fsR | 3.63 |
| BA2418 | protoporphyrinogen oxidase | hemY-2 | 3.68 |
| BA5148 | comA operon protein, putative | NA | 3.68 |
| BA5649 | ABC transporter, permease protein | NA | 3.70 |
| BA1962 | hypothetical protein | NA | 3.70 |
| BA5281 | conserved hypothetical protein | NA | 3.71 |
| BA5031 | conserved hypothetical protein | NA | 3.74 |
| BA2288 | CBS domain protein | NA | 3.76 |
| BA1959 | hypothetical protein | NA | 3.79 |
| BA1785 | conserved hypothetical protein | NA | 3.79 |
| BA3179 | TspO/MBR family protein | NA | 3.81 |
| BA1200 | conserved hypothetical protein | NA | 3.81 |
| BA5645 | xanthine/uracil permease family protein | NA | 3.81 |
| BA0415 | dedA family protein | NA | 3.82 |
| BA5648 | uracil-DNA glycosylase | ung | 3.83 |
| BA0080 | negative regulator of genetic competence ClpC/MecB | NA | 3.86 |
| BA0250 | holo-(acyl-carrier-protein) synthase | acpS | 3.86 |
| BA1210 | conserved hypothetical protein | NA | 3.87 |
| BA3694 | conserved hypothetical protein | NA | 3.91 |
| BA5209 | 5-nucleotidase family protein, truncation | NA | 3.94 |
| BA1296 | aldehyde dehydrogenase | ywdH | 3.98 |
| BA3335 | conserved hypothetical protein | NA | 3.99 |
| BA2016 | bacterial luciferase family protein | NA | 4.00 |
| BA2011 | hypothetical protein | NA | 4.03 |
| BA3740 | ABC transporter, ATP-binding/permease protein | NA | 4.05 |
| BA0411 | transporter, EamA family | NA | 4.05 |
| BA0975 | HD domain protein | NA | 4.07 |
| BA3655 | oxidoreductase, Gfo/Idh/MocA family | NA | 4.07 |
| BA4961 | drug resistance transporter, EmrB/QacA family | NA | 4.08 |
| BA5488 | conserved domain protein | NA | 4.10 |
| BA3874 | hypothetical protein | NA | 4.10 |
| BA0196 | oxidoreductase, aldo/keto reductase family | NA | 4.13 |
| BA3708 | transcriptional regulator, CarD family | NA | 4.13 |
| BA0081 | DNA repair protein RadA | radA | 4.15 |
| BA5491 | conserved hypothetical protein, authentic frameshift | NA | 4.16 |
| BA0493 | acetylornitine deacetylase, putative | NA | 4.17 |
| BA4945 | thioredoxin family protein | NA | 4.18 |
| BA0077 | transcriptional regulator CtsR | ctsR | 4.20 |
| BA3428 | gluconate kinase | gntK | 4.23 |
| BA4577 | hydrolase, alpha/beta fold family | NA | 4.24 |
| BA1825 | multidrug resistance protein, putative, authentic frameshift | NA | 4.26 |
| BA1558 | glycosyl transferase, group 1 family protein | NA | 4.26 |
| BA4724 | germination protein GerE | gerE | 4.26 |
| BA4541 | heat-inducible transcription repressor HrcA | hrcA | 4.28 |
| BA1505 | ATP-dependent DNA helicase RecQ | recQ-1 | 4.31 |
| BA0037 | deoxyribonuclease, TatD family | NA | 4.34 |
| BA2289 | aldehyde dehydrogenase family protein | NA | 4.36 |
| BA2947 | sulfatase | NA | 4.39 |
| BA3849 | alcohol dehydrogenase, iron-containing, authentic frameshift | NA | 4.42 |
| BA0675 | alcohol dehydrogenase, zinc-containing | NA | 4.45 |
| BA2038 | NADH:flavin oxidoreductase / NADH oxidase family protein | NA | 4.46 |
| BA1884 | 2-dehydropantoate 2-reductase | panE | 4.46 |
| BA0674 | multidrug resistance protein, putative | NA | 4.48 |
| BA2119 | glutathione peroxidase | bsaA | 4.49 |
| BA0244 | major facilitator family transporter | NA | 4.49 |
| BA3684 | alanyl-tRNA synthetase family protein | NA | 4.50 |
| BA2896 | transporter, putative | NA | 4.54 |
| BA0251 | lipoprotein, putative | NA | 4.55 |
| BA3602 | oxidoreductase, short-chain dehydrogenase/reductase family | NA | 4.63 |
| BA4868 | acetyltransferase, GNAT family | NA | 4.67 |
| BA3923 | conserved hypothetical protein | NA | 4.68 |
| BA1556 | methylglyoxal synthase | mgsA | 4.71 |
| BA3703 | phospholipase/carboxylesterase family protein | NA | 4.76 |
| BA5135 | D-isomer specific 2-hydroxyacid dehydrogenase family protein | NA | 4.81 |
| BA1038 | drug resistance transporter, EmrB/QacA family | NA | 4.85 |
| BA0197 | pyrroline-5-carboxylate reductase, putative | NA | 4.86 |
| BA3430 | transaldolase, putative | NA | 4.88 |
| BA4946 | conserved hypothetical protein | NA | 4.90 |
| BA4757 | excinuclease ABC, C subunit | uvrC | 4.91 |
| BA1767 | fumarate hydratase, class II | fumC | 4.91 |
| BA2117 | metallo-beta-lactamase family protein | NA | 4.92 |
| BA1560 | birA bifunctional protein | birA | 4.95 |
| BA5708 | conserved hypothetical protein TIGR00246 | NA | 4.99 |
| BA4359 | pyrroline-5-carboxylate reductase | proC-3 | 5.00 |
| BA2053 | cytosolic long-chain acyl-CoA thioester hydrolase family protein | NA | 5.11 |
| BA5424 | cold shock protein CspC | cspC | 5.12 |
| BA1833 | conserved domain protein | NA | 5.12 |
| BA3431 | 6-phosphogluconate dehydrogenase family protein | NA | 5.13 |
| BA3704 | glyoxylase family protein | NA | 5.14 |
| BA3741 | ABC transporter, ATP-binding/permease protein | NA | 5.16 |
| BA0528 | ABC transporter, ATP-binding/permease protein | NA | 5.21 |
| BA4739 | conserved hypothetical protein | NA | 5.22 |
| BA5650 | ABC transporter, ATP-binding protein | NA | 5.24 |
| BA0885 | S-layer protein Sap | sap | 5.31 |
| BA2867 | hypothetical protein | NA | 5.32 |
| BA1831 | cysteine synthase A | cysK-2 | 5.36 |
| BA3438 | alcohol dehydrogenase, zinc-containing | NA | 5.39 |
| BA3429 | gluconate permease | gntP-2 | 5.39 |
| BA1196 | MATE efflux family protein | NA | 5.47 |
| BA2280 | glycine betaine/L-proline ABC transporter, permease protein, putative | NA | 5.49 |
| BA1832 | acetyltransferase, GNAT family | NA | 5.52 |
| BA1775 | hypothetical protein | NA | 5.64 |
| BA1434 | D-isomer specific 2-hydroxyacid dehydrogenase family protein | NA | 5.65 |
| BA4160 | conserved hypothetical protein | NA | 5.67 |
| BA2059 | CBS domain protein | NA | 5.69 |
| BA1110 | Ser/Thr protein phosphatase family protein, authentic point mutation | NA | 5.88 |
| BA5651 | lipase/acylhydrolase, putative | NA | 5.90 |
| BA4319 | oxidoreductase, aldo/keto reductase family | NA | 5.90 |
| BA5032 | hypothetical protein | NA | 5.95 |
| BA0838 | NAD(P)H dehydrogenase, quinone family | NA | 6.32 |
| BA2357 | hypothetical protein | NA | 6.47 |
| BA2546 | conserved hypothetical protein | NA | 6.52 |
| BA0382 | ABC transporter, substrate-binding protein, putative | NA | 6.57 |
| BA3433 | glucose-6-phosphate 1-dehydrogenase | zwf | 6.60 |
| BA3798 | hypothetical protein | NA | 6.79 |
| BA1037 | major facilitator family transporter | NA | 6.83 |
| BA5687 | peptide methionine sulfoxide reductase | msrA-2 | 7.05 |
| BA4874 | 3-oxoacyl-(acyl-carrier-protein) reductase, putative | NA | 7.08 |
| BA1960 | aminoglycoside 6-adenylyltransferase, putative | NA | 7.26 |
| BA4736 | DNA-binding response regulator | NA | 7.29 |
| BA4498 | membrane protein, putative | NA | 7.47 |
| BA3416 | membrane protein, putative | NA | 7.48 |
| BA3545 | phosphoglycerate mutase, putative | NA | 7.49 |
| BA3607 | sodium/pantothenate symporter, putative | NA | 7.82 |
| BA0554 | glycine betaine transporter | opuD-1 | 7.88 |
| BA1262 | hypothetical protein | NA | 7.89 |
| BA3392 | hydrolase, haloacid dehalogenase-like family | NA | 7.96 |
| BA1208 | conserved hypothetical protein | NA | 8.14 |
| BA3432 | transketolase | tkt-1 | 8.19 |
| BA3618 | hypothetical protein | NA | 8.41 |
| BA0535 | potassium channel protein, putative | NA | 8.73 |
| BA4737 | membrane protein, putative | NA | 8.75 |
| BA0787 | major facilitator family transporter | NA | 9.01 |
| BA0532 | ABC transporter, ATP-binding protein | NA | 9.28 |
| BA0533 | ABC transporter, permease protein, putative | NA | 9.62 |
| BA1858 | major facilitator family transporter | NA | 10.00 |
| BA2279 | glycine betaine/L-proline ABC transporter, ATP-binding protein | proV-1 | 10.16 |
| BA5482 | hypothetical protein | NA | 10.25 |
| BA3543 | transcriptional regulator, LysR family | NA | 10.31 |
| BA3873 | membrane protein, putative | NA | 10.35 |
| BA1880 | transport protein, NRAMP family | NA | 10.60 |
| BA1209 | protozoan/cyanobacterial globin family protein | NA | 10.79 |
| BA4725 | xanthine/uracil permease family protein | NA | 11.61 |
| BA3614 | rarD protein | NA | 12.03 |
| BA0837 | lipoprotein, putative | NA | 12.58 |
| BA1119 | DNA-binding response regulator | NA | 12.97 |
| BA1263 | pyridine nucleotide-disulfide oxidoreductase, class I | NA | 12.98 |
| BA3620 | mrp protein | NA | 14.46 |
| BA3877 | hydrolase, alpha/beta fold family | NA | 14.57 |
| BA1118 | sensor histidine kinase | NA | 17.99 |
| BA0534 | ABC transporter, permease protein, putative | NA | 18.33 |
| BA3020 | major facilitator family transporter | NA | 20.47 |
| BA1951 | conserved hypothetical protein | NA | 32.58 |
| SpxA1DD negatively regulated genes (after 45 minutes of SpxA1DD induction) | | | |
| BA2956 | chorismate synthase | aroF-2 | -6.25 |
| BA3156 | sodium/alanine symporter family protein, authentic frameshift | NA | -6.03 |
| BA3511 | membrane protein, putative | NA | -5.78 |
| BA4747 | chemotaxis protein MotB, authentic frameshift | NA | -5.27 |
| BA3512 | membrane protein, putative | NA | -5.15 |
| BA3144 | conserved hypothetical protein | NA | -5.15 |
| BA2955 | histidinol-phosphate aminotransferase | hisC-2 | -4.83 |
| BA5442 | PTS system, cellobiose-specific IIA component | celC-2 | -4.74 |
| BA3147 | hypothetical protein | NA | -4.60 |
| BA2957 | hypothetical protein | NA | -4.25 |
| BA2954 | prephenate dehydrogenase | tyrA | -4.23 |
| BA1808 | aspartate--ammonia ligase | asnA | -4.22 |
| BA1194 | oligopeptide ABC transporter, ATP-binding protein | NA | -4.15 |
| BA1192 | oligopeptide ABC transporter, permease protein | NA | -4.12 |
| BA1025 | glycerol uptake facilitator protein | glpF | -3.92 |
| BA1094 | wall-associated protein, putative | NA | -3.91 |
| BA2528 | N-acetylmuramoyl-L-alanine amidase, family 3 | NA | -3.84 |
| BA3157 | transporter, putative | NA | -3.74 |
| BA1982 | siderophore biosynthesis protein, putative | NA | -3.67 |
| BA3145 | malate dehydrogenase, putative | NA | -3.61 |
| BA3151 | hypothetical protein | NA | -3.61 |
| BA4748 | chemotaxis protein MotA | NA | -3.61 |
| BA1195 | oligopeptide ABC transporter, ATP-binding protein | NA | -3.58 |
| BA5495 | ABC transporter, permease protein | NA | -3.57 |
| BA5639 | D-alanyl-D-alanine carboxypeptidase, putative | NA | -3.56 |
| BA4384 | 3-methyl-2-oxobutanoate dehydrogenase, alpha subunit | bfmbAa | -3.56 |
| BA5273 | hypothetical protein | NA | -3.50 |
| BA1985 | hypothetical protein | NA | -3.47 |
| BA3288 | impB/mucB/samB family protein | NA | -3.39 |
| BA3290 | hypothetical protein | NA | -3.33 |
| BA2958 | chorismate mutase/phospho-2-dehydro-3-deoxyheptonate aldolase | NA | -3.25 |
| BA5292 | hypothetical protein | NA | -3.23 |
| BA5200 | transcriptional activator tipA, putative | NA | -3.22 |
| BA3420 | conserved hypothetical protein | NA | -3.17 |
| BA3146 | hypothetical protein | NA | -3.15 |
| BA3266 | membrane protein, putative | NA | -3.14 |
| BA1427 | imidazoleglycerol-phosphate dehydratase | hisB | -3.07 |
| BA5479 | glycine betaine transporter | opuD-2 | -3.01 |
| BA3326 | conserved hypothetical protein | NA | -2.98 |
| BA2953 | 3-phosphoshikimate 1-carboxyvinyltransferase | aroA | -2.97 |
| BA4398 | arginine repressor | argR | -2.97 |
| BA3150 | spore germination protein GerAA | gerAA | -2.94 |
| BA4459 | prolyl 4-hydroxylase, alpha subunit domain protein | NA | -2.94 |
| BA2216 | sodium-dependent transporter, putative | NA | -2.94 |
| BA3143 | pyrroline-5-carboxylate reductase | proC-2 | -2.93 |
| BA1685 | flagellar hook assembly protein family protein | NA | -2.93 |
| BA1481 | site-specific recombinase, phage integrase family | NA | -2.93 |
| BA1095 | hypothetical protein | NA | -2.91 |
| BA3153 | response regulator | NA | -2.91 |
| BA1984 | hypothetical protein | NA | -2.90 |
| BA5520 | mbl protein | mbl | -2.85 |
| BA0154 | arginase | rocF | -2.85 |
| BA4386 | butyrate kinase | buk | -2.84 |
| BA4130 | prophage LambdaBa02, repressor protein | NA | -2.83 |
| BA1191 | oligopeptide ABC transporter, oligopeptide-binding protein | NA | -2.83 |
| BA5054 | S-layer protein, putative | NA | -2.81 |
| BA4167 | hypothetical protein | NA | -2.81 |
| BA3154 | sensor histidine kinase, authentic frameshift | NA | -2.81 |
| BXA0042 | hypothetical protein, | NA | -2.79 |
| BA3509 | conserved hypothetical protein | NA | -2.78 |
| BA1293 | SinI protein | NA | -2.73 |
| BA4060 | acetyltransferase, GNAT family | NA | -2.73 |
| BA3497 | prismane protein | NA | -2.73 |
| BA5345 | hypothetical protein | NA | -2.69 |
| BA0331 | polysaccharide deacetylase-like protein | NA | -2.69 |
| BA2239 | conserved hypothetical protein | NA | -2.65 |
| BXA0036 | hypothetical protein | NA | -2.63 |
| BA1983 | AMP-binding protein | NA | -2.62 |
| BA1430.1 | phosphoribosyl-AMP cyclohydrolase | hisI | -2.62 |
| BA0414 | hypothetical protein | NA | -2.62 |
| BA0694 | xanthine/uracil permease family protein | NA | -2.60 |
| BA3510 | cyclic nucleotide-binding domain protein | NA | -2.60 |
| BA5274 | conserved hypothetical protein | NA | -2.59 |
| BA2948 | ABC transporter, ATP-binding protein | NA | -2.56 |
| BXA0191 | conserved hypothetical protein, | NA | -2.55 |
| BA1026 | glycerol kinase | glpK | -2.53 |
| BA1981 | siderophore biosynthesis protein, putative | NA | -2.52 |
| BA0657 | oligopeptide ABC transporter, permease protein | NA | -2.51 |
| BA1986 | conserved hypothetical protein | NA | -2.51 |
| BA0898 | N-acetylmuramoyl-L-alanine amidase, family 3 | NA | -2.49 |
| BA1818 | N-acetylmuramoyl-L-alanine amidase, family 4 | NA | -2.48 |
| BA4490 | ribosomal protein L33 | rpmG-2 | -2.47 |
| BA3425 | pentapeptide repeats domain protein | NA | -2.46 |
| BA2599 | membrane protein, putative | NA | -2.46 |
| BA0877 | conserved hypothetical protein | NA | -2.46 |
| BA5494 | conserved hypothetical protein | NA | -2.45 |
| BA1178 | conserved hypothetical protein | NA | -2.45 |
| BA2846 | dltD protein | dltD-2 | -2.41 |
| BA5294 | conserved hypothetical protein TIGR00730 | NA | -2.40 |
| BA5162 | conserved hypothetical protein | NA | -2.39 |
| BA4816 | conserved hypothetical protein | NA | -2.39 |
| BXA0035 | group II intron reverse transcriptase/maturase, | NA | -2.38 |
| BA1428 | imidazole glycerol phosphate synthase, glutamine amidotransferase | hisH | -2.37 |
| BA0939 | membrane protein, putative | NA | -2.37 |
| BA2531 | ABC transporter, ATP-binding protein | NA | -2.36 |
| BA1975 | DNA-binding response regulator | NA | -2.34 |
| BXA0034 | conserved hypothetical protein | NA | -2.34 |
| BXA0037 | nucleotidyltransferase domain protein, | NA | -2.34 |
| BA5476 | conserved domain protein | NA | -2.33 |
| BA5221 | ABC transporter, permease protein | NA | -2.30 |
| BA5478 | hypothetical protein | NA | -2.29 |
| BA1096 | conserved hypothetical protein | NA | -2.29 |
| BA1706 | flagellin | NA | -2.27 |
| BA2560 | sensor histidine kinase | NA | -2.27 |
| BA0692 | conserved hypothetical protein | NA | -2.26 |
| BA2363 | transcriptional regulator, ArsR family | NA | -2.26 |
| BA0683 | undecaprenol kinase family protein | NA | -2.25 |
| BXA0082 | hypothetical protein | NA | -2.25 |
| BA0389 | transcriptional regulator, TetR family | NA | -2.25 |
| BA3451 | serine/threonine transporter family protein | NA | -2.24 |
| BA5072 | hypothetical protein | NA | -2.22 |
| BA2103 | hypothetical protein | NA | -2.22 |
| BA2291 | sensor histidine kinase | NA | -2.21 |
| BA1389 | D-alanine-activating enzyme/D-alanine-D-alanyl carrier protein ligase | dltA | -2.20 |
| BA4779 | conserved hypothetical protein | NA | -2.20 |
| BA4193 | peptidase, M20/M25/M40 family | NA | -2.20 |
| BA1429 | phosphoribosylformimino-5-aminoimidazole carboxamide ribotide isomerase | hisA | -2.20 |
| BA3325 | conserved hypothetical protein | NA | -2.19 |
| BA1193 | oligopeptide ABC transporter, permease protein | NA | -2.17 |
| BA1330 | 3-oxoacyl-(acyl-carrier-protein) reductase, putative | NA | -2.17 |
| BA1727 | conserved hypothetical protein | NA | -2.17 |
| BA1430 | imidazoleglycerol phosphate synthase, cyclase subunit | hisF | -2.16 |
| BA3289 | conserved hypothetical protein | NA | -2.15 |
| BA1909 | branched-chain amino acid transport system II carrier protein, authentic frameshift | NA | -2.15 |
| BA4456 | conserved hypothetical protein | NA | -2.14 |
| BA0661 | glycerol-3-phosphate transporter | glpT | -2.12 |
| BA1177 | ATP-dependent Clp protease, ATP-binding subunit ClpB | clpB | -2.12 |
| BXA0199 | conserved hypothetical protein, | NA | -2.11 |
| BA1801 | malate oxidoreductase | ykwA | -2.11 |
| BA0889 | alginate O-acetyltransferase, putative | NA | -2.10 |
| BA0330 | polysaccharide deacetylase-like protein | NA | -2.10 |
| BA3386 | conserved hypothetical protein | NA | -2.09 |
| BA4013 | fibronectin/fibrinogen-binding protein, putative | NA | -2.09 |
| BA4385 | dihydrolipoamide dehydrogenase | bfmbC | -2.09 |
| BA1431 | phosphoribosyl-ATP pyrophosphatase | hisE | -2.07 |
| BA4200 | conserved hypothetical protein | NA | -2.07 |
| BA5542 | NADH dehydrogenase I, A subunit | nuoA | -2.07 |
| BA0556 | conserved hypothetical protein | NA | -2.06 |
| BA4387 | leucine dehydrogenase | NA | -2.04 |
| BA1270 | 2-oxoglutarate dehydrogenase, E1 component | odhA | -2.04 |
| BA4031 | ribosomal large subunit pseudouridine synthase, RluA family | NA | -2.04 |
| BA3486 | CAAX amino terminal protease family protein | NA | -2.03 |
| BA0883 | polysaccharide biosynthesis protein CsaA | NA | -2.03 |
| BA3597 | membrane protein, putative | NA | -2.02 |
| BA4135 | conserved hypothetical protein | NA | -2.02 |
| BA0078 | conserved hypothetical protein | NA | -2.02 |
| BA3251 | 3-oxoacyl-(acyl-carrier-protein) synthase III, putative | NA | -2.02 |
| BA5477 | glycosyl transferase, group 2 family protein, authentic frameshift | NA | -2.02 |
| BA1380 | transcriptional regulator, AsnC family | NA | -2.01 |
| BA4203 | EAL-domain protein | NA | -2.01 |
| BA3591 | hypothetical protein | NA | -2.01 |
| BA2175 | arginyl-tRNA synthetase | argS-1 | -2.01 |
| SpxA1DD positively regulated genes (after 45 minutes of SpxA1DD induction) | | | |
| BA0909 | oligopeptide ABC transporter, permease protein | NA | 2.00 |
| BA5568 | sua5/yciO/yrdC/ywlC family protein | NA | 2.00 |
| BA3908 | conserved hypothetical protein | NA | 2.01 |
| BA1242 | conserved hypothetical protein | NA | 2.01 |
| BA4311 | integrase/recombinase XerD | NA | 2.01 |
| BA0527 | conserved hypothetical protein | NA | 2.02 |
| BA1554 | conserved hypothetical protein | NA | 2.02 |
| BA4860 | metallo-beta-lactamase family protein | NA | 2.03 |
| BA5063 | hypothetical protein | NA | 2.03 |
| BA1155 | conserved hypothetical protein | NA | 2.03 |
| BA4005 | polypeptide deformylase | deF-2 | 2.03 |
| BA0037 | deoxyribonuclease, TatD family | NA | 2.03 |
| BA4378 | conserved hypothetical protein | NA | 2.03 |
| BA5255 | conserved hypothetical protein TIGR00106 | NA | 2.03 |
| BA5488 | conserved domain protein | NA | 2.04 |
| BA0596 | nicotinate phosphoribosyltransferase, putative | NA | 2.04 |
| BA4469 | sodium:dicarboxylate symporter family protein | NA | 2.05 |
| BA2172 | hypothetical protein | NA | 2.05 |
| BA5637 | conserved hypothetical protein | NA | 2.07 |
| BA4499 | superoxide dismutase, Mn | sodA-1 | 2.07 |
| BA4543 | conserved hypothetical protein | NA | 2.08 |
| BA3391 | conserved domain protein | NA | 2.09 |
| BA4935 | hypothetical protein | NA | 2.09 |
| BA4867 | conserved domain protein | NA | 2.09 |
| BA4869 | conserved hypothetical protein | NA | 2.10 |
| BA1884 | 2-dehydropantoate 2-reductase | panE | 2.11 |
| BA2288 | CBS domain protein | NA | 2.11 |
| BA3553 | oligoendopeptidase F | pepF-2 | 2.12 |
| BA5064 | ferrous iron transport protein B | feoB | 2.13 |
| BA5411 | ABC transporter, ATP-binding/permease protein | NA | 2.13 |
| BA0415 | dedA family protein | NA | 2.13 |
| BA0912 | oligopeptide ABC transporter, ATP-binding protein | NA | 2.14 |
| BA4026 | carbamoyl-phosphate synthase, small subunit | carA | 2.14 |
| BA3922 | zinc protease, insulinase family | NA | 2.14 |
| BA0800 | ABC transporter, permease protein, putative | NA | 2.14 |
| BA0411 | transporter, EamA family | NA | 2.15 |
| BA2011 | hypothetical protein | NA | 2.15 |
| BA4551 | DNA internalization-related competence protein ComEC/Rec2 | comEC | 2.15 |
| BA5335 | carboxylesterase | estA | 2.15 |
| BA0194 | oligopeptide ABC transporter, oligopeptide-binding protein, putative | NA | 2.15 |
| BA1767 | fumarate hydratase, class II | fumC | 2.16 |
| BA0623 | conserved hypothetical protein | NA | 2.16 |
| BA0674 | multidrug resistance protein, putative | NA | 2.16 |
| BA4815 | peptidase, M42 family | NA | 2.17 |
| BA5315 | UDP-N-acetylenolpyruvoylglucosamine reductase | murB-2 | 2.18 |
| BA3698 | N-acetylmuramoyl-L-alanine amidase, putative | NA | 2.18 |
| BA5708 | conserved hypothetical protein TIGR00246 | NA | 2.19 |
| BA3655 | oxidoreductase, Gfo/Idh/MocA family | NA | 2.19 |
| BA1406 | transcriptional regulator, MarR family | NA | 2.19 |
| BA1038 | drug resistance transporter, EmrB/QacA family | NA | 2.20 |
| BA1557 | conserved hypothetical protein | NA | 2.20 |
| BA3740 | ABC transporter, ATP-binding/permease protein | NA | 2.21 |
| BA5491 | conserved hypothetical protein, authentic frameshift | NA | 2.22 |
| BA0250 | holo-(acyl-carrier-protein) synthase | acpS | 2.23 |
| BA1776 | conserved hypothetical protein | NA | 2.24 |
| BA1111 | HD domain protein | NA | 2.24 |
| BA4027 | dihydroorotase | pyrC | 2.26 |
| BA4754 | succinate dehydrogenase, flavoprotein subunit | sdhA | 2.26 |
| BA2360 | exonuclease, putative | NA | 2.26 |
| BA0502 | penicillin-binding protein, putative | NA | 2.26 |
| BA4170 | conserved hypothetical protein | NA | 2.28 |
| BA5148 | comA operon protein, putative | NA | 2.28 |
| BA2058 | membrane protein, putative | NA | 2.29 |
| BA2146 | nitrite reductase [NAD(P)H], large subunit | nirB | 2.29 |
| BA2280 | glycine betaine/L-proline ABC transporter, permease protein, putative | NA | 2.30 |
| BA4868 | acetyltransferase, GNAT family | NA | 2.31 |
| BA5714 | sensory box histidine kinase YycG | yycG | 2.31 |
| BA5112 | menaquinone-specific isochorismate synthase | menF | 2.31 |
| BA1555 | dihydrodipicolinate reductase | dapB | 2.32 |
| BA5110 | hydrolase, alpha/beta fold family | NA | 2.32 |
| BA3907 | conserved hypothetical protein | NA | 2.33 |
| BA2053 | cytosolic long-chain acyl-CoA thioester hydrolase family protein | NA | 2.33 |
| BA2289 | aldehyde dehydrogenase family protein | NA | 2.35 |
| BA1210 | conserved hypothetical protein | NA | 2.35 |
| BA0867 | alpha-acetolactate decarboxylase | alsD | 2.36 |
| BA0885 | S-layer protein Sap | sap | 2.37 |
| BA5713 | YycH protein | NA | 2.37 |
| BA3526 | arsenical pump family protein | NA | 2.38 |
| BA5692 | sensor histidine kinase LytS | NA | 2.39 |
| BA4905 | conserved hypothetical protein | NA | 2.40 |
| BA0624 | norQ protein, putative | NA | 2.41 |
| BA5105 | sensor histidine kinase | NA | 2.42 |
| BA1200 | conserved hypothetical protein | NA | 2.42 |
| BA5111 | 2-succinyl-6-hydroxy-2,4-cyclohexadiene-1-carboxylic acid synthase/2-oxoglutarate decarboxylase | menD | 2.43 |
| BA3703 | phospholipase/carboxylesterase family protein | NA | 2.44 |
| BA5648 | uracil-DNA glycosylase | ung | 2.44 |
| BA3335 | conserved hypothetical protein | NA | 2.44 |
| BA1302 | transporter, putative | NA | 2.45 |
| BA4936 | hypothetical protein | NA | 2.46 |
| BA2117 | metallo-beta-lactamase family protein | NA | 2.48 |
| BA3433 | glucose-6-phosphate 1-dehydrogenase | zwf | 2.48 |
| BA4577 | hydrolase, alpha/beta fold family | NA | 2.49 |
| BA1825 | multidrug resistance protein, putative, authentic frameshift | NA | 2.51 |
| BA4725 | xanthine/uracil permease family protein | NA | 2.52 |
| BA3590 | conserved hypothetical protein | NA | 2.52 |
| BA1434 | D-isomer specific 2-hydroxyacid dehydrogenase family protein | NA | 2.53 |
| BA5424 | cold shock protein CspC | cspC | 2.53 |
| BA1036 | conserved hypothetical protein | NA | 2.54 |
| BA3877 | hydrolase, alpha/beta fold family | NA | 2.55 |
| BA1830 | fosmidomycin resistance protein | fsR | 2.55 |
| BA0499 | glutaminase A | glsA-1 | 2.56 |
| BA5032 | hypothetical protein | NA | 2.58 |
| BA2947 | sulfatase | NA | 2.58 |
| BA4861 | proline dipeptidase | pepQ-2 | 2.59 |
| BA4945 | thioredoxin family protein | NA | 2.59 |
| BA1556 | methylglyoxal synthase | mgsA | 2.60 |
| BA0554 | glycine betaine transporter | opuD-1 | 2.60 |
| BA4160 | conserved hypothetical protein | NA | 2.61 |
| BA5675 | cytosolic long-chain acyl-CoA thioester hydrolase family protein | NA | 2.61 |
| BA1832 | acetyltransferase, GNAT family | NA | 2.61 |
| BA2059 | CBS domain protein | NA | 2.61 |
| BA4028 | aspartate carbamoyltransferase | pyrB | 2.62 |
| BA2444 | ABC transporter, ATP-binding/permease protein | NA | 2.63 |
| BA4318 | lolS protein | lolS | 2.63 |
| BA1559 | polyA polymerase | pcnB | 2.66 |
| BA2107 | formate--tetrahydrofolate ligase | fhs | 2.66 |
| BA3432 | transketolase | tkt-1 | 2.67 |
| BA0745 | phospholipase, putative | NA | 2.68 |
| BA5254 | conserved hypothetical protein | NA | 2.69 |
| BA3876 | phosphoglycerate mutase family protein, putative | NA | 2.70 |
| BA3618 | hypothetical protein | NA | 2.71 |
| BA1040 | helicase, UvrD/Rep family | NA | 2.73 |
| BA0244 | major facilitator family transporter | NA | 2.73 |
| BA0521 | yfhP protein | NA | 2.74 |
| BA0493 | acetylornitine deacetylase, putative | NA | 2.76 |
| BA2933 | lipoprotein, putative | NA | 2.77 |
| BA4780 | conserved hypothetical protein | NA | 2.77 |
| BA5289 | sodium/alanine symporter family protein | NA | 2.77 |
| BA0180 | conserved domain protein | NA | 2.79 |
| BA0911 | oligopeptide ABC transporter, ATP-binding protein, authentic point mutation | NA | 2.80 |
| BA5651 | lipase/acylhydrolase, putative | NA | 2.81 |
| BA3708 | transcriptional regulator, CarD family | NA | 2.82 |
| BA3874 | hypothetical protein | NA | 2.84 |
| BA1110 | Ser/Thr protein phosphatase family protein, authentic point mutation | NA | 2.85 |
| BA4961 | drug resistance transporter, EmrB/QacA family | NA | 2.85 |
| BA3615 | membrane protein, putative | NA | 2.86 |
| BA2281 | arginine/ornithine antiporter | arcD | 2.86 |
| BA4757 | excinuclease ABC, C subunit | uvrC | 2.88 |
| BA1881 | rhodanese-like domain protein | NA | 2.91 |
| BA5281 | conserved hypothetical protein | NA | 2.91 |
| BA3923 | conserved hypothetical protein | NA | 2.92 |
| BA1037 | major facilitator family transporter | NA | 2.94 |
| BA0510 | pyruvate formate-lyase-activating enzyme | pflA | 2.95 |
| BA2546 | conserved hypothetical protein | NA | 2.98 |
| BA1960 | aminoglycoside 6-adenylyltransferase, putative | NA | 3.01 |
| BA1558 | glycosyl transferase, group 1 family protein | NA | 3.01 |
| BA1833 | conserved domain protein | NA | 3.02 |
| BA4873 | alanine dehydrogenase | ald-2 | 3.06 |
| BA0535 | potassium channel protein, putative | NA | 3.14 |
| BA1880 | transport protein, NRAMP family | NA | 3.19 |
| BA1505 | ATP-dependent DNA helicase RecQ | recQ-1 | 3.20 |
| BA1831 | cysteine synthase A | cysK-2 | 3.21 |
| BA3063 | yaiI/yqxD family protein | NA | 3.21 |
| BA4576 | acetyltransferase, GNAT family | NA | 3.21 |
| BA3430 | transaldolase, putative | NA | 3.22 |
| BA1775 | hypothetical protein | NA | 3.23 |
| BA2896 | transporter, putative | NA | 3.26 |
| BA4874 | 3-oxoacyl-(acyl-carrier-protein) reductase, putative | NA | 3.31 |
| BA3431 | 6-phosphogluconate dehydrogenase family protein | NA | 3.31 |
| BA3545 | phosphoglycerate mutase, putative | NA | 3.36 |
| BA3614 | rarD protein | NA | 3.43 |
| BA3438 | alcohol dehydrogenase, zinc-containing | NA | 3.45 |
| BA3620 | mrp protein | NA | 3.46 |
| BA3392 | hydrolase, haloacid dehalogenase-like family | NA | 3.50 |
| BA4498 | membrane protein, putative | NA | 3.54 |
| BA1962 | hypothetical protein | NA | 3.59 |
| BA3602 | oxidoreductase, short-chain dehydrogenase/reductase family | NA | 3.60 |
| BA4319 | oxidoreductase, aldo/keto reductase family | NA | 3.72 |
| BA1560 | birA bifunctional protein | birA | 3.72 |
| BA1951 | conserved hypothetical protein | NA | 3.79 |
| BA2119 | glutathione peroxidase | bsaA | 3.86 |
| BA5209 | 5-nucleotidase family protein, truncation | NA | 3.86 |
| BA0251 | lipoprotein, putative | NA | 3.86 |
| BA1959 | hypothetical protein | NA | 3.93 |
| BA4946 | conserved hypothetical protein | NA | 4.02 |
| BA0533 | ABC transporter, permease protein, putative | NA | 4.03 |
| BA1208 | conserved hypothetical protein | NA | 4.07 |
| BA1262 | hypothetical protein | NA | 4.08 |
| BA1785 | conserved hypothetical protein | NA | 4.10 |
| BA5065 | FeoA family protein | NA | 4.14 |
| BA0532 | ABC transporter, ATP-binding protein | NA | 4.16 |
| BA0837 | lipoprotein, putative | NA | 4.25 |
| BA3020 | major facilitator family transporter | NA | 4.36 |
| BA3798 | hypothetical protein | NA | 4.61 |
| BA1196 | MATE efflux family protein | NA | 4.75 |
| BA4599 | aldehyde-alcohol dehydrogenase | NA | 4.76 |
| BA5331 | DNA-binding response regulator | NA | 4.82 |
| BA0534 | ABC transporter, permease protein, putative | NA | 4.85 |
| BA2174 | conserved hypothetical protein | NA | 5.03 |
| BA0838 | NAD(P)H dehydrogenase, quinone family | NA | 5.20 |
| BA4923 | oxidoreductase, Gfo/Idh/MocA family | NA | 5.29 |
| BA1209 | protozoan/cyanobacterial globin family protein | NA | 5.33 |
| BA5687 | peptide methionine sulfoxide reductase | msrA-2 | 5.38 |
| BA5705 | guanosine monophosphate reductase | guaC | 5.41 |
| BA4724 | germination protein GerE | gerE | 5.72 |
| BA0975 | HD domain protein | NA | 5.75 |
| BA3873 | membrane protein, putative | NA | 5.82 |
| BA0675 | alcohol dehydrogenase, zinc-containing | NA | 5.92 |
| BA3538 | conserved hypothetical protein | NA | 5.96 |
| BA3704 | glyoxylase family protein | NA | 5.96 |
| BA0787 | major facilitator family transporter | NA | 6.11 |
| BA1858 | major facilitator family transporter | NA | 6.14 |
| BA1263 | pyridine nucleotide-disulfide oxidoreductase, class I | NA | 6.35 |
| BA3799 | conserved domain protein | NA | 6.81 |
| BA3515 | alcohol dehydrogenase, zinc-containing, authentic point mutation | NA | 7.60 |
| BA2279 | glycine betaine/L-proline ABC transporter, ATP-binding protein | proV-1 | 8.67 |
| BA3694 | conserved hypothetical protein | NA | 8.69 |
| BA3849 | alcohol dehydrogenase, iron-containing, authentic frameshift | NA | 8.85 |
| BA1118 | sensor histidine kinase | NA | 11.36 |
| BA1119 | DNA-binding response regulator | NA | 12.00 |
| BA0774 | pyridine nucleotide-disulfide oxidoreductase, class I | NA | 13.74 |
