# Supplementary material for: Transcriptomic and phenotypic analysis of paralogous spx gene function in Bacillus anthracis Sterne
Source: Microbiologyopen. 2013 Jul 22;2(4):695–714. doi: 10.1002/mbo3.109 (PMC3831629; doi:10.1002/mbo3.109)
Supplement: Supplementary file 4 — Table S2. SpxA2DD-regulated genes. [file mbo30002-0695-SD4.docx]

Supplementary Table S2. SpxA2DD-regulated genes.

| SpxA2DD negatively regulated genes (after 15 minutes of SpxA2DD induction) | | | |
| --- | --- | --- | --- |
| Locus | Gene Description | Gene Annotation | Fold Change |
| BA1886 | membrane protein, putative, authentic point mutation | NA | -9.98 |
| BA3481 | hypothetical protein | NA | -9.44 |
| BA3511 | membrane protein, putative | NA | -7.59 |
| BA3144 | conserved hypothetical protein | NA | -5.87 |
| BA3896 | conserved domain protein | NA | -5.82 |
| BA3512 | membrane protein, putative | NA | -5.64 |
| BA2752 | conserved hypothetical protein | NA | -5.52 |
| BA2412 | acetyltransferase, GNAT family | NA | -5.49 |
| BA5496 | ABC transporter, ATP-binding protein | NA | -5.42 |
| BA3420 | conserved hypothetical protein | NA | -5.21 |
| BA3147 | hypothetical protein | NA | -5.10 |
| BA0822 | glucokinase regulator-related protein | NA | -5.09 |
| BA3150 | spore germination protein GerAA | gerAA | -5.08 |
| BA5298 | nucleoside transporter, NupC family | NA | -5.04 |
| BA3288 | impB/mucB/samB family protein | NA | -4.96 |
| BA1975 | DNA-binding response regulator | NA | -4.65 |
| BA5639 | D-alanyl-D-alanine carboxypeptidase, putative | NA | -4.54 |
| BA4025 | carbamoyl-phosphate synthase, large subunit | carB | -4.50 |
| BA1380 | transcriptional regulator, AsnC family | NA | -4.31 |
| BA4459 | prolyl 4-hydroxylase, alpha subunit domain protein | NA | -4.29 |
| BA2757 | conserved hypothetical protein | NA | -4.23 |
| BA4022 | orotidine 5-phosphate decarboxylase | pyrF | -4.22 |
| BA5200 | transcriptional activator tipA, putative | NA | -4.09 |
| BA1330 | 3-oxoacyl-(acyl-carrier-protein) reductase, putative | NA | -4.01 |
| BA2128 | respiratory nitrate reductase, gamma subunit | narI | -3.96 |
| BA0802 | branched-chain amino acid transport system II carrier protein | brnQ-2 | -3.95 |
| BA3737 | N-acetylmuramoyl-L-alanine amidase, family 2 | NA | -3.95 |
| BA4019 | conserved hypothetical protein | NA | -3.95 |
| BA5273 | hypothetical protein | NA | -3.91 |
| BA3157 | transporter, putative | NA | -3.90 |
| BA0738.1 | hypothetical protein | NA | -3.89 |
| BA3926 | sugar ABC transporter, ATP-binding protein | NA | -3.88 |
| BA1467 | flavohemoprotein | hmp | -3.86 |
| BA1974 | lipoprotein, putative | NA | -3.84 |
| BA1293 | SinI protein | NA | -3.81 |
| BA5072 | hypothetical protein | NA | -3.81 |
| BA0160 | conserved hypothetical protein | NA | -3.75 |
| BA4024 | dihydroorotate dehydrogenase, electron transfer subunit | pyrK | -3.73 |
| BA0610 | L-lactate permease | lldP-1 | -3.73 |
| BA3289 | conserved hypothetical protein | NA | -3.72 |
| BA2531 | ABC transporter, ATP-binding protein | NA | -3.68 |
| BA1976 | sensor histidine kinase | NA | -3.68 |
| BA3497 | prismane protein | NA | -3.64 |
| BA5523 | hypothetical protein | NA | -3.62 |
| BA1826 | 3-oxoacyl-(acyl-carrier-protein) synthase III, putative | NA | -3.59 |
| BA0812 | hypothetical protein | NA | -3.56 |
| BA0513 | conserved hypothetical protein | NA | -3.56 |
| BA1200 | conserved hypothetical protein | NA | -3.55 |
| BA2133 | molybdenum cofactor biosynthesis protein A | narA-1 | -3.55 |
| BA3451 | serine/threonine transporter family protein | NA | -3.54 |
| BA1811 | aspartate kinase, monofunctional class | dapG-1 | -3.53 |
| BA5604 | LPXTG-motif cell wall anchor domain protein, degenerate | NA | -3.51 |
| BA0428 | prophage LambdaBa04, DNA-binding protein | NA | -3.49 |
| BA1246 | sodium/proline symporter family protein | NA | -3.47 |
| BA0799 | conserved hypothetical protein | NA | -3.47 |
| BA2957 | hypothetical protein | NA | -3.46 |
| BA1390 | conserved hypothetical protein | NA | -3.44 |
| BA4023 | dihydroorotate oxidase | pyrD | -3.41 |
| BA1909 | branched-chain amino acid transport system II carrier protein, authentic frameshift | NA | -3.41 |
| BA0668 | ribose ABC transporter, permease protein | rbsC | -3.37 |
| BA1985 | hypothetical protein | NA | -3.36 |
| BA1086 | sugar-binding transcriptional regulator, LacI family | NA | -3.36 |
| BA1329 | phaR protein | NA | -3.34 |
| BA5294 | conserved hypothetical protein TIGR00730 | NA | -3.33 |
| BA1194 | oligopeptide ABC transporter, ATP-binding protein | NA | -3.32 |
| BA3356 | membrane protein, putative | NA | -3.32 |
| BA5274 | conserved hypothetical protein | NA | -3.30 |
| BA1982 | siderophore biosynthesis protein, putative | NA | -3.28 |
| BA0331 | polysaccharide deacetylase-like protein | NA | -3.27 |
| BA1727 | conserved hypothetical protein | NA | -3.26 |
| BA1984 | hypothetical protein | NA | -3.26 |
| BA5435 | glycosyl transferase, group 4 family protein | NA | -3.24 |
| BA1639 | germination protein gerN | NA | -3.24 |
| BA5345 | hypothetical protein | NA | -3.24 |
| BA1428 | imidazole glycerol phosphate synthase, glutamine amidotransferase | hisH | -3.21 |
| BA4901 | septation ring formation regulator | ezrA | -3.21 |
| BA5240 | L-lactate dehydrogenase | ldh-3 | -3.20 |
| BA2130 | ABC transporter, ATP-binding protein | NA | -3.20 |
| BA3649 | RNA polymerase sigma-70 factor, ECF subfamily | NA | -3.20 |
| BA3153 | response regulator | NA | -3.20 |
| BA2917 | hypothetical protein | NA | -3.19 |
| BA1427 | imidazoleglycerol-phosphate dehydratase | hisB | -3.17 |
| BXA0082 | hypothetical protein | NA | -3.17 |
| BA0885 | S-layer protein Sap | sap | -3.16 |
| BA0579 | malate dehydrogenase, putative | NA | -3.15 |
| BA0694 | xanthine/uracil permease family protein | NA | -3.13 |
| BA3927 | lipoprotein, Bmp family | NA | -3.13 |
| BA1270 | 2-oxoglutarate dehydrogenase, E1 component | odhA | -3.12 |
| BA0785 | Na/Pi-cotransporter family protein | NA | -3.11 |
| BA1433 | hypothetical protein | NA | -3.11 |
| BA2653 | degV family protein | NA | -3.11 |
| BA1192 | oligopeptide ABC transporter, permease protein | NA | -3.11 |
| BA0163 | gluconate permease | gntP-1 | -3.10 |
| BA0722 | hypothetical protein | NA | -3.09 |
| BA0898 | N-acetylmuramoyl-L-alanine amidase, family 3 | NA | -3.09 |
| BA5392 | HPr(Ser) kinase/phosphatase | hprK | -3.09 |
| BA5388 | TPR domain protein | NA | -3.08 |
| BA2620 | hypothetical protein | NA | -3.07 |
| BA1425 | ATP phosphoribosyltransferase | hisG | -3.06 |
| BA1812 | hypothetical protein | NA | -3.06 |
| BA0010 | pyridoxine biosynthesis protein | NA | -3.05 |
| BA4747 | chemotaxis protein MotB, authentic frameshift | NA | -3.05 |
| BXA0048 | transcriptional regulator, TetR family, | NA | -3.04 |
| BA0204 | molybdenum ABC transporter, molybdenum-binding protein, authentic frameshift | modA | -3.03 |
| BA1953 | hydrolase, alpha/beta fold family | NA | -3.03 |
| BA3326 | conserved hypothetical protein | NA | -3.00 |
| BA4394 | stage 0 sporulation protein A | spo0A | -2.99 |
| BA0981 | S-layer protein, putative | NA | -2.99 |
| BA1269 | 2-oxoglutarate dehydrogenase, E2 component, dihydrolipoamide succinyltransferase | odhB | -2.99 |
| BA3251 | 3-oxoacyl-(acyl-carrier-protein) synthase III, putative | NA | -2.97 |
| BA0330 | polysaccharide deacetylase-like protein | NA | -2.97 |
| BA1983 | AMP-binding protein | NA | -2.95 |
| BA0657 | oligopeptide ABC transporter, permease protein | NA | -2.92 |
| BA1668 | conserved domain protein | NA | -2.91 |
| BA3151 | hypothetical protein | NA | -2.91 |
| BA5194 | conserved hypothetical protein | NA | -2.91 |
| BA4284 | conserved hypothetical protein | NA | -2.91 |
| BA2846 | dltD protein | dltD-2 | -2.90 |
| BA2145 | nitrite reductase [NAD(P)H], small subunit | nirD | -2.90 |
| BA1801 | malate oxidoreductase | ykwA | -2.89 |
| BA1430.1 | phosphoribosyl-AMP cyclohydrolase | hisI | -2.89 |
| BA3146 | hypothetical protein | NA | -2.88 |
| BA3257 | transcriptional regulator, ArsR family | NA | -2.88 |
| BA5054 | S-layer protein, putative | NA | -2.87 |
| BA4193 | peptidase, M20/M25/M40 family | NA | -2.87 |
| BA3156 | sodium/alanine symporter family protein, authentic frameshift | NA | -2.87 |
| BA0154 | arginase | rocF | -2.87 |
| BA4599 | aldehyde-alcohol dehydrogenase | NA | -2.86 |
| BA3935 | dihydrodipicolinate synthase | dapA-2 | -2.85 |
| BXB0079 | IS240-related, transposase, truncation | NA | -2.85 |
| BA3744 | transketolase | tkt-2 | -2.83 |
| BA0422 | conserved hypothetical protein | NA | -2.82 |
| BA4446 | conserved hypothetical protein | NA | -2.82 |
| BA2137 | molybdopterin converting factor, subunit 1 | moaD-1 | -2.82 |
| BA1944 | cytochrome d ubiquinol oxidase, subunit II | cydB-1 | -2.80 |
| BA1782 | transposase, IS605 family, OrfA | NA | -2.80 |
| BA2308 | sporulation-control protein Spo0M, putative | NA | -2.79 |
| BA1981 | siderophore biosynthesis protein, putative | NA | -2.79 |
| BA3290 | hypothetical protein | NA | -2.78 |
| BA5550 | ATP synthase F1, delta subunit | atpH | -2.77 |
| BA2858 | conserved hypothetical protein | NA | -2.77 |
| BA0643 | amino acid ABC transporter, permease protein | NA | -2.77 |
| BXA0042 | hypothetical protein, | NA | -2.77 |
| BA3202 | chaperone protein hscC | hscC | -2.76 |
| BA3597 | membrane protein, putative | NA | -2.74 |
| BA3667 | 4-hydroxybenzoyl-CoA thioesterase, putative | NA | -2.74 |
| BA2136 | molybdopterin converting factor, subunit 2 | moaE-1 | -2.71 |
| BA0414 | hypothetical protein | NA | -2.71 |
| BA5464 | L-lactate permease | lldP-2 | -2.70 |
| BA4750 | D-alanyl-D-alanine carboxypeptidase family protein | NA | -2.69 |
| BA1469 | hypothetical protein | NA | -2.69 |
| BA5494 | conserved hypothetical protein | NA | -2.68 |
| BA1403 | bacitracin resistance protein | bacA-2 | -2.65 |
| BA1818 | N-acetylmuramoyl-L-alanine amidase, family 4 | NA | -2.65 |
| BA4398 | arginine repressor | argR | -2.64 |
| BA5116 | conserved hypothetical protein | NA | -2.63 |
| BA1862 | acetyl-CoA hydrolase/transferase family protein | NA | -2.63 |
| BA2193 | TPR domain protein | NA | -2.62 |
| BA1277 | conserved domain protein | NA | -2.61 |
| BA4130 | prophage LambdaBa02, repressor protein | NA | -2.61 |
| BA3587 | glyoxalase family protein | NA | -2.59 |
| BA1431 | phosphoribosyl-ATP pyrophosphatase | hisE | -2.58 |
| BA5372 | RNA polymerase sigma-54 factor | sigL | -2.58 |
| BA1094 | wall-associated protein, putative | NA | -2.57 |
| BA4060 | acetyltransferase, GNAT family | NA | -2.56 |
| BA2103 | hypothetical protein | NA | -2.55 |
| BA3837 | GTP-binding protein | NA | -2.54 |
| BA3743 | conserved hypothetical protein | NA | -2.54 |
| BA3937 | aspartate-semialdehyde dehydrogenase | asd-2 | -2.53 |
| BA0332 | nucleoside transporter, NupC family | NA | -2.52 |
| BA1430 | imidazoleglycerol phosphate synthase, cyclase subunit | hisF | -2.52 |
| BA1958 | oxidoreductase, short-chain dehydrogenase/reductase family | NA | -2.52 |
| BA2366 | hypothetical protein | NA | -2.52 |
| BA3731 | membrane protein, putative | NA | -2.51 |
| BA2955 | histidinol-phosphate aminotransferase | hisC-2 | -2.51 |
| BA5565 | conserved hypothetical protein | NA | -2.50 |
| BA4026 | carbamoyl-phosphate synthase, small subunit | carA | -2.50 |
| BA3509 | conserved hypothetical protein | NA | -2.50 |
| BA5479 | glycine betaine transporter | opuD-2 | -2.50 |
| BA4305 | xanthine/uracil permease family protein | NA | -2.49 |
| BA4167 | hypothetical protein | NA | -2.49 |
| BA2363 | transcriptional regulator, ArsR family | NA | -2.49 |
| BA1379 | hypothetical protein | NA | -2.49 |
| BA5292 | hypothetical protein | NA | -2.49 |
| BA1449 | peptidase, M23/M37 family | NA | -2.48 |
| BA4204 | oxidoreductase, short chain dehydrogenase/reductase family | NA | -2.48 |
| BA3925 | sugar ABC transporter, permease protein, authentic point mutation | NA | -2.48 |
| BA2992 | gamma-glutamyl phosphate reductase | proA | -2.47 |
| BA5551 | ATP synthase F0, B subunit | atpF | -2.47 |
| BA1375 | ABC transporter, permease protein, putative | NA | -2.47 |
| BA5133 | aminotransferase, classes I and II | NA | -2.46 |
| BA1331 | poly(R)-hydroxyalkanoic acid synthase, class III, PhaC subunit | phaC | -2.46 |
| BA3145 | malate dehydrogenase, putative | NA | -2.46 |
| BA0167 | conserved hypothetical protein | NA | -2.45 |
| BA1191 | oligopeptide ABC transporter, oligopeptide-binding protein | NA | -2.44 |
| BA2355 | homoserine/threonine efflux protein, putative | NA | -2.43 |
| BA1429 | phosphoribosylformimino-5-aminoimidazole carboxamide ribotide isomerase | hisA | -2.43 |
| BA0823 | PTS system, sucrose-specific IIBC component | NA | -2.43 |
| BA3645 | oligopeptide ABC transporter, oligopeptide-binding protein, putative | NA | -2.42 |
| BA0692 | conserved hypothetical protein | NA | -2.42 |
| BA4812 | drug resistance transporter, EmrB/QacA family | NA | -2.42 |
| BA0873 | conserved domain protein | NA | -2.42 |
| BA1799 | proton/sodium-glutamate symporter | NA | -2.42 |
| BA4825 | S-adenosylmethionine decarboxylase proenzyme | speD-1 | -2.41 |
| BA2442 | conserved hypothetical protein | NA | -2.41 |
| BA4307 | pyrimidine-nucleoside phosphorylase | pyn-2 | -2.41 |
| BA1193 | oligopeptide ABC transporter, permease protein | NA | -2.41 |
| BA4916 | acetoin utilization protein AcuA | acuA | -2.40 |
| BA2135 | molybdopterin biosynthesis protein MoeA | moeA-1 | -2.40 |
| BA1278 | hypothetical protein | NA | -2.39 |
| BXA0145 | conserved domain protein | NA | -2.38 |
| BA1361 | radical SAM domain protein | NA | -2.38 |
| BA2908 | transcriptional regulator, GntR family, putative, authentic frameshift | NA | -2.37 |
| BA0665 | ribokinase | rbsK | -2.36 |
| BA2226 | conserved hypothetical protein, authentic frameshift | NA | -2.36 |
| BA2954 | prephenate dehydrogenase | tyrA | -2.35 |
| BA4274 | N-acetylglucosamine-6-phosphate deacetylase | nagA | -2.34 |
| BA4706 | conserved hypothetical protein | NA | -2.33 |
| BA1943 | cytochrome d ubiquinol oxidase, subunit I | cydA-1 | -2.33 |
| BA5431 | conserved hypothetical protein TIGR00257 | NA | -2.32 |
| BA3266 | membrane protein, putative | NA | -2.31 |
| BA5557 | uracil phosphoribosyltransferase | upp | -2.31 |
| BA4456 | conserved hypothetical protein | NA | -2.31 |
| BXA0069 | hypothetical protein, | NA | -2.30 |
| BA5495 | ABC transporter, permease protein | NA | -2.30 |
| BA3407 | hypothetical protein | NA | -2.30 |
| BA0758 | disulfide bond formation protein B , putative | NA | -2.30 |
| BA2306 | hypothetical protein | NA | -2.30 |
| BA5439 | chromate ion transporter | NA | -2.29 |
| BA2389 | ABC transporter, ATP-binding protein, authentic point mutation | NA | -2.29 |
| BA1363 | conserved domain protein | NA | -2.29 |
| BA3609 | aldehyde dehydrogenase | dhaS | -2.28 |
| BA2051 | membrane protein, putative | NA | -2.28 |
| BA2878 | conserved hypothetical protein | NA | -2.28 |
| BA0396 | prolyl-tRNA synthetase | proS-1 | -2.28 |
| BA5055 | conserved domain protein | NA | -2.28 |
| BA5735 | jag protein | jag | -2.28 |
| BA1485 | conserved domain protein | NA | -2.27 |
| BA0245 | D-alanine--D-alanine ligase, authentic frameshift | NA | -2.27 |
| BA0162 | gluconate kinase, authentic point mutation | NA | -2.27 |
| BXA0146 | transcriptional activator AtxA, | NA | -2.25 |
| BA4761 | enoyl-CoA hydratase/isomerase family protein | NA | -2.25 |
| BA0701 | quinol oxidase, subunit III | qoxC | -2.25 |
| BA1817 | N-acetylmuramoyl-L-alanine amidase, family 3 | NA | -2.24 |
| BA4238 | conserved hypothetical protein | NA | -2.24 |
| BA2146 | nitrite reductase [NAD(P)H], large subunit | nirB | -2.24 |
| BA0670 | transaldolase, putative | NA | -2.24 |
| BA3258 | permease, putative | NA | -2.24 |
| BA2269 | membrane protein, putative | NA | -2.23 |
| BA1986 | conserved hypothetical protein | NA | -2.22 |
| BA4269 | PTS system, glucose-specific IIABC component | ptsG | -2.22 |
| BA1978 | lipoprotein, putative | NA | -2.22 |
| BA2441 | conserved hypothetical protein | NA | -2.21 |
| BA1135 | cold shock protein CspA | cspA-1 | -2.21 |
| BA4408 | acetyl-CoA carboxylase, biotin carboxylase | accC | -2.20 |
| BA4308 | purine nucleoside phosphorylase | NA | -2.20 |
| BA1875 | coenzyme PQQ synthesis protein, putative | NA | -2.20 |
| BA4009 | guanylate kinase, putative | NA | -2.19 |
| BA3162 | 5-nucleotidase, putative | NA | -2.19 |
| BA1682 | conserved hypothetical protein, authentic frameshift | NA | -2.19 |
| BA0853 | ABC transporter, ATP-binding/permease protein, authentic frameshift | NA | -2.18 |
| BA0366 | fatty acid desaturase | NA | -2.18 |
| BA2367 | oxalate:formate antiporter, putative | NA | -2.18 |
| BA2958 | chorismate mutase/phospho-2-dehydro-3-deoxyheptonate aldolase | NA | -2.17 |
| BA1178 | conserved hypothetical protein | NA | -2.17 |
| BA0723 | conserved domain protein | NA | -2.17 |
| BA5134 | transcriptional regulator, AsnC family | NA | -2.17 |
| BXA0035 | group II intron reverse transcriptase/maturase, | NA | -2.17 |
| BA0407 | low molecular weight phosphotyrosine protein phosphatase family protein | NA | -2.16 |
| BA4027 | dihydroorotase | pyrC | -2.16 |
| BA1080 | transcriptional regulator, TetR family | NA | -2.15 |
| BA0664 | ribose operon repressor | rbsR | -2.15 |
| BA3730 | conserved hypothetical protein | NA | -2.15 |
| BA3663 | anaerobic ribonucleoside-triphosphate reductase, putative | NA | -2.15 |
| BA1865 | chlorohydrolase family protein | NA | -2.15 |
| BA1499 | ATP:cob(I)alamin adenosyltransferase, putative | NA | -2.14 |
| BA4021 | orotate phosphoribosyltransferase | pyrE | -2.14 |
| BA5090 | permease, putative | NA | -2.14 |
| BA3510 | cyclic nucleotide-binding domain protein | NA | -2.14 |
| BA0406 | conserved hypothetical protein | NA | -2.13 |
| BA2144 | uroporphyrin-III C-methyltransferase, putative | NA | -2.12 |
| BA5253 | proline dehydrogenase family protein | NA | -2.12 |
| BA1389 | D-alanine-activating enzyme/D-alanine-D-alanyl carrier protein ligase | dltA | -2.12 |
| BA5239 | conserved hypothetical protein | NA | -2.12 |
| BA0877 | conserved hypothetical protein | NA | -2.12 |
| BA2948 | ABC transporter, ATP-binding protein | NA | -2.11 |
| BA4281 | ribT protein | ribT | -2.11 |
| BA0724 | peptidase, M23/M37 family | NA | -2.11 |
| BA3711 | urocanate hydratase | hutU | -2.10 |
| BA1243 | membrane protein, putative | NA | -2.10 |
| BA0666 | ribose ABC transporter protein | rbsD | -2.10 |
| BA2196 | hypothetical protein | NA | -2.10 |
| BA1459 | branched-chain amino acid transport system II carrier protein | brnQ-3 | -2.09 |
| BA1629 | cold shock protein CspB | cspB-1 | -2.09 |
| BA1666 | conserved hypothetical protein | NA | -2.08 |
| BA2192 | hypothetical protein | NA | -2.08 |
| BA2800 | conserved hypothetical protein | NA | -2.08 |
| BA0158 | hypothetical protein | NA | -2.08 |
| BA4790 | branched-chain amino acid transport system II carrier protein | brnQ-6 | -2.08 |
| BA5478 | hypothetical protein | NA | -2.08 |
| BA0669 | ribose ABC transporter, ribose-binding protein | rbsB | -2.08 |
| BA2956 | chorismate synthase | aroF-2 | -2.07 |
| BA0509 | formate acetyltransferase | pfl | -2.07 |
| BA2528 | N-acetylmuramoyl-L-alanine amidase, family 3 | NA | -2.07 |
| BA3486 | CAAX amino terminal protease family protein | NA | -2.07 |
| BA0619 | conserved hypothetical protein | NA | -2.06 |
| BA4813 | conserved hypothetical protein | NA | -2.05 |
| BA1690 | chemotaxis protein CheV, authentic frameshift | NA | -2.05 |
| BA2841 | conserved hypothetical protein | NA | -2.04 |
| BA2134 | molybdopterin biosynthesis protein MoeB, putative | NA | -2.03 |
| BA0246 | UDP-N-acetylmuramoylalanyl-D-glutamyl-2,6-diaminopimelate--D-alanyl-D-alanyl ligase | murF | -2.03 |
| BA1096 | conserved hypothetical protein | NA | -2.03 |
| BA0409 | ribonuclease BN, putative | NA | -2.03 |
| BA0293 | phosphoribosylformylglycinamidine synthetase I | purQ | -2.03 |
| BA3989 | 3-oxoacyl-(acyl-carrier-protein) reductase | fabG | -2.03 |
| BA3605 | hypothetical protein | NA | -2.03 |
| BA4455 | membrane protein, putative | NA | -2.02 |
| BA5689 | membrane protein, putative | NA | -2.02 |
| BA0796 | conserved hypothetical protein | NA | -2.02 |
| BA1279 | conserved hypothetical protein | NA | -2.02 |
| BA3586 | acetyltransferase, GNAT family | NA | -2.01 |
| BA4649 | conserved hypothetical protein | NA | -2.01 |
| BA3410 | membrane protein, putative | NA | -2.01 |
| BA2345 | penicillin-binding protein 1A | NA | -2.01 |
| BA0703 | quinol oxidase, subunit II | qoxA | -2.01 |
| BA1511 | glutamate dehydrogenase | gdhA | -2.01 |
| BA1684 | conserved hypothetical protein | NA | -2.00 |
| BA0816 | conserved hypothetical protein | NA | -2.00 |
| BA0557 | SPFH domain/band 7 family protein | NA | -2.00 |
| BA5427 | endopeptidase lytE, putative | NA | -2.00 |
| BA2899 | aminotransferase, classes I and II | NA | -2.00 |
| SpxA2DD positively regulated genes (after 15 minutes of SpxA2DD induction) | | | |
| Locus | Gene Description | Gene Annotation | Fold Change |
| BA0374 | conserved domain protein | NA | 2.00 |
| BA1203 | negative regulator of competence MecA | mecA | 2.00 |
| BA4505 | cation ABC transporter, ATP-binding protein, putative | NA | 2.00 |
| BA4216 | drug resistance transporter, EmrB/QacA family | NA | 2.00 |
| BA1950 | D-alanyl-D-alanine carboxypeptidase family protein | NA | 2.01 |
| BA3039 | conserved hypothetical protein | NA | 2.01 |
| BA1506 | CAAX amino terminal protease family protein | NA | 2.01 |
| BA4325 | membrane protein, putative | NA | 2.01 |
| BA5567 | membrane protein, putative | NA | 2.02 |
| BA0991 | anti-sigma b factor | rsbW | 2.02 |
| BA2490 | hypothetical protein | NA | 2.03 |
| BA4056 | cell division protein FtsL | ftsL | 2.03 |
| BA5393 | membrane protein, putative | NA | 2.03 |
| BA0426 | RNA methyltransferase, TrmA family | NA | 2.03 |
| BA1068 | conserved hypothetical protein | NA | 2.03 |
| BA5010 | bacterial transferase family protein | NA | 2.03 |
| BA2020 | oxidoreductase, aldo/keto reductase family | NA | 2.04 |
| BA0857 | amino acid ABC transporter, ATP-binding protein | NA | 2.04 |
| BA4210 | conserved hypothetical protein | NA | 2.04 |
| BA1088 | conserved hypothetical protein | NA | 2.05 |
| BA4156 | cytochrome aa3 controlling protein | ctaA | 2.05 |
| BA1526 | glycerol-3-phosphate dehydrogenase (NAD(P) ) | gpsA | 2.05 |
| BA2487 | transcriptional regulator, MarR family | NA | 2.06 |
| BA4860 | metallo-beta-lactamase family protein | NA | 2.06 |
| BA4002 | conserved hypothetical protein TIGR00048 | NA | 2.06 |
| BA4884 | conserved hypothetical protein | NA | 2.07 |
| BA1532 | GTP cyclohydrolase I | mtrA | 2.07 |
| BA1758 | transcriptional regulator, GntR family | NA | 2.07 |
| BA1242 | conserved hypothetical protein | NA | 2.08 |
| BA3697 | conserved hypothetical protein | NA | 2.08 |
| BXA0026 | conserved hypothetical protein, | NA | 2.09 |
| BA0032 | conserved hypothetical protein | NA | 2.09 |
| BA5215 | aminotransferase, class V | NA | 2.09 |
| BA4007 | phosphopantothenoylcysteine decarboxylase/phosphopantothenate--cysteine ligase | coaBC | 2.09 |
| BA5056 | membrane protein, putative | NA | 2.09 |
| BA5620 | multidrug resistance protein, putative | NA | 2.10 |
| BA4822 | primosomal protein DnaI | dnaI | 2.10 |
| BA1129 | S-layer protein, putative | NA | 2.11 |
| BA4944 | conserved hypothetical protein | NA | 2.11 |
| BA3466 | conserved hypothetical protein | NA | 2.11 |
| BA1776 | conserved hypothetical protein | NA | 2.12 |
| BA1515 | pyridine nucleotide-disulfide oxidoreductase family protein | NA | 2.12 |
| BA4852 | DHH subfamily 1 protein | NA | 2.13 |
| BA0543 | penicillin-binding domain protein | NA | 2.13 |
| BA5645 | xanthine/uracil permease family protein | NA | 2.13 |
| BA1368 | hypothetical protein | NA | 2.13 |
| BA1565 | DnaQ family exonuclease/DinG family helicase, putative | NA | 2.13 |
| BA3905 | DNA mismatch repair protein MutS | mutS | 2.13 |
| BA2967 | conserved domain protein | NA | 2.14 |
| BA4217 | conserved hypothetical protein | NA | 2.14 |
| BA0345 | alkyl hydroperoxide reductase, subunit C | ahpC | 2.14 |
| BA1155 | conserved hypothetical protein | NA | 2.14 |
| BA5191 | NAD(P)H dehydrogenase, quinone family | NA | 2.15 |
| BA4600 | cystathionine beta-lyase | metC-3 | 2.15 |
| BA5504 | DNA-binding response regulator | NA | 2.15 |
| BA2224 | conserved hypothetical protein | NA | 2.15 |
| BA1296 | aldehyde dehydrogenase | ywdH | 2.15 |
| BA4057 | S-adenosyl-methyltransferase MraW | mraW | 2.16 |
| BA5066 | conserved hypothetical protein | NA | 2.16 |
| BA4320 | conserved hypothetical protein | NA | 2.17 |
| BA4988 | conserved hypothetical protein | NA | 2.17 |
| BA5216 | conserved hypothetical protein | NA | 2.17 |
| BA5625 | membrane protein, putative | NA | 2.17 |
| BA1317 | ykgG family protein | NA | 2.17 |
| BA3740 | ABC transporter, ATP-binding/permease protein | NA | 2.18 |
| BA4345 | Na /H antiporter NhaC | nhaC-3 | 2.18 |
| BA2277 | permease, putative | NA | 2.19 |
| BA5612 | conserved hypothetical protein | NA | 2.19 |
| BA4344 | hypothetical protein | NA | 2.19 |
| BA3602 | oxidoreductase, short-chain dehydrogenase/reductase family | NA | 2.19 |
| BA3524 | conserved hypothetical protein | NA | 2.21 |
| BA4890 | thiol peroxidase | NA | 2.23 |
| BA0867 | alpha-acetolactate decarboxylase | alsD | 2.23 |
| BA5076 | hypothetical protein | NA | 2.23 |
| BA2276 | azoreductase | NA | 2.24 |
| BA5130 | phosphoglucose isomerase | pgi | 2.24 |
| BA5424 | cold shock protein CspC | cspC | 2.24 |
| BA3526 | arsenical pump family protein | NA | 2.25 |
| BA1657 | conserved hypothetical protein | NA | 2.25 |
| BA5217 | ABC transporter, ATP-binding protein | NA | 2.26 |
| BA4849 | DNA polymerase III, alpha subunit | dnaE | 2.26 |
| BA0594 | transcriptional regulator, ArsR family | NA | 2.26 |
| BA2886 | hypothetical protein | NA | 2.26 |
| BA2444 | ABC transporter, ATP-binding/permease protein | NA | 2.27 |
| BA3427 | conserved hypothetical protein | NA | 2.27 |
| BA0940 | hypothetical protein | NA | 2.27 |
| BA4577 | hydrolase, alpha/beta fold family | NA | 2.28 |
| BA2173 | conserved hypothetical protein | NA | 2.28 |
| BA4905 | conserved hypothetical protein | NA | 2.28 |
| BA4506 | membrane protein, putative | NA | 2.28 |
| BA0531 | glutamate-1-semialdehyde-2,1-aminomutase | hemL-1 | 2.28 |
| BA0520 | conserved hypothetical protein | NA | 2.29 |
| BA3655 | oxidoreductase, Gfo/Idh/MocA family | NA | 2.29 |
| BA0380 | conserved hypothetical protein | NA | 2.29 |
| BA4934 | lipoprotein, putative | NA | 2.30 |
| BA1312 | DNA-binding response regulator | NA | 2.31 |
| BA0164 | 6-phosphogluconate dehydrogenase, decarboxylating | yqjI | 2.32 |
| BA3478 | ankyrin repeat domain protein | NA | 2.32 |
| BA2867 | hypothetical protein | NA | 2.32 |
| BA3872 | peptidase T | pepT-1 | 2.32 |
| BA5637 | conserved hypothetical protein | NA | 2.32 |
| BA0415 | dedA family protein | NA | 2.32 |
| BA1554 | conserved hypothetical protein | NA | 2.33 |
| BA4054 | sporulation specific penicillin-binding protein | NA | 2.34 |
| BA0077 | transcriptional regulator CtsR | ctsR | 2.35 |
| BA0528 | ABC transporter, ATP-binding/permease protein | NA | 2.35 |
| BA3429 | gluconate permease | gntP-2 | 2.35 |
| BA1557 | conserved hypothetical protein | NA | 2.36 |
| BA5335 | carboxylesterase | estA | 2.36 |
| BA4628 | ATPase, AAA family | NA | 2.36 |
| BA2163 | HD domain protein | NA | 2.36 |
| BA3344 | transcriptional regulator, MarR family | NA | 2.37 |
| BA1143 | conserved domain protein | NA | 2.37 |
| BA2040 | hypothetical protein | NA | 2.37 |
| BA5135 | D-isomer specific 2-hydroxyacid dehydrogenase family protein | NA | 2.39 |
| BA1404 | conserved hypothetical protein | NA | 2.39 |
| BA0009 | D-alanyl-D-alanine carboxypeptidase | dacA | 2.39 |
| BA5411 | ABC transporter, ATP-binding/permease protein | NA | 2.39 |
| BA3186 | 1,4-dihydroxy-2-naphthoate octaprenyltransferase, putative | NA | 2.40 |
| BA0251 | lipoprotein, putative | NA | 2.41 |
| BA3069 | conserved hypothetical protein | NA | 2.43 |
| BA5705 | guanosine monophosphate reductase | guaC | 2.43 |
| BA2107 | formate--tetrahydrofolate ligase | fhs | 2.43 |
| BA0871 | LPXTG-motif cell wall anchor domain protein | NA | 2.43 |
| BA4960 | conserved hypothetical protein TIGR00275 | NA | 2.44 |
| BA2037 | conserved hypothetical protein | NA | 2.44 |
| BA0381 | ABC transporter, permease protein, putative | NA | 2.45 |
| BA5688 | membrane protein, putative | NA | 2.45 |
| BA3849 | alcohol dehydrogenase, iron-containing, authentic frameshift | NA | 2.45 |
| BA2288 | CBS domain protein | NA | 2.46 |
| BA4326 | sugar-binding transcriptional regulator, LacI family, putative | NA | 2.46 |
| BA5214 | nifU domain protein | NA | 2.47 |
| BA1556 | methylglyoxal synthase | mgsA | 2.47 |
| BA0583 | acetyltransferase, GNAT family | NA | 2.47 |
| BA4758 | thioredoxin | trx | 2.49 |
| BA3428 | gluconate kinase | gntK | 2.49 |
| BA2698 | conserved hypothetical protein | NA | 2.49 |
| BA2866 | MTA/SAH nucleosidase / phosphatase, putative | NA | 2.50 |
| BA3207 | conserved domain protein | NA | 2.50 |
| BA0499 | glutaminase A | glsA-1 | 2.53 |
| BXA0154 | transposase X | NA | 2.53 |
| BA5255 | conserved hypothetical protein TIGR00106 | NA | 2.53 |
| BA2009 | methyl-accepting chemotaxis protein | NA | 2.53 |
| BA0560 | response regulator, authentic frameshift | NA | 2.53 |
| BA3922 | zinc protease, insulinase family | NA | 2.54 |
| BA1231 | dTDP-4-dehydrorhamnose reductase | rfbD | 2.55 |
| BA3708 | transcriptional regulator, CarD family | NA | 2.56 |
| BA1558 | glycosyl transferase, group 1 family protein | NA | 2.57 |
| BA4879 | argininosuccinate lyase | argH-2 | 2.57 |
| BA0591 | glycerophosphoryl diester phosphodiesterase family protein | NA | 2.57 |
| BA4603 | conserved hypothetical protein | NA | 2.58 |
| BA5650 | ABC transporter, ATP-binding protein | NA | 2.58 |
| BA0699 | sodium/hydrogen exchanger family protein/TrkA domain protein | NA | 2.58 |
| BA3615 | membrane protein, putative | NA | 2.59 |
| BA5008 | conserved hypothetical protein | NA | 2.59 |
| BA0166 | conserved hypothetical protein | NA | 2.59 |
| BA4869 | conserved hypothetical protein | NA | 2.59 |
| BA4147 | conserved hypothetical protein | NA | 2.62 |
| BA1560 | birA bifunctional protein | birA | 2.62 |
| BA4005 | polypeptide deformylase | deF-2 | 2.63 |
| BA1829 | oligopeptide ABC transporter, oligopeptide-binding protein, putative, authentic frameshift | NA | 2.66 |
| BA0502 | penicillin-binding protein, putative | NA | 2.67 |
| BA1692 | conserved hypothetical protein | NA | 2.69 |
| BA0653 | sulfate permease family protein | NA | 2.71 |
| BA5001 | conserved hypothetical protein TIGR01212 | NA | 2.72 |
| BA0595 | heavy metal-transporting ATPase | NA | 2.73 |
| BA5691 | response regulator LytR | NA | 2.73 |
| BA2058 | membrane protein, putative | NA | 2.73 |
| BA4373 | conserved hypothetical protein | NA | 2.73 |
| BA5148 | comA operon protein, putative | NA | 2.74 |
| BA5043 | mutT/nudix family protein | NA | 2.75 |
| BA0081 | DNA repair protein RadA | radA | 2.75 |
| BA4306 | magnesium and cobalt transport protein CorA | corA | 2.75 |
| BA4499 | superoxide dismutase, Mn | sodA-1 | 2.75 |
| BA4881 | conserved hypothetical protein, authentic point mutation | NA | 2.76 |
| BA5692 | sensor histidine kinase LytS | NA | 2.76 |
| BA4059 | 2-dehydropantoate 2-reductase | panE | 2.76 |
| BA4936 | hypothetical protein | NA | 2.77 |
| BA1884 | 2-dehydropantoate 2-reductase | panE | 2.77 |
| BA1495 | resB protein | resB | 2.77 |
| BA2172 | hypothetical protein | NA | 2.78 |
| BA0623 | conserved hypothetical protein | NA | 2.79 |
| BA1559 | polyA polymerase | pcnB | 2.79 |
| BA2016 | bacterial luciferase family protein | NA | 2.80 |
| BA4743 | rrf2 family protein | NA | 2.82 |
| BA1555 | dihydrodipicolinate reductase | dapB | 2.82 |
| BA3133 | conserved hypothetical protein | NA | 2.84 |
| BA2171 | PBS lyase HEAT-like repeat domain protein | NA | 2.84 |
| BA4840 | conserved hypothetical protein | NA | 2.85 |
| BA4961 | drug resistance transporter, EmrB/QacA family | NA | 2.85 |
| BA4870 | hypothetical protein | NA | 2.86 |
| BA0080 | negative regulator of genetic competence ClpC/MecB | NA | 2.86 |
| BA1951 | conserved hypothetical protein | NA | 2.88 |
| BA5686 | transporter, AcrB/AcrD/AcrF family | NA | 2.89 |
| BA5666 | carbon starvation protein A | cstA | 2.89 |
| BA3947 | tRNA pseudouridine synthase B | truB | 2.90 |
| BA5648 | uracil-DNA glycosylase | ung | 2.91 |
| BA1406 | transcriptional regulator, MarR family | NA | 2.93 |
| BA0624 | norQ protein, putative | NA | 2.93 |
| BA5141 | kinase-associated protein B | NA | 2.93 |
| BA0493 | acetylornitine deacetylase, putative | NA | 2.95 |
| BA0375 | DNA topoisomerase III | topB-1 | 2.95 |
| BA0837 | lipoprotein, putative | NA | 2.95 |
| BA4885 | conserved hypothetical protein | NA | 2.96 |
| BA0079 | phosphotransferase domain protein | NA | 2.96 |
| BA0834 | transcriptional regulator, TetR family | NA | 2.96 |
| BA3614 | rarD protein | NA | 2.97 |
| BA0618 | iron compound ABC transporter, ATP-binding protein | NA | 3.00 |
| BA3923 | conserved hypothetical protein | NA | 3.01 |
| BA1505 | ATP-dependent DNA helicase RecQ | recQ-1 | 3.03 |
| BA1832 | acetyltransferase, GNAT family | NA | 3.03 |
| BA5568 | sua5/yciO/yrdC/ywlC family protein | NA | 3.04 |
| BA0745 | phospholipase, putative | NA | 3.04 |
| BA5169 | hesB/yadR/yfhF family protein | NA | 3.04 |
| BA2225 | acetyltransferase, GNAT family | NA | 3.05 |
| BA0521 | yfhP protein | NA | 3.05 |
| BA4992 | permease, putative | NA | 3.05 |
| BA1141 | ATP-dependent nuclease, subunit B | addB | 3.06 |
| BA0411 | transporter, EamA family | NA | 3.07 |
| BA2038 | NADH:flavin oxidoreductase / NADH oxidase family protein | NA | 3.07 |
| BA1264 | acetyltransferase, GNAT family | NA | 3.07 |
| BA3930 | stage III sporulation protein E | NA | 3.08 |
| BA1836 | polysaccharide deacetylase, putative | NA | 3.10 |
| BA4861 | proline dipeptidase | pepQ-2 | 3.11 |
| BA4739 | conserved hypothetical protein | NA | 3.14 |
| BA5201 | membrane protein, putative | NA | 3.18 |
| BA2119 | glutathione peroxidase | bsaA | 3.19 |
| BA5238 | PAP2 family protein | NA | 3.19 |
| BA2059 | CBS domain protein | NA | 3.20 |
| BA0848 | conserved hypothetical protein | NA | 3.20 |
| BA2174 | conserved hypothetical protein | NA | 3.21 |
| BA4312 | conserved hypothetical protein | NA | 3.23 |
| BA5137 | conserved hypothetical protein | NA | 3.24 |
| BA5651 | lipase/acylhydrolase, putative | NA | 3.26 |
| BA4935 | hypothetical protein | NA | 3.26 |
| BA5533 | NADH dehydrogenase I, M subunit | nuoM | 3.27 |
| BA0196 | oxidoreductase, aldo/keto reductase family | NA | 3.28 |
| BA1210 | conserved hypothetical protein | NA | 3.28 |
| BA4945 | thioredoxin family protein | NA | 3.29 |
| BA2011 | hypothetical protein | NA | 3.30 |
| BA0544 | conserved hypothetical protein | NA | 3.33 |
| BA3518 | drug resistance transporter, Bcr/CflA family | NA | 3.33 |
| BA5491 | conserved hypothetical protein, authentic frameshift | NA | 3.36 |
| BA3431 | 6-phosphogluconate dehydrogenase family protein | NA | 3.36 |
| BA1881 | rhodanese-like domain protein | NA | 3.38 |
| BA3672 | DNA polymerase III, epsilon subunit, putative | NA | 3.39 |
| BA4058 | conserved hypothetical protein | NA | 3.39 |
| BA1959 | hypothetical protein | NA | 3.40 |
| BA1825 | multidrug resistance protein, putative, authentic frameshift | NA | 3.41 |
| BA3209 | hypothetical protein | NA | 3.42 |
| BA3666 | conserved hypothetical protein | NA | 3.43 |
| BA0527 | conserved hypothetical protein | NA | 3.44 |
| BA3220 | transcriptional regulator, MarR family | NA | 3.45 |
| BA5488 | conserved domain protein | NA | 3.45 |
| BA0585 | DNA-binding response regulator | NA | 3.46 |
| BA4868 | acetyltransferase, GNAT family | NA | 3.47 |
| BA5031 | conserved hypothetical protein | NA | 3.47 |
| BA4431 | lipoate-protein ligase A, putative | NA | 3.49 |
| BA2896 | transporter, putative | NA | 3.50 |
| BA5482 | hypothetical protein | NA | 3.50 |
| BA4629 | prespore-specific transcriptional regulator rsfA, putative | NA | 3.50 |
| BA2546 | conserved hypothetical protein | NA | 3.51 |
| BA1833 | conserved domain protein | NA | 3.53 |
| BA3545 | phosphoglycerate mutase, putative | NA | 3.54 |
| BA4168 | inositol monophosphatase family protein | NA | 3.55 |
| BA5205 | lipoic acid synthetase | lipA | 3.55 |
| BA1962 | hypothetical protein | NA | 3.56 |
| BA3946 | riboflavin biosynthesis protein RibC | ribC | 3.57 |
| BA2792 | acetyltransferase, GNAT family | NA | 3.58 |
| BA0197 | pyrroline-5-carboxylate reductase, putative | NA | 3.59 |
| BA2117 | metallo-beta-lactamase family protein | NA | 3.61 |
| BA0235 | oligopeptide ABC transporter, ATP-binding protein | NA | 3.63 |
| BA4319 | oxidoreductase, aldo/keto reductase family | NA | 3.63 |
| BA5281 | conserved hypothetical protein | NA | 3.68 |
| BA3735 | conserved hypothetical protein, authentic frameshift | NA | 3.69 |
| BA5521 | stage III sporulation protein D | spoIIID | 3.70 |
| BA4737 | membrane protein, putative | NA | 3.72 |
| BA3430 | transaldolase, putative | NA | 3.78 |
| BA1196 | MATE efflux family protein | NA | 3.80 |
| BA2484 | conserved hypothetical protein | NA | 3.84 |
| BA5649 | ABC transporter, permease protein | NA | 3.84 |
| BA2057 | oxidoreductase, putative | NA | 3.84 |
| BA1775 | hypothetical protein | NA | 3.85 |
| BA3607 | sodium/pantothenate symporter, putative | NA | 3.85 |
| BA3261 | DNA-binding response regulator | NA | 3.85 |
| BA0675 | alcohol dehydrogenase, zinc-containing | NA | 3.89 |
| BA3868 | exodeoxyribonuclease III | exoA | 3.89 |
| BA1262 | hypothetical protein | NA | 3.89 |
| BA4218 | 5-methyltetrahydropteroyltriglutamate--homocysteine methyltransferase | metE | 3.92 |
| BA5561 | low molecular weight phosphotyrosine protein phosphatase family protein | NA | 3.93 |
| BA0382 | ABC transporter, substrate-binding protein, putative | NA | 3.97 |
| BA4541 | heat-inducible transcription repressor HrcA | hrcA | 3.98 |
| BA0800 | ABC transporter, permease protein, putative | NA | 3.98 |
| BA4160 | conserved hypothetical protein | NA | 3.99 |
| BA4738 | membrane protein, putative | NA | 3.99 |
| BA1831 | cysteine synthase A | cysK-2 | 4.02 |
| BA1510 | negative regulator of competence MecA, putative | NA | 4.04 |
| BA4324 | hydrolase, alpha/beta fold family, putative | NA | 4.05 |
| BA4588 | glyoxalase family protein, authentic frameshift | NA | 4.05 |
| BA1110 | Ser/Thr protein phosphatase family protein, authentic point mutation | NA | 4.10 |
| BA1830 | fosmidomycin resistance protein | fsR | 4.11 |
| BA3873 | membrane protein, putative | NA | 4.15 |
| BA4914 | lipoprotein, putative | NA | 4.18 |
| BA2357 | hypothetical protein | NA | 4.18 |
| BA3208 | glyoxylase family protein | NA | 4.18 |
| BA3707 | NADH-dependent flavin oxidoreductase, Oye family | NA | 4.19 |
| BA1111 | HD domain protein | NA | 4.25 |
| BA4757 | excinuclease ABC, C subunit | uvrC | 4.32 |
| BA5387 | thioredoxin reductase | trxB | 4.34 |
| BA3433 | glucose-6-phosphate 1-dehydrogenase | zwf | 4.36 |
| BA0784 | alcohol dehydrogenase, zinc-containing | NA | 4.41 |
| BA0616 | iron compound ABC transporter, permease protein | NA | 4.43 |
| BA2280 | glycine betaine/L-proline ABC transporter, permease protein, putative | NA | 4.44 |
| BA2053 | cytosolic long-chain acyl-CoA thioester hydrolase family protein | NA | 4.44 |
| BA1767 | fumarate hydratase, class II | fumC | 4.48 |
| BA5002 | conserved hypothetical protein | NA | 4.49 |
| BA1480 | hypothetical protein | NA | 4.54 |
| BA0975 | HD domain protein | NA | 4.55 |
| BA4736 | DNA-binding response regulator | NA | 4.58 |
| BA5032 | hypothetical protein | NA | 4.59 |
| BA4874 | 3-oxoacyl-(acyl-carrier-protein) reductase, putative | NA | 4.63 |
| BA5209 | 5-nucleotidase family protein, truncation | NA | 4.74 |
| BA2289 | aldehyde dehydrogenase family protein | NA | 4.83 |
| BA5048 | conserved hypothetical protein TIGR00278 | NA | 4.84 |
| BA1225 | conserved hypothetical protein | NA | 4.88 |
| BA1434 | D-isomer specific 2-hydroxyacid dehydrogenase family protein | NA | 5.03 |
| BA0674 | multidrug resistance protein, putative | NA | 5.05 |
| BA4925 | conserved domain protein | NA | 5.23 |
| BA0787 | major facilitator family transporter | NA | 5.25 |
| BA3432 | transketolase | tkt-1 | 5.32 |
| BA5687 | peptide methionine sulfoxide reductase | msrA-2 | 5.40 |
| BA0532 | ABC transporter, ATP-binding protein | NA | 5.47 |
| BA0535 | potassium channel protein, putative | NA | 5.49 |
| BA0838 | NAD(P)H dehydrogenase, quinone family | NA | 5.53 |
| BA4725 | xanthine/uracil permease family protein | NA | 5.53 |
| BA1263 | pyridine nucleotide-disulfide oxidoreductase, class I | NA | 5.90 |
| BA2279 | glycine betaine/L-proline ABC transporter, ATP-binding protein | proV-1 | 5.96 |
| BA1960 | aminoglycoside 6-adenylyltransferase, putative | NA | 6.10 |
| BA0534 | ABC transporter, permease protein, putative | NA | 6.13 |
| BA4724 | germination protein GerE | gerE | 6.25 |
| BA5675 | cytosolic long-chain acyl-CoA thioester hydrolase family protein | NA | 6.32 |
| BA1880 | transport protein, NRAMP family | NA | 6.41 |
| BA3473 | AMP-binding protein | NA | 6.44 |
| BA5208 | conserved hypothetical protein | NA | 6.72 |
| BA3438 | alcohol dehydrogenase, zinc-containing | NA | 6.85 |
| BA5543 | sensory box/GGDEF family protein | NA | 6.88 |
| BA1208 | conserved hypothetical protein | NA | 6.95 |
| BA4923 | oxidoreductase, Gfo/Idh/MocA family | NA | 6.97 |
| BA2987 | conserved hypothetical protein | NA | 7.01 |
| BA3077 | conserved hypothetical protein | NA | 7.06 |
| BA3020 | major facilitator family transporter | NA | 7.70 |
| BA4498 | membrane protein, putative | NA | 7.77 |
| BA0554 | glycine betaine transporter | opuD-1 | 7.96 |
| BA1858 | major facilitator family transporter | NA | 7.97 |
| BA3798 | hypothetical protein | NA | 7.98 |
| BA3538 | conserved hypothetical protein | NA | 8.21 |
| BA2647 | alcohol dehydrogenase, zinc-containing | NA | 8.62 |
| BA0533 | ABC transporter, permease protein, putative | NA | 10.75 |
| BA1040 | helicase, UvrD/Rep family | NA | 11.30 |
| BA3456 | conserved hypothetical protein | NA | 11.36 |
| BA0774 | pyridine nucleotide-disulfide oxidoreductase, class I | NA | 12.76 |
| BA3543 | transcriptional regulator, LysR family | NA | 12.95 |
| BA0847 | glutamate racemase | racE-1 | 15.90 |
| BA5331 | DNA-binding response regulator | NA | 16.38 |
| BA3840 | site-specific recombinase, phage integrase family | NA | 16.57 |
| BA4164 | hypothetical protein | NA | 17.64 |
| BA3515 | alcohol dehydrogenase, zinc-containing, authentic point mutation | NA | 27.30 |
| SpxA2DD negatively regulated genes (after 45 minutes of SpxA2DD induction) | | | |
| BA5054 | S-layer protein, putative | NA | -9.05 |
| BA5273 | hypothetical protein | NA | -8.56 |
| BA2757 | conserved hypothetical protein | NA | -6.93 |
| BA3511 | membrane protein, putative | NA | -5.70 |
| BA3512 | membrane protein, putative | NA | -5.24 |
| BA3145 | malate dehydrogenase, putative | NA | -5.11 |
| BA0812 | hypothetical protein | NA | -5.08 |
| BA1811 | aspartate kinase, monofunctional class | dapG-1 | -4.68 |
| BA5496 | ABC transporter, ATP-binding protein | NA | -4.37 |
| BA3146 | hypothetical protein | NA | -4.30 |
| BA3289 | conserved hypothetical protein | NA | -4.30 |
| BA1886 | membrane protein, putative, authentic point mutation | NA | -4.16 |
| BA3156 | sodium/alanine symporter family protein, authentic frameshift | NA | -3.94 |
| BA2077 | HAD-superfamily hydrolase, subfamily IIB | NA | -3.89 |
| BA4205 | Ser/Thr protein phosphatase family protein | NA | -3.82 |
| BA1812 | hypothetical protein | NA | -3.77 |
| BA5274 | conserved hypothetical protein | NA | -3.76 |
| BA2127 | nitrate reductase delta chain | narJ | -3.76 |
| BXA0082 | hypothetical protein | NA | -3.75 |
| BA4193 | peptidase, M20/M25/M40 family | NA | -3.69 |
| BA0657 | oligopeptide ABC transporter, permease protein | NA | -3.59 |
| BA2306 | hypothetical protein | NA | -3.56 |
| BA4130 | prophage LambdaBa02, repressor protein | NA | -3.50 |
| BA1467 | flavohemoprotein | hmp | 3.42 |
| BA3288 | impB/mucB/samB family protein | NA | -3.42 |
| BA0885 | S-layer protein Sap | sap | -3.38 |
| BA0799 | conserved hypothetical protein | NA | -3.35 |
| BA4778 | hypothetical protein | NA | -3.20 |
| BA0331 | polysaccharide deacetylase-like protein | NA | -3.19 |
| BA1330 | 3-oxoacyl-(acyl-carrier-protein) reductase, putative | NA | -3.17 |
| BA2136 | molybdopterin converting factor, subunit 2 | moaE-1 | -3.17 |
| BA1875 | coenzyme PQQ synthesis protein, putative | NA | -3.16 |
| BA2135 | molybdopterin biosynthesis protein MoeA | moeA-1 | -3.14 |
| BA0683 | undecaprenol kinase family protein | NA | -3.11 |
| BA1329 | phaR protein | NA | -3.10 |
| BA4224 | hypothetical protein | NA | -3.08 |
| BA3147 | hypothetical protein | NA | -3.08 |
| BA0694 | xanthine/uracil permease family protein | NA | -3.06 |
| BA1661 | chemotaxis protein CheA, authentic frameshift | NA | -3.06 |
| BA3497 | prismane protein | NA | 3.05 |
| BA5071 | hypothetical protein | NA | -3.01 |
| BA3144 | conserved hypothetical protein | NA | -3.01 |
| BA5239 | conserved hypothetical protein | NA | -3.00 |
| BA2948 | ABC transporter, ATP-binding protein | NA | -2.98 |
| BA5604 | LPXTG-motif cell wall anchor domain protein, degenerate | NA | -2.98 |
| BA3326 | conserved hypothetical protein | NA | -2.98 |
| BA4747 | chemotaxis protein MotB, authentic frameshift | NA | -2.95 |
| BA1426 | histidinol dehydrogenase | hisD | -2.91 |
| BXA0051 | hypothetical protein | NA | -2.90 |
| BA2133 | molybdenum cofactor biosynthesis protein A | narA-1 | -2.86 |
| BA1909 | branched-chain amino acid transport system II carrier protein, authentic frameshift | NA | -2.85 |
| BXA0036 | hypothetical protein | NA | -2.84 |
| BXA0079 | surface layer protein, | NA | -2.84 |
| BA1380 | transcriptional regulator, AsnC family | NA | -2.83 |
| BA2103 | hypothetical protein | NA | -2.81 |
| BA3202 | chaperone protein hscC | hscC | -2.80 |
| BA4398 | arginine repressor | argR | -2.79 |
| BA1481 | site-specific recombinase, phage integrase family | NA | -2.79 |
| BA2800 | conserved hypothetical protein | NA | -2.78 |
| BA4914 | lipoprotein, putative | NA | -2.78 |
| BA0822 | glucokinase regulator-related protein | NA | -2.77 |
| BA1727 | conserved hypothetical protein | NA | -2.74 |
| BA2041 | oligopeptide ABC transporter, oligopeptide-binding protein, putative | NA | -2.72 |
| BA4011 | conserved hypothetical protein TIGR00255 | NA | -2.70 |
| BA0898 | N-acetylmuramoyl-L-alanine amidase, family 3 | NA | -2.69 |
| BA1782 | transposase, IS605 family, OrfA | NA | -2.68 |
| BA3150 | spore germination protein GerAA | gerAA | -2.65 |
| BA1097 | hypothetical protein | NA | -2.64 |
| BA5240 | L-lactate dehydrogenase | ldh-3 | -2.63 |
| BA5639 | D-alanyl-D-alanine carboxypeptidase, putative | NA | -2.61 |
| BA3258 | permease, putative | NA | -2.61 |
| BA1684 | conserved hypothetical protein | NA | -2.60 |
| BA1200 | conserved hypothetical protein | NA | -2.59 |
| BA1668 | conserved domain protein | NA | -2.59 |
| BA2355 | homoserine/threonine efflux protein, putative | NA | -2.58 |
| BA5055 | conserved domain protein | NA | -2.58 |
| BA2916 | membrane protein, putative | NA | -2.57 |
| BA4200 | conserved hypothetical protein | NA | -2.57 |
| BA4748 | chemotaxis protein MotA | NA | -2.56 |
| BA2956 | chorismate synthase | aroF-2 | -2.55 |
| BA5276 | sensor histidine kinase | NA | 2.55 |
| BXA0069 | hypothetical protein, | NA | -2.55 |
| BA2146 | nitrite reductase [NAD(P)H], large subunit | nirB | -2.55 |
| BA1897 | conserved hypothetical protein | NA | -2.55 |
| BA3837 | GTP-binding protein | NA | -2.55 |
| BXA0042 | hypothetical protein, | NA | -2.54 |
| BA1293 | SinI protein | NA | -2.54 |
| BA1403 | bacitracin resistance protein | bacA-2 | -2.54 |
| BA2125 | respiratory nitrate reductase, alpha subunit | narG | -2.53 |
| BA2621 | hypothetical protein | NA | -2.52 |
| BA0038 | primase-related protein | NA | -2.50 |
| BA4450 | helicase, putative | NA | -2.49 |
| BA0406 | conserved hypothetical protein | NA | -2.49 |
| BA5495 | ABC transporter, permease protein | NA | -2.49 |
| BA2130 | ABC transporter, ATP-binding protein | NA | -2.47 |
| BA4779 | conserved hypothetical protein | NA | -2.47 |
| BA1862 | acetyl-CoA hydrolase/transferase family protein | NA | -2.44 |
| BA4769 | spore coat protein C, putative | NA | -2.42 |
| BA2111 | glyoxalase family protein | NA | -2.41 |
| BA5294 | conserved hypothetical protein TIGR00730 | NA | -2.41 |
| BA5514 | glycosyl transferase, group 1 family protein | NA | -2.41 |
| BA5200 | transcriptional activator tipA, putative | NA | -2.39 |
| BA3587 | glyoxalase family protein | NA | -2.39 |
| BA3663 | anaerobic ribonucleoside-triphosphate reductase, putative | NA | -2.39 |
| BA3570 | hypothetical protein | NA | -2.39 |
| BA0422 | conserved hypothetical protein | NA | -2.38 |
| BXA0034 | conserved hypothetical protein | NA | -2.38 |
| BA0619 | conserved hypothetical protein | NA | -2.38 |
| BA1331 | poly(R)-hydroxyalkanoic acid synthase, class III, PhaC subunit | phaC | -2.37 |
| BA4169 | conserved hypothetical protein | NA | -2.37 |
| BA0160 | conserved hypothetical protein | NA | -2.37 |
| BA0823 | PTS system, sucrose-specific IIBC component | NA | -2.36 |
| BA0351 | iron compound ABC transporter, iron compound-binding protein | NA | -2.36 |
| BA0722 | hypothetical protein | NA | -2.36 |
| BXA0037 | nucleotidyltransferase domain protein, | NA | -2.35 |
| BXA0035 | group II intron reverse transcriptase/maturase, | NA | -2.34 |
| BA1667 | conserved hypothetical protein | NA | -2.34 |
| BA2389 | ABC transporter, ATP-binding protein, authentic point mutation | NA | -2.33 |
| BA0270 | xanthine/uracil permease family protein | NA | -2.32 |
| BA0643 | amino acid ABC transporter, permease protein | NA | -2.32 |
| BA2917 | hypothetical protein | NA | -2.32 |
| BA0204 | molybdenum ABC transporter, molybdenum-binding protein, authentic frameshift | modA | -2.31 |
| BA0414 | hypothetical protein | NA | -2.31 |
| BA5685 | transcriptional regulator, TetR family | NA | -2.31 |
| BXA0199 | conserved hypothetical protein, | NA | -2.30 |
| BA1379 | hypothetical protein | NA | -2.30 |
| BA1685 | flagellar hook assembly protein family protein | NA | -2.29 |
| BA3386 | conserved hypothetical protein | NA | -2.29 |
| BA2953 | 3-phosphoshikimate 1-carboxyvinyltransferase | aroA | -2.29 |
| BA4459 | prolyl 4-hydroxylase, alpha subunit domain protein | NA | -2.29 |
| BA2442 | conserved hypothetical protein | NA | -2.28 |
| BA1741 | hypothetical protein | NA | -2.28 |
| BXA0190 | hypothetical protein, | NA | -2.28 |
| BA0428 | prophage LambdaBa04, DNA-binding protein | NA | -2.28 |
| BA4605 | conserved hypothetical protein | NA | -2.28 |
| BA4599 | aldehyde-alcohol dehydrogenase | NA | -2.28 |
| BA2531 | ABC transporter, ATP-binding protein | NA | -2.27 |
| BA4706 | conserved hypothetical protein | NA | 2.27 |
| BA0877 | conserved hypothetical protein | NA | -2.27 |
| BXA0057 | hypothetical protein | NA | -2.26 |
| BA3407 | hypothetical protein | NA | -2.26 |
| BA2954 | prephenate dehydrogenase | tyrA | -2.25 |
| BA1375 | ABC transporter, permease protein, putative | NA | -2.25 |
| BA1654 | conserved hypothetical protein | NA | -2.24 |
| BA5390 | hydrolase, haloacid dehalogenase-like family | NA | -2.24 |
| BA0330 | polysaccharide deacetylase-like protein | NA | -2.24 |
| BA3324 | RNA polymerase sigma-70 factor, ECF subfamily | NA | -2.24 |
| BA2666 | hypothetical protein | NA | -2.23 |
| BA4572 | conserved hypothetical protein | NA | -2.22 |
| BA2061 | CBS domain protein | NA | -2.22 |
| BA5134 | transcriptional regulator, AsnC family | NA | -2.21 |
| BA2307 | protein kinase domain protein | NA | -2.21 |
| BA1430 | imidazoleglycerol phosphate synthase, cyclase subunit | hisF | -2.21 |
| BA4060 | acetyltransferase, GNAT family | NA | -2.21 |
| BA1279 | conserved hypothetical protein | NA | -2.21 |
| BA2239 | conserved hypothetical protein | NA | -2.20 |
| BA0783 | transcription antiterminator, LytR family | NA | -2.19 |
| BA0293 | phosphoribosylformylglycinamidine synthetase I | purQ | -2.18 |
| BA1322 | conserved hypothetical protein | NA | -2.18 |
| BA1501 | hypothetical protein | NA | -2.18 |
| BA3257 | transcriptional regulator, ArsR family | NA | -2.17 |
| BA0656 | oligopeptide ABC transporter, oligopeptide-binding protein | NA | -2.17 |
| BA2957 | hypothetical protein | NA | -2.17 |
| BA3858 | DNA-binding protein HU | hup-3 | -2.16 |
| BA0686 | DNA-binding protein | NA | -2.15 |
| BA3151 | hypothetical protein | NA | -2.15 |
| BA0658 | oligopeptide ABC transporter, permease protein | NA | -2.15 |
| BA4203 | EAL-domain protein | NA | -2.14 |
| BA5028 | sensor histidine kinase, putative | NA | -2.14 |
| BA4646 | preprotein translocase, YajC subunit | yajC | -2.14 |
| BA1663 | hypothetical protein | NA | -2.14 |
| BA3153 | response regulator | NA | -2.13 |
| BA0782 | hypothetical protein | NA | -2.13 |
| BA3486 | CAAX amino terminal protease family protein | NA | -2.12 |
| BA0366 | fatty acid desaturase | NA | -2.12 |
| BA5059 | hypothetical protein | NA | -2.12 |
| BA4446 | conserved hypothetical protein | NA | -2.12 |
| BA4901 | septation ring formation regulator | ezrA | -2.12 |
| BA2145 | nitrite reductase [NAD(P)H], small subunit | nirD | -2.12 |
| BA5550 | ATP synthase F1, delta subunit | atpH | -2.11 |
| BA0396 | prolyl-tRNA synthetase | proS-1 | -2.10 |
| BA4329 | conserved hypothetical protein | NA | -2.10 |
| BA1579 | conserved hypothetical protein | NA | -2.09 |
| BA1624 | lacX protein, putative | NA | -2.09 |
| BA1243 | membrane protein, putative | NA | -2.09 |
| BA2620 | hypothetical protein | NA | -2.08 |
| BA2955 | histidinol-phosphate aminotransferase | hisC-2 | -2.08 |
| BA3140 | conserved hypothetical protein | NA | -2.08 |
| BA1247 | hypothetical protein | NA | -2.08 |
| BA2134 | molybdopterin biosynthesis protein MoeB, putative | NA | -2.08 |
| BA5116 | conserved hypothetical protein | NA | -2.07 |
| BA5479 | glycine betaine transporter | opuD-2 | -2.07 |
| BA5193 | transcriptional regulator, DeoR family | NA | -2.07 |
| BA1095 | hypothetical protein | NA | -2.07 |
| BA5212 | conserved hypothetical protein | NA | -2.07 |
| BA4750 | D-alanyl-D-alanine carboxypeptidase family protein | NA | -2.06 |
| BA3737 | N-acetylmuramoyl-L-alanine amidase, family 2 | NA | -2.06 |
| BA1986 | conserved hypothetical protein | NA | -2.06 |
| BA1818 | N-acetylmuramoyl-L-alanine amidase, family 4 | NA | -2.05 |
| BA5458 | transposase, IS605 family, OrfA | NA | -2.05 |
| BA2079 | alanine racemase | dal-2 | -2.04 |
| BA2081 | acetyltransferase, GNAT family | NA | -2.04 |
| BA5696 | superoxide dismutase, Mn | sodA-2 | -2.04 |
| BA1683 | conserved domain protein | NA | -2.04 |
| BA5456 | hypothetical protein | NA | -2.04 |
| BA1549 | conserved hypothetical protein | NA | -2.03 |
| BA4167 | hypothetical protein | NA | -2.03 |
| BA4547 | ribosomal protein S20 | rpsT | -2.03 |
| BA0883 | polysaccharide biosynthesis protein CsaA | NA | -2.03 |
| BA1953 | hydrolase, alpha/beta fold family | NA | -2.03 |
| BA1687 | conserved hypothetical protein | NA | -2.03 |
| BA2908 | transcriptional regulator, GntR family, putative, authentic frameshift | NA | -2.03 |
| BA1817 | N-acetylmuramoyl-L-alanine amidase, family 3 | NA | -2.02 |
| BA0486 | hypothetical protein | NA | -2.02 |
| BA2958 | chorismate mutase/phospho-2-dehydro-3-deoxyheptonate aldolase | NA | -2.02 |
| BA1975 | DNA-binding response regulator | NA | -2.02 |
| BA1435 | CDP-diacylglycerol--serine O-phosphatidyltransferase, putative | NA | -2.02 |
| BA1982 | siderophore biosynthesis protein, putative | NA | -2.02 |
| BA0213 | 1-acyl-sn-glycerol-3-phosphate acyltransferase, putative | NA | -2.01 |
| BA5152 | UTP-glucose-1-phosphate uridylyltransferase | gtaB | -2.01 |
| BA1469 | hypothetical protein | NA | -2.01 |
| BA5210 | conserved hypothetical protein | NA | -2.01 |
| BA0602 | lipoprotein, putative | NA | -2.01 |
| BA4070 | prophage LambdaBa02, repressor protein | NA | -2.00 |
| BA4276 | conserved hypothetical protein TIGR00281 | NA | -2.00 |
| BA1720 | alanyl-tRNA synthetase domain protein | NA | -2.00 |
| BA5221 | ABC transporter, permease protein | NA | -2.00 |
| SpxA2DD positively regulated genes (after 45 minutes of SpxA2DD induction) | | | |
| BA4164 | hypothetical protein | NA | 2.00 |
| BA1639 | germination protein gerN | NA | 2.00 |
| BA4960 | conserved hypothetical protein TIGR00275 | NA | 2.00 |
| BA4345 | Na /H antiporter NhaC | nhaC-3 | 2.00 |
| BA0501 | PTS system, N-acetylglucosamine-specific IIBC component, putative | NA | 2.00 |
| BA5112 | menaquinone-specific isochorismate synthase | menF | 2.01 |
| BA5686 | transporter, AcrB/AcrD/AcrF family | NA | 2.01 |
| BA4056 | cell division protein FtsL | ftsL | 2.03 |
| BA1557 | conserved hypothetical protein | NA | 2.03 |
| BA3526 | arsenical pump family protein | NA | 2.03 |
| BA3905 | DNA mismatch repair protein MutS | mutS | 2.03 |
| BA3874 | hypothetical protein | NA | 2.04 |
| BA4610 | peptidase, U32 family | NA | 2.04 |
| BA1520 | isopentenyl-diphosphate delta-isomerase | NA | 2.05 |
| BA4780 | conserved hypothetical protein | NA | 2.05 |
| BA4905 | conserved hypothetical protein | NA | 2.05 |
| BA1823 | conserved hypothetical protein | NA | 2.05 |
| BA5204 | conserved hypothetical protein | NA | 2.05 |
| BA2360 | exonuclease, putative | NA | 2.06 |
| BA0623 | conserved hypothetical protein | NA | 2.06 |
| BA0847 | glutamate racemase | racE-1 | 2.06 |
| BA2172 | hypothetical protein | NA | 2.07 |
| BA3946 | riboflavin biosynthesis protein RibC | ribC | 2.07 |
| BA1088 | conserved hypothetical protein | NA | 2.07 |
| BA2918 | hypothetical protein | NA | 2.07 |
| BA3562 | hypothetical protein | NA | 2.08 |
| BA3904 | DNA mismatch repair protein MutL | mutL | 2.08 |
| BA1828 | GTP-binding protein | NA | 2.08 |
| BA1526 | glycerol-3-phosphate dehydrogenase (NAD(P) ) | gpsA | 2.09 |
| BA3525 | conserved hypothetical protein | NA | 2.10 |
| BA2163 | HD domain protein | NA | 2.10 |
| BA2009 | methyl-accepting chemotaxis protein | NA | 2.10 |
| BA0499 | glutaminase A | glsA-1 | 2.10 |
| BA3655 | oxidoreductase, Gfo/Idh/MocA family | NA | 2.10 |
| BA3891 | 1-phosphatidylinositol phosphodiesterase | NA | 2.11 |
| BA1141 | ATP-dependent nuclease, subunit B | addB | 2.11 |
| BA4737 | membrane protein, putative | NA | 2.11 |
| BA1775 | hypothetical protein | NA | 2.12 |
| BA3930 | stage III sporulation protein E | NA | 2.12 |
| BA1692 | conserved hypothetical protein | NA | 2.12 |
| BA5048 | conserved hypothetical protein TIGR00278 | NA | 2.13 |
| BA1554 | conserved hypothetical protein | NA | 2.13 |
| BA1506 | CAAX amino terminal protease family protein | NA | 2.14 |
| BA2358 | conserved hypothetical protein | NA | 2.14 |
| BA4849 | DNA polymerase III, alpha subunit | dnaE | 2.15 |
| BA5705 | guanosine monophosphate reductase | guaC | 2.15 |
| BA4325 | membrane protein, putative | NA | 2.15 |
| BA3947 | tRNA pseudouridine synthase B | truB | 2.16 |
| BA4168 | inositol monophosphatase family protein | NA | 2.16 |
| BA5647 | conserved hypothetical protein | NA | 2.17 |
| BA0577 | response regulator | NA | 2.17 |
| BA4738 | membrane protein, putative | NA | 2.17 |
| BA4739 | conserved hypothetical protein | NA | 2.17 |
| BA0194 | oligopeptide ABC transporter, oligopeptide-binding protein, putative | NA | 2.18 |
| BA1960 | aminoglycoside 6-adenylyltransferase, putative | NA | 2.18 |
| BA4936 | hypothetical protein | NA | 2.18 |
| BA1767 | fumarate hydratase, class II | fumC | 2.18 |
| BA5335 | carboxylesterase | estA | 2.19 |
| BA3993 | ATP-dependent DNA helicase RecG | recG | 2.19 |
| BA4988 | conserved hypothetical protein | NA | 2.19 |
| BA4233 | conserved domain protein | NA | 2.19 |
| BA3427 | conserved hypothetical protein | NA | 2.20 |
| BA0164 | 6-phosphogluconate dehydrogenase, decarboxylating | yqjI | 2.21 |
| BA0374 | conserved domain protein | NA | 2.21 |
| BXA0154 | transposase X | NA | 2.21 |
| BA0629 | amino acid permease family protein | NA | 2.22 |
| BA2792 | acetyltransferase, GNAT family | NA | 2.22 |
| BA5691 | response regulator LytR | NA | 2.22 |
| BA0544 | conserved hypothetical protein | NA | 2.23 |
| BA4860 | metallo-beta-lactamase family protein | NA | 2.24 |
| BA3735 | conserved hypothetical protein, authentic frameshift | NA | 2.24 |
| BA1825 | multidrug resistance protein, putative, authentic frameshift | NA | 2.24 |
| BA4590 | DNA-binding protein | NA | 2.24 |
| BA3615 | membrane protein, putative | NA | 2.25 |
| BA4822 | primosomal protein DnaI | dnaI | 2.26 |
| BA5692 | sensor histidine kinase LytS | NA | 2.27 |
| BA1040 | helicase, UvrD/Rep family | NA | 2.27 |
| BA5031 | conserved hypothetical protein | NA | 2.27 |
| BA0940 | hypothetical protein | NA | 2.27 |
| BA3666 | conserved hypothetical protein | NA | 2.27 |
| BA3465 | metallo-beta-lactamase family protein | NA | 2.28 |
| BA0867 | alpha-acetolactate decarboxylase | alsD | 2.29 |
| BA1231 | dTDP-4-dehydrorhamnose reductase | rfbD | 2.29 |
| BA0196 | oxidoreductase, aldo/keto reductase family | NA | 2.30 |
| BA2038 | NADH:flavin oxidoreductase / NADH oxidase family protein | NA | 2.30 |
| BA4217 | conserved hypothetical protein | NA | 2.31 |
| BA0197 | pyrroline-5-carboxylate reductase, putative | NA | 2.32 |
| BA2867 | hypothetical protein | NA | 2.32 |
| BA3602 | oxidoreductase, short-chain dehydrogenase/reductase family | NA | 2.32 |
| BA2011 | hypothetical protein | NA | 2.34 |
| BA0411 | transporter, EamA family | NA | 2.34 |
| BA5650 | ABC transporter, ATP-binding protein | NA | 2.35 |
| BA5387 | thioredoxin reductase | trxB | 2.35 |
| BA3974 | succinyl-CoA synthase, beta subunit | sucC | 2.36 |
| BA3708 | transcriptional regulator, CarD family | NA | 2.36 |
| BA1210 | conserved hypothetical protein | NA | 2.36 |
| BA1110 | Ser/Thr protein phosphatase family protein, authentic point mutation | NA | 2.37 |
| BA4945 | thioredoxin family protein | NA | 2.37 |
| BA1312 | DNA-binding response regulator | NA | 2.37 |
| BA2059 | CBS domain protein | NA | 2.38 |
| BA1616 | glycosyltransferase, authentic frameshift | NA | 2.38 |
| BA0088 | serine O-acetyltransferase | cysE | 2.38 |
| BA0784 | alcohol dehydrogenase, zinc-containing | NA | 2.38 |
| BA4541 | heat-inducible transcription repressor HrcA | hrcA | 2.38 |
| BA0800 | ABC transporter, permease protein, putative | NA | 2.38 |
| BA3672 | DNA polymerase III, epsilon subunit, putative | NA | 2.39 |
| BA1785 | conserved hypothetical protein | NA | 2.39 |
| BA5281 | conserved hypothetical protein | NA | 2.40 |
| BA2866 | MTA/SAH nucleosidase / phosphatase, putative | NA | 2.40 |
| BA0250 | holo-(acyl-carrier-protein) synthase | acpS | 2.40 |
| BA4925 | conserved domain protein | NA | 2.40 |
| BA5568 | sua5/yciO/yrdC/ywlC family protein | NA | 2.41 |
| BA5169 | hesB/yadR/yfhF family protein | NA | 2.42 |
| BA4059 | 2-dehydropantoate 2-reductase | panE | 2.42 |
| BA4628 | ATPase, AAA family | NA | 2.42 |
| BA0520 | conserved hypothetical protein | NA | 2.43 |
| BA3922 | zinc protease, insulinase family | NA | 2.44 |
| BA5424 | cold shock protein CspC | cspC | 2.44 |
| BA1406 | transcriptional regulator, MarR family | NA | 2.48 |
| BA0674 | multidrug resistance protein, putative | NA | 2.48 |
| BA4588 | glyoxalase family protein, authentic frameshift | NA | 2.50 |
| BA5488 | conserved domain protein | NA | 2.50 |
| BA2896 | transporter, putative | NA | 2.50 |
| BA2280 | glycine betaine/L-proline ABC transporter, permease protein, putative | NA | 2.51 |
| BA1434 | D-isomer specific 2-hydroxyacid dehydrogenase family protein | NA | 2.52 |
| BA4781 | sodium/hydrogen exchanger family protein | NA | 2.55 |
| BA0163 | gluconate permease | gntP-1 | 2.55 |
| BA3840 | site-specific recombinase, phage integrase family | NA | 2.55 |
| BA4861 | proline dipeptidase | pepQ-2 | 2.56 |
| BA3574 | bile acid transporter family protein | NA | 2.58 |
| BA0617 | iron compound ABC transporter, permease protein | NA | 2.58 |
| BA0375 | DNA topoisomerase III | topB-1 | 2.59 |
| BA4885 | conserved hypothetical protein | NA | 2.60 |
| BA4498 | membrane protein, putative | NA | 2.61 |
| BA5130 | phosphoglucose isomerase | pgi | 2.61 |
| BA4961 | drug resistance transporter, EmrB/QacA family | NA | 2.62 |
| BA5543 | sensory box/GGDEF family protein | NA | 2.62 |
| BA2107 | formate--tetrahydrofolate ligase | fhs | 2.63 |
| BA5648 | uracil-DNA glycosylase | ung | 2.63 |
| BA3698 | N-acetylmuramoyl-L-alanine amidase, putative | NA | 2.63 |
| BA2484 | conserved hypothetical protein | NA | 2.67 |
| BA5649 | ABC transporter, permease protein | NA | 2.70 |
| BA1884 | 2-dehydropantoate 2-reductase | panE | 2.70 |
| BA1505 | ATP-dependent DNA helicase RecQ | recQ-1 | 2.71 |
| BA1313 | sensor histidine kinase | NA | 2.71 |
| BA1510 | negative regulator of competence MecA, putative | NA | 2.72 |
| BA4431 | lipoate-protein ligase A, putative | NA | 2.72 |
| BA5032 | hypothetical protein | NA | 2.73 |
| BA2180 | TPR domain protein | NA | 2.73 |
| BA4320 | conserved hypothetical protein | NA | 2.74 |
| BA5110 | hydrolase, alpha/beta fold family | NA | 2.75 |
| BA3428 | gluconate kinase | gntK | 2.75 |
| BA4005 | polypeptide deformylase | deF-2 | 2.78 |
| BA4874 | 3-oxoacyl-(acyl-carrier-protein) reductase, putative | NA | 2.80 |
| BA3209 | hypothetical protein | NA | 2.80 |
| BA4218 | 5-methyltetrahydropteroyltriglutamate--homocysteine methyltransferase | metE | 2.81 |
| BA5076 | hypothetical protein | NA | 2.82 |
| BA3466 | conserved hypothetical protein | NA | 2.83 |
| BA2872 | degV family protein | NA | 2.85 |
| BA5111 | 2-succinyl-6-hydroxy-2,4-cyclohexadiene-1-carboxylic acid synthase/2-oxoglutarate decarboxylase | menD | 2.85 |
| BA4378 | conserved hypothetical protein | NA | 2.86 |
| BA4873 | alanine dehydrogenase | ald-2 | 2.87 |
| BA4611 | O-methyltransferase family protein | NA | 2.90 |
| BA3614 | rarD protein | NA | 2.92 |
| BA3694 | conserved hypothetical protein | NA | 2.92 |
| BA2647 | alcohol dehydrogenase, zinc-containing | NA | 2.92 |
| BA2288 | CBS domain protein | NA | 2.92 |
| BA3429 | gluconate permease | gntP-2 | 2.93 |
| BA4935 | hypothetical protein | NA | 2.93 |
| BA4324 | hydrolase, alpha/beta fold family, putative | NA | 2.96 |
| BA0624 | norQ protein, putative | NA | 2.96 |
| BA4603 | conserved hypothetical protein | NA | 2.97 |
| BA4007 | phosphopantothenoylcysteine decarboxylase/phosphopantothenate--cysteine ligase | coaBC | 2.98 |
| BA5331 | DNA-binding response regulator | NA | 2.99 |
| BA1951 | conserved hypothetical protein | NA | 3.00 |
| BA1558 | glycosyl transferase, group 1 family protein | NA | 3.01 |
| BA1962 | hypothetical protein | NA | 3.02 |
| BA1555 | dihydrodipicolinate reductase | dapB | 3.03 |
| BA4757 | excinuclease ABC, C subunit | uvrC | 3.03 |
| BA1556 | methylglyoxal synthase | mgsA | 3.04 |
| BA2289 | aldehyde dehydrogenase family protein | NA | 3.05 |
| BA0382 | ABC transporter, substrate-binding protein, putative | NA | 3.05 |
| BA1832 | acetyltransferase, GNAT family | NA | 3.05 |
| BA4934 | lipoprotein, putative | NA | 3.08 |
| BA4868 | acetyltransferase, GNAT family | NA | 3.10 |
| BA3923 | conserved hypothetical protein | NA | 3.12 |
| BA3208 | glyoxylase family protein | NA | 3.13 |
| BA4058 | conserved hypothetical protein | NA | 3.17 |
| BA2043 | conserved hypothetical protein | NA | 3.19 |
| BA4160 | conserved hypothetical protein | NA | 3.19 |
| BA1802 | sensor histidine kinase | NA | 3.20 |
| BA0745 | phospholipase, putative | NA | 3.22 |
| BA3538 | conserved hypothetical protein | NA | 3.23 |
| BA1560 | birA bifunctional protein | birA | 3.23 |
| BA1559 | polyA polymerase | pcnB | 3.29 |
| BA5651 | lipase/acylhydrolase, putative | NA | 3.32 |
| BA4430 | hypothetical protein | NA | 3.33 |
| BA4469 | sodium:dicarboxylate symporter family protein | NA | 3.33 |
| BA4499 | superoxide dismutase, Mn | sodA-1 | 3.42 |
| BA0554 | glycine betaine transporter | opuD-1 | 3.42 |
| BA2357 | hypothetical protein | NA | 3.46 |
| BA5137 | conserved hypothetical protein | NA | 3.49 |
| BA1225 | conserved hypothetical protein | NA | 3.49 |
| BA0618 | iron compound ABC transporter, ATP-binding protein | NA | 3.49 |
| BA1880 | transport protein, NRAMP family | NA | 3.50 |
| BA1830 | fosmidomycin resistance protein | fsR | 3.52 |
| BA2947 | sulfatase | NA | 3.53 |
| BA3868 | exodeoxyribonuclease III | exoA | 3.58 |
| BA4840 | conserved hypothetical protein | NA | 3.59 |
| BA3063 | yaiI/yqxD family protein | NA | 3.60 |
| BA2174 | conserved hypothetical protein | NA | 3.67 |
| BA0616 | iron compound ABC transporter, permease protein | NA | 3.67 |
| BA3438 | alcohol dehydrogenase, zinc-containing | NA | 3.69 |
| BA2117 | metallo-beta-lactamase family protein | NA | 3.72 |
| BA2119 | glutathione peroxidase | bsaA | 3.73 |
| BA3433 | glucose-6-phosphate 1-dehydrogenase | zwf | 3.74 |
| BA1111 | HD domain protein | NA | 3.83 |
| BA2053 | cytosolic long-chain acyl-CoA thioester hydrolase family protein | NA | 3.90 |
| BA3430 | transaldolase, putative | NA | 3.92 |
| BA3432 | transketolase | tkt-1 | 4.00 |
| BA4725 | xanthine/uracil permease family protein | NA | 4.02 |
| BA0521 | yfhP protein | NA | 4.03 |
| BA2179 | hypothetical protein | NA | 4.10 |
| BA3552 | N-acetyltransferase family protein, authentic frameshift | NA | 4.12 |
| BA1833 | conserved domain protein | NA | 4.17 |
| BA5561 | low molecular weight phosphotyrosine protein phosphatase family protein | NA | 4.29 |
| BA1881 | rhodanese-like domain protein | NA | 4.29 |
| BA5687 | peptide methionine sulfoxide reductase | msrA-2 | 4.31 |
| BA3431 | 6-phosphogluconate dehydrogenase family protein | NA | 4.34 |
| BA2279 | glycine betaine/L-proline ABC transporter, ATP-binding protein | proV-1 | 4.38 |
| BA0837 | lipoprotein, putative | NA | 4.41 |
| BA3545 | phosphoglycerate mutase, putative | NA | 4.43 |
| BA0848 | conserved hypothetical protein | NA | 4.47 |
| BA1209 | protozoan/cyanobacterial globin family protein | NA | 4.48 |
| BA0535 | potassium channel protein, putative | NA | 4.58 |
| BA4992 | permease, putative | NA | 4.68 |
| BA0653 | sulfate permease family protein | NA | 4.85 |
| BA0251 | lipoprotein, putative | NA | 4.86 |
| BA4319 | oxidoreductase, aldo/keto reductase family | NA | 4.90 |
| BA1831 | cysteine synthase A | cysK-2 | 4.91 |
| BA1263 | pyridine nucleotide-disulfide oxidoreductase, class I | NA | 5.10 |
| BA1359 | exsB protein | NA | 5.25 |
| BA3020 | major facilitator family transporter | NA | 5.26 |
| BA0774 | pyridine nucleotide-disulfide oxidoreductase, class I | NA | 5.30 |
| BA3543 | transcriptional regulator, LysR family | NA | 5.32 |
| BA4724 | germination protein GerE | gerE | 5.37 |
| BA0838 | NAD(P)H dehydrogenase, quinone family | NA | 5.44 |
| BA3607 | sodium/pantothenate symporter, putative | NA | 5.50 |
| BA1480 | hypothetical protein | NA | 5.71 |
| BA3515 | alcohol dehydrogenase, zinc-containing, authentic point mutation | NA | 5.79 |
| BA5209 | 5-nucleotidase family protein, truncation | NA | 5.83 |
| BA0591 | glycerophosphoryl diester phosphodiesterase family protein | NA | 6.11 |
| BA0533 | ABC transporter, permease protein, putative | NA | 6.26 |
| BA4372 | conserved hypothetical protein | NA | 6.44 |
| BA5002 | conserved hypothetical protein | NA | 6.51 |
| BA1858 | major facilitator family transporter | NA | 6.53 |
| BA1196 | MATE efflux family protein | NA | 6.73 |
| BA0787 | major facilitator family transporter | NA | 6.82 |
| BA0532 | ABC transporter, ATP-binding protein | NA | 6.86 |
| BA0862 | conserved hypothetical protein | NA | 7.13 |
| BA5533 | NADH dehydrogenase I, M subunit | nuoM | 7.25 |
| BA5010 | bacterial transferase family protein | NA | 7.48 |
| BA1208 | conserved hypothetical protein | NA | 7.60 |
| BA3473 | AMP-binding protein | NA | 8.57 |
| BA3078 | conserved hypothetical protein | NA | 8.99 |
| BA4736 | DNA-binding response regulator | NA | 9.52 |
| BA0534 | ABC transporter, permease protein, putative | NA | 9.57 |
| BA0585 | DNA-binding response regulator | NA | 9.75 |
| BA3456 | conserved hypothetical protein | NA | 9.95 |
| BA5208 | conserved hypothetical protein | NA | 11.46 |
| BA0963 | dihydroxyacetone kinase family protein | NA | 13.46 |
| BA4923 | oxidoreductase, Gfo/Idh/MocA family | NA | 15.70 |
| BA3849 | alcohol dehydrogenase, iron-containing, authentic frameshift | NA | 18.24 |
| BA3077 | conserved hypothetical protein | NA | 21.05 |
| BA3873 | membrane protein, putative | NA | 21.73 |
| BA2057 | oxidoreductase, putative | NA | 25.51 |
| BA1228 | glucose-1-phosphate thymidylyltransferase, putative | NA | 41.63 |
| BA0975 | HD domain protein | NA | 111.06 |
