# Supplementary material for: Transcriptomic and phenotypic analysis of paralogous spx gene function in Bacillus anthracis Sterne
Source: Microbiologyopen. 2013 Jul 22;2(4):695–714. doi: 10.1002/mbo3.109 (PMC3831629; doi:10.1002/mbo3.109)
Supplement: Supplementary file 5 — Table S3. SpxA1DD-activated genes. Fifteen minutes after induction. .threefold increase. [file mbo30002-0695-SD5.pdf]

Table S3: SpxA1DD-activated genes. 15 min after induction. ≥3-fold increase

SpxA1 15 min ≥3-fold upregulated genes: Cell Surface and Virulence

Locus	GeneN	GeneS	15 min	Fold-change
BA4325	membrane protein, putative	NA	1.62	3.08
BA0381	ABC transporter, permease protein, putative	NA	1.69	3.23
BA3526	arsenical pump family protein	NA	1.74	3.33
BA1302	transporter, putative	NA	1.74	3.35
BA4216	drug resistance transporter, EmrB/QacA family	NA	1.81	3.51
BA5411	ABC transporter, ATP-binding/permease protein	NA	1.84	3.57
BA3740	ABC transporter, ATP-binding/permease protein	NA	2.02	4.05
BA0411	transporter, EamA family	NA	2.02	4.05
BA4961	drug resistance transporter, EmrB/QacA family	NA	2.03	4.08
BA2896	transporter, putative	NA	2.18	4.54
BA3741	ABC transporter, ATP-binding/permease protein	NA	2.37	5.16
BA0528	ABC transporter, ATP-binding/permease protein	NA	2.38	5.21
BA5650	ABC transporter, ATP-binding protein	NA	2.39	5.24
BA0885	S-layer protein Sap	sap	2.41	5.31
BA1196	MATE efflux family protein	NA	2.45	5.47
BA0382	ABC transporter, substrate-binding protein, putative	NA	2.72	6.57
BA4498	membrane protein, putative	NA	2.90	7.47
BA3416	membrane protein, putative	NA	2.90	7.48
BA3607	sodium/pantothenate symporter, putative	NA	2.97	7.82
BA0535	potassium channel protein, putative	NA	3.13	8.73
BA4737	membrane protein, putative	NA	3.13	8.75
BA0532	ABC transporter, ATP-binding protein	NA	3.21	9.28
BA0533	ABC transporter, permease protein, putative	NA	3.27	9.62
BA2279	glycine betaine/L-proline ABC transporter, ATP-binding pr	proV-1	3.34	10.16
BA3873	membrane protein, putative	NA	3.37	10.35
BA0534	ABC transporter, permease protein, putative	NA	4.20	18.33
BA0521	yfhP protein	NA	1.61	3.04
BA1830	fosmidomycin resistance protein	fsR	1.86	3.63
BA5649	ABC transporter, permease protein	NA	1.89	3.70
BA3179	TspO/MBR family protein	NA	1.93	3.81
BA5645	xanthine/uracil permease family protein	NA	1.93	3.81
BA0415	dedA family protein	NA	1.93	3.82
BA4577	hydrolase, alpha/beta fold family	NA	2.09	4.24
BA1825	multidrug resistance protein, putative, authentic framesh	NA	2.09	4.26
BA0674	multidrug resistance protein, putative	NA	2.16	4.48
BA0244	major facilitator family transporter	NA	2.17	4.49
BA0251	lipoprotein, putative	NA	2.18	4.55
BA2933	lipoprotein, putative	NA	1.63	3.11
BA0837	lipoprotein, putative	NA	3.65	12.58
BA1038	drug resistance transporter, EmrB/QacA family	NA	2.28	4.85
BA3429	gluconate permease	gntP-2	2.43	5.39
BA2280	glycine betaine/L-proline ABC transporter, permease prot	NA	2.46	5.49
BA1037	major facilitator family transporter	NA	2.77	6.83
BA1960	aminoglycoside 6-adenylyltransferase, putative	NA	2.86	7.26
BA1881	rhodanese-like domain protein	NA	1.85	3.60
BA0554	glycine betaine transporter	opuD-1	2.98	7.88
BA0787	major facilitator family transporter	NA	3.17	9.01
BA1858	major facilitator family transporter	NA	3.32	10.00
BA1880	transport protein, NRAMP family	NA	3.41	10.60
BA3020	major facilitator family transporter	NA	4.36	20.47
BA4725	xanthine/uracil permease family protein	NA	3.54	11.61
BA3614	rarD protein	NA	3.59	12.03
BA5048	conserved hypothetical protein TIGR00278	NA	1.71	3.27
BA2357	hypothetical protein	NA	2.69	6.47

SpxA1 15 min ≥3-fold upregulated genes: Unknown

Locus	GeneN	GeneS	15 min	Fold-change
BA4936	hypothetical protein	NA	1.59	3.01
BA4867	conserved domain protein	NA	1.67	3.19
BA1036	conserved hypothetical protein	NA	1.72	3.30
BA5255	conserved hypothetical protein TIGR00106	NA	1.74	3.35
BA0848	conserved hypothetical protein	NA	1.75	3.36
BA3069	conserved hypothetical protein	NA	1.82	3.53
BA0380	conserved hypothetical protein	NA	1.85	3.60
BA1962	hypothetical protein	NA	1.89	3.70
BA1959	hypothetical protein	NA	1.92	3.79
BA1785	conserved hypothetical protein	NA	1.92	3.79
BA3694	conserved hypothetical protein	NA	1.97	3.91
BA2011	hypothetical protein	NA	2.01	4.03
BA5488	conserved domain protein	NA	2.03	4.10
BA3874	hypothetical protein	NA	2.04	4.10
BA5491	conserved hypothetical protein, authentic frar	NA	2.06	4.16
BA2867	hypothetical protein	NA	2.41	5.32
BA1775	hypothetical protein	NA	2.50	5.64
BA2546	conserved hypothetical protein	NA	2.70	6.52
BA3798	hypothetical protein	NA	2.76	6.79
BA1262	hypothetical protein	NA	2.98	7.89
BA3618	hypothetical protein	NA	3.07	8.41
BA0862	conserved hypothetical protein	NA	1.64	3.12
BA4935	hypothetical protein	NA	1.64	3.12

SpxA1 15 min ≥3-fold upregulated genes: Protein synthesis & degradation

Locus	GeneN	GeneS	15 min	Fold-change
BA3947	tRNA pseudouridine synthase B	truB	1.64	3.13
BA4861	proline dipeptidase	pepQ-2	1.72	3.30
BA4324	hydrolase, alpha/beta fold family, putative	NA	1.84	3.59
BA1558	glycosyl transferase, group 1 family protein	NA	2.09	4.26
BA3684	alanyl-tRNA synthetase family protein	NA	2.17	4.50
BA1110	Ser/Thr protein phosphatase family protein, authentic point mutati	NA	2.56	5.88
BA4431	lipoate-protein ligase A, putative	NA	1.82	3.54
BA0493	acetylornitine deacetylase, putative	NA	2.06	4.17
BA3877	hydrolase, alpha/beta fold family	NA	3.87	14.57
BA3923	conserved hypothetical protein	NA	2.23	4.68

SpxA1 15 min ≥3-fold upregulated genes: Oxidative Stress

Locus	GeneN	GeneS	15 min	Fold-change
BA5387	thioredoxin reductase	trxB	1.63	3.09
BA1515	pyridine nucleotide-disulfide oxidoreductase family	NA	1.64	3.11
BA4923	oxidoreductase, Gfo/Idh/MocA family	NA	1.71	3.27
BA2288	CBS domain protein	NA	1.91	3.76
BA3655	oxidoreductase, Gfo/Idh/MocA family	NA	2.02	4.07
BA0196	oxidoreductase, aldo/keto reductase family	NA	2.05	4.13
BA4945	thioredoxin family protein	NA	2.06	4.18
BA2119	glutathione peroxidase	bsaA	2.17	4.49
BA3602	oxidoreductase, short-chain dehydrogenase/reduct	NA	2.21	4.63
BA0197	pyrroline-5-carboxylate reductase, putative	NA	2.28	4.86
BA2059	CBS domain protein	NA	2.51	5.69
BA4319	oxidoreductase, aldo/keto reductase family	NA	2.56	5.90
BA1263	pyridine nucleotide-disulfide oxidoreductase, class I	NA	3.70	12.98
BA1209	protozoan/cyanobacterial globin family protein	NA	3.43	10.79
BA1200	conserved hypothetical protein	NA	1.93	3.81
BA1208	conserved hypothetical protein	NA	3.03	8.14
BA1951	conserved hypothetical protein	NA	5.03	32.58
BA4058	conserved hypothetical protein	NA	1.74	3.35
BA4059	2-dehydropantoate 2-reductase	panE	1.63	3.09
BA1555	dihydrodipicolinate reductase	dapB	1.67	3.19
BA4318	lolS protein	lolS	1.69	3.22
BA2016	bacterial luciferase family protein	NA	2.00	4.00
BA1884	2-dehydropantoate 2-reductase	panE	2.16	4.46
BA5687	peptide methionine sulfoxide reductase	msrA-2	2.82	7.05
BA4874	3-oxoacyl-(acyl-carrier-protein) reductase, putative	NA	2.82	7.08
BA2172	hypothetical protein	NA	1.68	3.21
BA4160	conserved hypothetical protein	NA	2.50	5.67

SpxA1 15 min ≥3-fold upregulated genes: Metabolism

Locus	GeneN	GeneS	15 min	Fold-change
BA0499	glutaminase A	glsA-1	1.59	3.01
BA1296	aldehyde dehydrogenase	ywdH	1.99	3.98
BA3428	gluconate kinase	gntK	2.08	4.23
BA2289	aldehyde dehydrogenase family protein	NA	2.13	4.36
BA3849	alcohol dehydrogenase, iron-containing, authentic fra	NA	2.14	4.42
BA0675	alcohol dehydrogenase, zinc-containing	NA	2.15	4.45
BA2038	NADH:flavin oxidoreductase / NADH oxidase family p	NA	2.16	4.46
BA1556	methylglyoxal synthase	mgsA	2.23	4.71
BA3430	transaldolase, putative	NA	2.29	4.88
BA3438	alcohol dehydrogenase, zinc-containing	NA	2.43	5.39
BA0838	NAD(P)H dehydrogenase, quinone family	NA	2.66	6.32
BA3433	glucose-6-phosphate 1-dehydrogenase	zwf	2.72	6.60
BA3432	transketolase	tkt-1	3.03	8.19
BA3704	glyoxylase family protein	NA	2.36	5.14
BA1520	isopentenyl-diphosphate delta-isomerase	NA	1.59	3.02
BA3876	phosphoglycerate mutase family protein, putative	NA	1.62	3.07
BA0624	norQ protein, putative	NA	1.65	3.14
BA5335	carboxylesterase	estA	1.75	3.37
BA1496	resC protein	resC	1.76	3.40
BA0596	nicotinate phosphoribosyltransferase, putative	NA	1.81	3.51
BA2647	alcohol dehydrogenase, zinc-containing	NA	1.82	3.54
BA4588	glyoxalase family protein, authentic frameshift	NA	1.84	3.57
BA5043	mutT/nudix family protein	NA	1.85	3.61
BA2418	protoporphyrinogen oxidase	hemY-2	1.88	3.68
BA5648	uracil-DNA glycosylase	ung	1.94	3.83
BA0250	holo-(acyl-carrier-protein) synthase	acpS	1.95	3.86
BA5209	5-nucleotidase family protein, truncation	NA	1.98	3.94
BA2947	sulfatase	NA	2.13	4.39
BA3703	phospholipase/carboxylesterase family protein	NA	2.25	4.76
BA1767	fumarate hydratase, class II	fumC	2.30	4.91
BA5135	D-isomer specific 2-hydroxyacid dehydrogenase famil	NA	2.27	4.81
BA2117	metallo-beta-lactamase family protein	NA	2.30	4.92
BA4359	pyrroline-5-carboxylate reductase	proC-3	2.32	5.00
BA2053	cytosolic long-chain acyl-CoA thioester hydrolase fam	NA	2.35	5.11
BA3431	6-phosphogluconate dehydrogenase family protein	NA	2.36	5.13
BA1831	cysteine synthase A	cysK-2	2.42	5.36
BA1434	D-isomer specific 2-hydroxyacid dehydrogenase famil	NA	2.50	5.65
BA5651	lipase/acylhydrolase, putative	NA	2.56	5.90
BA3545	phosphoglycerate mutase, putative	NA	2.90	7.49
BA3392	hydrolase, haloacid dehalogenase-like family	NA	2.99	7.96
BA3620	mrp protein	NA	3.85	14.46
BA1242	conserved hypothetical protein	NA	1.63	3.10
BA4988	conserved hypothetical protein	NA	1.74	3.35
BA5281	conserved hypothetical protein	NA	1.89	3.71
BA5031	conserved hypothetical protein	NA	1.90	3.74
BA1210	conserved hypothetical protein	NA	1.95	3.87
BA4946	conserved hypothetical protein	NA	2.29	4.90

BA5032	hypothetical protein	NA	2.57	5.95
BA5482	hypothetical protein	NA	3.36	10.25
BA5708	conserved hypothetical protein TIGR00246	NA	2.32	4.99

SpxA1 15 min ≥3-fold upregulated genes: Gene Regulation

Locus	GeneN	GeneS	15 min	Fold-change
BA1111	HD domain protein	NA	1.61	3.06
BA1510	negative regulator of competence MecA, puta	NA	1.66	3.17
BA5105	sensor histidine kinase	NA	1.66	3.17
BA0079	phosphotransferase domain protein	NA	1.69	3.23
BA5713	YycH protein	NA	1.74	3.34
BA0585	DNA-binding response regulator	NA	1.79	3.46
BA5714	sensory box histidine kinase YycG	yycG	1.81	3.51
BA5148	comA operon protein, putative	NA	1.88	3.68
BA0080	negative regulator of genetic competence Clp(	NA	1.95	3.86
BA0975	HD domain protein	NA	2.02	4.07
BA3708	transcriptional regulator, CarD family	NA	2.05	4.13
BA0077	transcriptional regulator CtsR	ctsR	2.07	4.20
BA4541	heat-inducible transcription repressor HrcA	hrcA	2.10	4.28
BA4736	DNA-binding response regulator	NA	2.87	7.29
BA3543	transcriptional regulator, LysR family	NA	3.37	10.31
BA1119	DNA-binding response regulator	NA	3.70	12.97
BA1118	sensor histidine kinase	NA	4.17	17.99
BA3907	conserved hypothetical protein	NA	1.73	3.33
BA4543	conserved hypothetical protein	NA	1.72	3.30
BA1560	birA bifunctional protein	birA	2.31	4.95
BA5424	cold shock protein CspC	cspC	2.36	5.12
BA0938	conserved hypothetical protein	NA	1.65	3.13
BA4739	conserved hypothetical protein	NA	2.38	5.22

SpxA1 15 min ≥3-fold upregulated genes: DNA Replication and Repair

Locus	GeneN	GeneS	15 min	Fold-change
BA4628	ATPase, AAA family	NA	1.85	3.62
BA5487	helicase, putative	NA	1.86	3.63
BA0081	DNA repair protein RadA	radA	2.05	4.15
BA1505	ATP-dependent DNA helicase RecQ	recQ-1	2.11	4.31
BA0037	deoxyribonuclease, TatD family	NA	2.12	4.34
BA4757	excinuclease ABC, C subunit	uvrC	2.29	4.91
BA3335	conserved hypothetical protein	NA	2.00	3.99
BA4868	acetyltransferase, GNAT family	NA	2.22	4.67
BA1832	acetyltransferase, GNAT family	NA	2.46	5.52
BA1141	ATP-dependent nuclease, subunit B	addB	1.61	3.05
BA0078	conserved hypothetical protein	NA	1.82	3.52
BA2174	conserved hypothetical protein	NA	1.61	3.05
BA1833	conserved domain protein	NA	2.36	5.12

SpxA1 15 min ≥3-fold upregulated genes: Division and Sporulation

Locus	GeneN	GeneS	15 min	Fold-change
BA4724	germination protein GerE	gerE	2.09	4.26

SpxA1 15 min ≥3-fold upregulated genes: Unknown

Locus	GeneN	GeneS	15 min	Fold-change
BA4936	hypothetical protein	NA	1.59	3.01
BA4867	conserved domain protein	NA	1.67	3.19
BA1036	conserved hypothetical protein	NA	1.72	3.30
BA5255	conserved hypothetical protein TIGR00106	NA	1.74	3.35
BA0848	conserved hypothetical protein	NA	1.75	3.36
BA3069	conserved hypothetical protein	NA	1.82	3.53
BA0380	conserved hypothetical protein	NA	1.85	3.60
BA1962	hypothetical protein	NA	1.89	3.70
BA1959	hypothetical protein	NA	1.92	3.79
BA1785	conserved hypothetical protein	NA	1.92	3.79
BA3694	conserved hypothetical protein	NA	1.97	3.91
BA2011	hypothetical protein	NA	2.01	4.03
BA5488	conserved domain protein	NA	2.03	4.10
BA3874	hypothetical protein	NA	2.04	4.10
BA5491	conserved hypothetical protein, authentic frar	NA	2.06	4.16
BA2867	hypothetical protein	NA	2.41	5.32
BA1775	hypothetical protein	NA	2.50	5.64
BA2546	conserved hypothetical protein	NA	2.70	6.52
BA3798	hypothetical protein	NA	2.76	6.79
BA1262	hypothetical protein	NA	2.98	7.89
BA3618	hypothetical protein	NA	3.07	8.41
BA0862	conserved hypothetical protein	NA	1.64	3.12
BA4935	hypothetical protein	NA	1.64	3.12
