# Supplementary material for: Transcriptomic and phenotypic analysis of paralogous spx gene function in Bacillus anthracis Sterne
Source: Microbiologyopen. 2013 Jul 22;2(4):695–714. doi: 10.1002/mbo3.109 (PMC3831629; doi:10.1002/mbo3.109)
Supplement: Supplementary file 6 — Table S4. SpxA2DD-activated. Fifteen minutes after induction. .threefold increase. [file mbo30002-0695-SD6.pdf]

Table S4: SpxA2DD-activated. 15 min. after induction. ≥3-fold increase

SpxA2 upreguated genes ≥3-fold: Cell Surface & Virulence

Locus	GeneN	GeneS	15_min	fold-change
BA0847	glutamate racemase	racE-1	3.99	15.90
BA0533	ABC transporter, permease protein, putative	NA	3.43	10.75
BA0554	glycine betaine transporter	opuD-1	2.99	7.96
BA4498	membrane protein, putative	NA	2.96	7.77
BA2279	glycine betaine/L-proline ABC transporter, ATP-binding protein	proV-1	2.57	5.96
BA0534	ABC transporter, permease protein, putative	NA	2.61	6.13
BA0532	ABC transporter, ATP-binding protein	NA	2.45	5.47
BA0535	potassium channel protein, putative	NA	2.46	5.49
BA4738	membrane protein, putative	NA	2.00	3.99
BA3873	membrane protein, putative	NA	2.05	4.15
BA4914	lipoprotein, putative	NA	2.06	4.18
BA4737	membrane protein, putative	NA	1.89	3.72
BA1196	MATE efflux family protein	NA	1.93	3.80
BA3607	sodium/pantothenate symporter, putative	NA	1.94	3.85
BA0382	ABC transporter, substrate-binding protein, putative	NA	1.99	3.97
BA0800	ABC transporter, permease protein, putative	NA	1.99	3.98
BA2896	transporter, putative	NA	1.81	3.50
BA0235	oligopeptide ABC transporter, ATP-binding protein	NA	1.86	3.63
BA0618	iron compound ABC transporter, ATP-binding protein	NA	1.59	3.00
BA0411	transporter, EamA family	NA	1.62	3.07
BA5201	membrane protein, putative	NA	1.67	3.18
BA3518	drug resistance transporter, Bcr/CflA family	NA	1.74	3.33
BA3020	major facilitator family transporter	NA	2.94	7.70
BA1858	major facilitator family transporter	NA	3.00	7.97
BA0787	major facilitator family transporter	NA	2.39	5.25
BA4725	xanthine/uracil permease family protein	NA	2.47	5.53
BA4992	permease, putative	NA	1.61	3.05
BA1880	transport protein, NRAMP family	NA	2.68	6.41
BA0674	multidrug resistance protein, putative	NA	2.33	5.05
BA0975	HD domain protein	NA	2.19	4.55
BA0616	iron compound ABC transporter, permease protein	NA	2.15	4.43
BA2280	glycine betaine/L-proline ABC transporter, permease protein, pu	NA	2.15	4.44
BA1830	fosmidomycin resistance protein	fsR	2.04	4.11
BA5649	ABC transporter, permease protein	NA	1.94	3.84
BA1825	multidrug resistance protein, putative, authentic frameshift	NA	1.77	3.41
BA5048	conserved hypothetical protein TIGR00278	NA	2.27	4.84
BA2059	CBS domain protein	NA	1.68	3.20
BA0521	yfhP protein	NA	1.61	3.05
BA1962	hypothetical protein	NA	1.83	3.56
BA1960	aminoglycoside 6-adenylyltransferase, putative	NA	2.61	6.10
BA1881	rhodanese-like domain protein	NA	1.76	3.38

Locus	GeneN	GeneS	15_min	fold-change
BA4164	hypothetical protein	NA	4.14	17.64
BA3538	conserved hypothetical protein	NA	3.04	8.21
BA3798	hypothetical protein	NA	3.00	7.98
BA3077	conserved hypothetical protein	NA	2.82	7.06
BA4925	conserved domain protein	NA	2.39	5.23
BA2357	hypothetical protein	NA	2.06	4.18
	SpxA2 upreguated genes ≥3-fold: Unkown			
BA1480	hypothetical protein	NA	2.18	4.54
BA3735	conserved hypothetical protein, authe	NA	1.89	3.69
BA1775	hypothetical protein	NA	1.94	3.85
BA1262	hypothetical protein	NA	1.96	3.89
BA0527	conserved hypothetical protein	NA	1.78	3.44
BA5488	conserved domain protein	NA	1.79	3.45
BA2546	conserved hypothetical protein	NA	1.81	3.51
BA1833	conserved domain protein	NA	1.82	3.53
BA0848	conserved hypothetical protein	NA	1.68	3.20
BA4312	conserved hypothetical protein	NA	1.69	3.23
BA5137	conserved hypothetical protein	NA	1.69	3.24
BA4935	hypothetical protein	NA	1.71	3.26
BA2011	hypothetical protein	NA	1.72	3.30
BA1959	hypothetical protein	NA	1.76	3.40
BA3209	hypothetical protein	NA	1.77	3.42
BA5491	conserved hypothetical protein, authe	NA	1.75	3.36

Protein synthesis & degradation

Locus	GeneN	GeneS	15_min	fold-change
BA4324	hydrolase, alpha/beta fold family, putative	NA	2.02	4.05
BA5561	low molecular weight phosphotyrosine protei	NA	1.98	3.93
BA4431	lipoate-protein ligase A, putative	NA	1.80	3.49
BA5208	conserved hypothetical protein	NA	2.75	6.72
BA5002	conserved hypothetical protein	NA	2.17	4.49
BA1225	conserved hypothetical protein	NA	2.29	4.88
BA5238	PAP2 family protein	NA	1.67	3.19
BA3923	conserved hypothetical protein	NA	1.59	3.01
BA2174	conserved hypothetical protein	NA	1.68	3.21
BA5169	hesB/yadR/yfhF family protein	NA	1.60	3.04
BA5568	sua5/yciO/yrdC/ywlC family protein	NA	1.60	3.04

SpxA2 upreguated genes ≥3-fold: Oxidative Stress

Locus	GeneN	GeneS	15_min	fold-change
BA0774	pyridine nucleotide-disulfide oxidoreductase, class I	NA	3.67	12.76
BA3456	conserved hypothetical protein	NA	3.51	11.36
BA1208	conserved hypothetical protein	NA	2.80	6.95
BA4923	oxidoreductase, Gfo/Idh/MocA family	NA	2.80	6.97
BA1263	pyridine nucleotide-disulfide oxidoreductase, class I	NA	2.56	5.90
BA5387	thioredoxin reductase	trxB	2.12	4.34
BA1831	cysteine synthase A	cysK-2	2.01	4.02
BA2038	NADH:flavin oxidoreductase / NADH oxidase family protein	NA	1.62	3.07
BA5687	peptide methionine sulfoxide reductase	msrA-2	2.43	5.40
BA4874	3-oxoacyl-(acyl-carrier-protein) reductase, putative	NA	2.21	4.63
BA3707	NADH-dependent flavin oxidoreductase, Oye family	NA	2.07	4.19
BA0675	alcohol dehydrogenase, zinc-containing	NA	1.96	3.89
BA2057	oxidoreductase, putative	NA	1.94	3.84
BA4319	oxidoreductase, aldo/keto reductase family	NA	1.86	3.63
BA0196	oxidoreductase, aldo/keto reductase family	NA	1.72	3.28
BA4945	thioredoxin family protein	NA	1.72	3.29
BA2119	glutathione peroxidase	bsaA	1.67	3.19
BA0544	conserved hypothetical protein	NA	1.73	3.33
BA4058	conserved hypothetical protein	NA	1.76	3.39
BA2119	glutathione peroxidase	bsaA	1.67	3.19
BA4160	conserved hypothetical protein	NA	2.00	3.99
BA3666	conserved hypothetical protein	NA	1.78	3.43
BA2987	conserved hypothetical protein	NA	2.81	7.01
BA3473	AMP-binding protein	NA	2.69	6.44

SpxA2 upreguated genes ≥3-fold: Metabolism

Locus	GeneN	GeneS	15_min	fold-change
BA3432	transketolase	tkt-1	2.41	5.32
BA0784	alcohol dehydrogenase, zinc-containing	NA	2.14	4.41
BA1767	fumarate hydratase, class II	fumC	2.16	4.48
BA5209	5-nucleotidase family protein, truncation	NA	2.24	4.74
BA2289	aldehyde dehydrogenase family protein	NA	2.27	4.83
BA1434	D-isomer specific 2-hydroxyacid dehydrogenase family protein	NA	2.33	5.03
BA0838	NAD(P)H dehydrogenase, quinone family	NA	2.47	5.53
BA3438	alcohol dehydrogenase, zinc-containing	NA	2.78	6.85
BA2647	alcohol dehydrogenase, zinc-containing	NA	3.11	8.62
BA3515	alcohol dehydrogenase, zinc-containing, authentic point mutation	NA	4.77	27.30
BA5675	cytosolic long-chain acyl-CoA thioester hydrolase family protein	NA	2.66	6.32
BA1111	HD domain protein	NA	2.09	4.25
BA3433	glucose-6-phosphate 1-dehydrogenase	zwf	2.12	4.36
BA2053	cytosolic long-chain acyl-CoA thioester hydrolase family protein	NA	2.15	4.44
BA3208	glyoxylase family protein	NA	2.06	4.18
BA4588	glyoxalase family protein, authentic frameshift	NA	2.02	4.05
BA4218	5-methyltetrahydropteroyltriglutamate--homocysteine methyltrans	metE	1.97	3.92
BA3430	transaldolase, putative	NA	1.92	3.78
BA3946	riboflavin biosynthesis protein RibC	ribC	1.84	3.57
BA5205	lipoic acid synthetase	lipA	1.83	3.55
BA0197	pyrroline-5-carboxylate reductase, putative	NA	1.84	3.59
BA3545	phosphoglycerate mutase, putative	NA	1.82	3.54
BA4168	inositol monophosphatase family protein	NA	1.83	3.55
BA3431	6-phosphogluconate dehydrogenase family protein	NA	1.75	3.36
BA5281	conserved hypothetical protein	NA	1.88	3.68
BA2484	conserved hypothetical protein	NA	1.94	3.84
BA1210	conserved hypothetical protein	NA	1.72	3.28
BA5651	lipase/acylhydrolase, putative	NA	1.70	3.26
BA5533	NADH dehydrogenase I, M subunit	nuoM	1.71	3.27
BA4861	proline dipeptidase	pepQ-2	1.64	3.11
BA1836	polysaccharide deacetylase, putative	NA	1.63	3.10
BA5482	hypothetical protein	NA	1.81	3.50
BA5031	conserved hypothetical protein	NA	1.80	3.47
BA5032	hypothetical protein	NA	2.20	4.59
BA0745	phospholipase, putative	NA	1.60	3.04
BA2117	metallo-beta-lactamase family protein	NA	1.85	3.61

SpxA2 upreguated genes ≥3-fold: Gene regulation

Locus	GeneN	GeneS	15_min	fold-change
BA5331	DNA-binding response regulator	NA	4.03	16.38
BA3543	transcriptional regulator, LysR family	NA	3.69	12.95
BA1110	Ser/Thr protein phosphatase family protein, authentic poin	NA	2.03	4.10
BA1510	negative regulator of competence MecA, putative	NA	2.01	4.04
BA4736	DNA-binding response regulator	NA	2.20	4.58
BA5543	sensory box/GGDEF family protein	NA	2.78	6.88
BA4541	heat-inducible transcription repressor HrcA	hrcA	1.99	3.98
BA3261	DNA-binding response regulator	NA	1.95	3.85
BA0585	DNA-binding response regulator	NA	1.79	3.46
BA3220	transcriptional regulator, MarR family	NA	1.79	3.45
BA4739	conserved hypothetical protein	NA	1.65	3.14

SpxA2 upreguated genes ≥3-fold: DNA replication & repair

Locus	GeneN	GeneS	15_min	fold-change
BA1040	helicase, UvrD/Rep family	NA	3.50	11.30
BA1505	ATP-dependent DNA helicase RecQ	recQ-1	1.60	3.03
BA3840	site-specific recombinase, phage integrase family	NA	4.05	16.57
BA4757	excinuclease ABC, C subunit	uvrC	2.11	4.32
BA3868	exodeoxyribonuclease III	exoA	1.96	3.89
BA3672	DNA polymerase III, epsilon subunit, putative	NA	1.76	3.39
BA1141	ATP-dependent nuclease, subunit B	addB	1.61	3.06
BA1832	acetyltransferase, GNAT family	NA	1.60	3.03
BA2225	acetyltransferase, GNAT family	NA	1.61	3.05
BA1264	acetyltransferase, GNAT family	NA	1.62	3.07
BA2792	acetyltransferase, GNAT family	NA	1.84	3.58
BA4868	acetyltransferase, GNAT family	NA	1.79	3.47

SpxA2 upreguated genes ≥3-fold: Cell division & sporulation

Locus	GeneN	GeneS	15_min	fold-change
BA4724	germination protein GerE	gerE	2.64	6.25
BA5521	stage III sporulation protein D	spoIIID	1.89	3.70
BA4629	prespore-specific transcriptional regulator rsfA, putative	NA	1.81	3.50
BA3930	stage III sporulation protein E	NA	1.62	3.08
