# Supplementary material for: Transcriptomic and phenotypic analysis of paralogous spx gene function in Bacillus anthracis Sterne
Source: Microbiologyopen. 2013 Jul 22;2(4):695–714. doi: 10.1002/mbo3.109 (PMC3831629; doi:10.1002/mbo3.109)
Supplement: Supplementary file 7 — Table S5. SpxA1- and SpxA2- controlled genes shown to be induced by peroxide treatment (Pohl et al. 2011). [file mbo30002-0695-SD7.docx]

Table S5. SpxA1 and SpxA2 genes shown to be induced by peroxide treatment (Pohl et al., 2011).

**SpxA1-activated genes induced by peroxide**

BA1951 Nitroreductase family protein

BA0534 Putative iron compound ABC transporter, permease protein

BA3877 a/b hydrolase fold protein of unknown function

BA1263 Class I pyridine nucleotide-disulphide oxidoreductase, putatively involved

in disulphide formation

BA1209 Putative oxygen-binding heme protein

BA1880 NRAMP (natural resistance-associated macrophage protein) family

protein, putative manganese transporter

BA2279 Glycine betaine/L-proline ABC transporter, ATP-binding protein for

osmoprotection

BA1858 Major facilitator family transporter

BA0533 Putative iron compound ABC transporter, permease protein

BA0532 Putative iron compound ABC transporter, ATP-binding protein

BA0535 Putative iron compound channel protein

BA3432 Transketolase

BA1208 Putative disulphide oxidoreductases with a thioredoxin fold

BA3433 Glucose-6-phosphate 1-dehydrogenase

BA1434 D-Isomer-specific 2-hydroxyacid dehydrogenase family protein

BA2280 Glycine betaine/L-proline ABC transporter, permease protein for

osmoprotection

BA1196 MATE efflux family protein, putative MDR efflux protein

BA3431 6-Phosphogluconate dehydrogenase family protein

BA1767 Fumarate hydratase, class II

BA4757 UvrC Excinuclease ABC, C subunit

BA3430 Transaldolase, putative

BA0197 ProI Pyrroline-5-carboxylate reductase, proline metabolism

BA1556 Methylglyoxal synthase

BA2289 Aldehyde dehydrogenase family protein for the detoxification of aldehydes

BA4541 HrcA Heat-inducible transcription repressor HrcA

BA1558 BshA L-Malic acid glycosyltransferase, involved in bacillithiol synthesis

BA0077 CtsR Transcriptional regulator

BA0196 YtbE Aldo/keto reductase family oxidoreductase

BA1296 YwdH Aldehyde dehydrogenase

BA0080 ClpC AAA superfamily domain protein, negative regulator of genetic

competence

BA5487 YwqA Putative SNF2 family helicase

BA1881 YbfQ Putative rhodanese domain sulphurtransferase for the formation of

prosthetic groups in iron–sulphur proteins

BA5411 YwjA MsbA-like ABC transporter protein

BA0078 McsA Modulator of CtsR repression with UvrB/UvrC motif

BA5714 YycG/WalK Sensory histidine kinase, cell wall metabolism

BA5335 EstA Carboxylesterase

BA4058 BshC Cysteine-adding enzyme required for the synthesis of bacillithiol

BA5713 YycH Putative regulator of YycG/WalK

BA0079 McsB ATP:guanido phosphotransferase domain protein, modulator of CtsR

repression

BA2172 Putative thioredoxin

BA1555 DapB Dihydrodipicolinate reductase

BA1515 YpdA Pyridine nucleotide-disulphide oxidoreductase family protein

BA5387 TrxB Thioredoxin reductase

BA3876 Phosphoglycerate mutase family protein

BA1141 AddB ATP-dependent nuclease, subunit B

BA1559 CnB/Cca RNA nucleotidyltransferase/poly(A) polymerase

BA5155 PepA Cytosolic protein-degrading aminopeptidase

BA3524 BshB2 N-Acetylglucosamine-malate deacetylase, involved in bacillithiol synthesis

BA4499 SodA-1 Superoxide dismutase, Mn/Fe

SpxA2-activated genes induced by peroxide

BA0533 Putative iron compound ABC transporter, permease protein

BA1858 Major facilitator family transporter

BA1208 Putative disulphide oxidoreductases with a thioredoxin fold

BA1880 MntH NRAMP (natural resistance-associated macrophage protein) family

protein, putative manganese transporter

BA0534 Putative iron compound ABC transporter, permease protein

BA2279 ProV-1 Glycine betaine/L-proline ABC transporter, ATP-binding protein for

osmoprotection

BA1263 Class I pyridine nucleotide-disulphide oxidoreductase, putatively involved

in disulphide formation

BA0535 Putative iron compound channel protein

BA0532 Putative iron compound ABC transporter, ATP-binding protein

BA3432 Tkt-1 Transketolase

BA1434 D-Isomer-specific 2-hydroxyacid dehydrogenase family protein

BA2289 Aldehyde dehydrogenase family protein for the detoxification of aldehydes

BA1767 FumC Fumarate hydratase, class II

BA2280 Glycine betaine/L-proline ABC transporter, permease protein for

osmoprotection

BA3433 Zwf Glucose-6-phosphate 1-dehydrogenase

BA5387 TrxB Thioredoxin reductase

BA4757 UvrC Excinuclease ABC, C subunit

BA4541 HrcA Heat-inducible transcription repressor HrcA

BA5561 Low-molecular-weight phosphatase family protein

BA1196 MATE efflux family protein, putative MDR efflux protein

BA3430 Transaldolase, putative

BA0197 ProI Pyrroline-5-carboxylate reductase, proline metabolism

BA3946 RibC Riboflavin biosynthesis protein RibC

BA4058 BshC Cysteine-adding enzyme required for the synthesis of bacillithiol

BA1881 YbfQ Putative rhodanese domain sulphurtransferase for the formation of

prosthetic groups in iron–sulphur proteins

BA3431 6-Phosphogluconate dehydrogenase family protein

BA0196 YtbE Aldo/keto reductase family oxidoreductase

BA1141 AddB ATP-dependent nuclease, subunit B

BA0079 McsB ATP:guanido phosphotransferase domain protein, modulator of CtsR

repression

BA1951 Nitroreductase family protein

BA0080 ClpC AAA superfamily domain protein, negative regulator of genetic

competence

BA2171 YpgR HEAT-like repeat domain of the Nfu/NifU family protein involved in the

assembly of iron–sulphur clusters

BA1555 DapB Dihydrodipicolinate reductase
