# Supplementary material for: Transcriptomic and phenotypic analysis of paralogous spx gene function in Bacillus anthracis Sterne
Source: Microbiologyopen. 2013 Jul 22;2(4):695–714. doi: 10.1002/mbo3.109 (PMC3831629; doi:10.1002/mbo3.109)
Supplement: Supplementary file 8 — Table S6. Oligonuceotide primers. [file mbo30002-0695-SD8.docx]

Table S6. Oligonucleotides primers

| **Amplified Region** | **Primer Tag** | **Primer Sequence (5’🡪3’)** | **Amplicon Size** |
| --- | --- | --- | --- |
| **Δ*spxA1* (cloned into pRP1028, pSB2)** | | | |
| upstream | SB11 | tagggGGATCCgcaaagcaagaaggttcagatg | 1 kb |
|  | SB14 | gaagcattaaaaaaaaccttacaaagctcactctccttaaagagtac |  |
| downstream | SB12 | taggcGGTACCatatgacaaatcaatagttcttgtactctc | 1 kb |
|  | SB13 | ggagagtgagctttgtaaggttttttttaatgcttcacattg |  |
| Δ*spxA1* | SB11 | tagggGGATCCgcaaagcaagaaggttcagatg | 2 kb |
|  | SB12 | taggcGGTACCatatgacaaatcaatagttcttgtactctc |  |
| **Δ*spxA2* (cloned into pRP1028, pSB3)** | | | |
| upstream | SB15 | tagggAAGCTTgaggcgcgtattgcctcaacctat | 1 kb |
|  | SB35 | ggagtgagtgaaactaagtaatggtatgagagag |  |
| downstream | SB16 | taggcGGTACCacgttcttgattgataagatcaac | 1 kb |
|  | SB36 | ctcataccattacttagtttcactcactcctttaatg |  |
| Δ*spxA2* | SB15 | tagggAAGCTTgaggcgcgtattgcctcaacctat | 2 kb |
|  | SB16 | taggcGGTACCacgttcttgattgataagatcaac |  |
| **ICE*Bs1*::P_spank(hy)_-*spxA1*DD or –*spxA2*DD (cloned into pJMA402)** | | | |
| *spxA1DD* / *spxA2DD* cloned into pJMA402 | Spac-up | gactttatctacaaggtgtg | 2.1 kb |
|  | Spac-down | aaatgatgacctcgtttcca |  |
| *spxA1DD* (pDR111) | MN10-535 | cggaagctttaaggagagtgagctttgt | 400 bp |
|  | MN10-536 | tgcatgcttaatcgtcaagacgatgtgcttcacg |  |
| *spxA2DD* (pDR111) | MN10-537 | cagaagcttaaaggagtgagtgaaacatg | 400 bp |
|  | MN10-538 | tgcatgcttaatcgtccaacttttgcagttcaat |  |
| **Direct Activation Studies and Protein Purification** | | | |
| P_BAS1811_  (pDG793) | SB50 | tagggGAATTCgtaggaaataaaaaggtaaaatatgtgagtgc | 167 bp (157 nt)^1^ |
|  | SB51 | taggcGGATCCcttttgccatcataattccctcc |  |
| P*_racE-1_*  (pDG793) | SB52 | tagggGAATTCcaccgagatacgaatgagg | 246 bp (205 nt)^1^ |
|  | SB53 | taggcGGATCCctagcacacctattactgaatgtttatgacata |  |
| P*_spxA1_*  (pDG793) | SB54 | tagggGAATTCctgtatatttcttgcaaaatctattgtataatg | 213 bp  (205 nt)^1^ |
|  | SB55 | taggcGGATCCgttaccatacaaagctcactctcc |  |
| P*_yvrH_*  (pDG793) | SB56 | tagggGAATTCctggctcttgcaactaacgttaatag | 267 bp  (218 nt)^1^ |
|  | SB57 | taggcGGATCCgtaaaattgctgtttcatcatcaacgagc |  |
| P*_trxB_*  (pDG793) | SB60 | tagggGAATTCggaatatatcgtacgcagcgaacaagg | 218 bp  (199 nt)^1^ |
|  | SB61 | taggcGGATCCcttctgacacactattcactcctattcattcctacac |  |
| P*_exoA_*  (pDG793) | SB62 | tagggGAATTCaggtgttctgctgctttaacaattctgaactgctg | 625 bp  (585 nt)^1^ |
|  | SB63 | taggcGGATCCgcacgtaaaccatttacattccacgaaatgaacttcac |  |
| *spxA2* (pPROEX-1, for protein purification) | SB37 | tagggCATATGgtggttttatatacaacagcaag | 418 bp |
|  | SB45 | gcctaGGATCCttaattagccaacttttgcagttc |  |
| **RT-qPCR Studies** | | | |
| *trxB* | trxB-F1 | aaaaattggtgtaccaggtg | 108 bp |
|  | trxB-R1 | ccgccaataactacaagttc |  |
| *spxA1* | spxA1-F1 | cgaaattatggctagaggaa | 148 bp |
|  | spxA1-R1 | tggaaaacttttgaacgagt |  |
| *yjbH* | yjbH-F1 | tgtaaagattgctgggaaat | 129 bp |
|  | yjbH-R1 | attccatttgtgtgaggaag |  |
| *racE-1* | racE-1-F1 | tccgttaaaggctctagttg | 237 bp |
|  | racE-1-R1 | gcatgactatgcactttcaa |  |
| BAS1811 | BAS1811-F1 | gtggttatggaacggtttta | 114 bp |
|  | BAS1811-R1 | ttcctgaggattgttgagac |  |
| *gatB/*Yqey (GBAA4533) | gatB-F1 | agctcgtgaagtaaaacagc | 216 bp |
|  | gatB-R1 | acagcagtcatcaccttacc |  |
| *yvrG* | yvrG-F1 | ctatcaggttcaggaaatcg | 243 bp |
|  | yvrG-R1 | tgtcacgatcagtccagtta |  |
| *spxA2* | SpxA2-F3 | tgagcatccgctaatgttg | 114 bp |
|  | SpxA2-R3 | aaaaatgttcgaacgctacg |  |

^1^indicates nucleotides prior to start codon
